# Novel Cell-Penetrating Peptides Derived From Scaffold-Attachment- Factor A Inhibits Cancer Cell Proliferation and Survival

**DOI:** 10.3389/fonc.2021.621825

**Published:** 2021-03-30

**Authors:** Pavan Kumar Puvvula, Anne M. Moon

**Affiliations:** ^1^Department of Molecular and Functional Genomics, Weis Center for Research, Geisinger Clinic, Danville, PA, United States; ^2^Department of Human Genetics, University of Utah, Salt Lake City, UT, United States; ^3^The Mindich Child Health and Development Institute, Hess Center for Science and Medicine at Mount Sinai, New York, NY, United States

**Keywords:** hnRNPU, epigenetics, cell-penetrating peptides, splicing, cancer, RGG domain, SAP domain

## Abstract

Scaffold-attachment-factor A (SAFA) has important roles in many normal and pathologic cellular processes but the scope of its function in cancer cells is unknown. Here, we report dominant-negative activity of novel peptides derived from the SAP and RGG-domains of SAFA and their effects on proliferation, survival and the epigenetic landscape in a range of cancer cell types. The RGG-derived peptide dysregulates SAFA binding and regulation of alternatively spliced targets and decreases levels of key spliceosome proteins in a cell-type specific manner. In contrast, the SAP-derived peptide reduces active histone marks, promotes chromatin compaction, and activates the DNA damage response and cell death in a subset of cancer cell types. Our findings reveal an unprecedented function of SAFA-derived peptides in regulating diverse SAFA molecular functions as a tumor suppressive mechanism and demonstrate the potential therapeutic utility of SAFA-peptides in a wide range of cancer cells.

## Background

Scaffold-attachment-factor A (SAFA, also called hnRNPU) belongs to the hnRNP family of proteins and functions in diverse processes such as epigenetic regulation, transcription, alternative splicing, translation, and mRNA stability (1–4). SAFA possesses both RNA and DNA binding activities. SAFA-mediated transcriptional activation and repression are the result of its association with p300 (5) and CBX5 (6), respectively. SAFA also participates in transcription by directly associating with BRG1 complex (7) and/or with core-TFIIH complex (8). It plays a structural role in nuclear organization by selectively tethering chromatin loops to the nuclear matrix (9, 10). SAFA binds to both coding and non-coding transcripts and functions as a global splicing regulator (3) and enhances the stability of some transcripts by binding to their 3′ UTR (11). Cellular studies have implicated SAFA as a master regulator of cell proliferation (12, 13) and cellular senescence (14). In X-chromosome inactivation, the RGG domain mediates recruitment and layering of the *Xist* molecule on the X-chromosome (15, 16).

With such diverse functions it is not surprising that SAFA has key roles in development and disease (17–19). *SAFA* variants are associated with central nervous system, cardiac, and renal anomalies (20). Its contribution to the pathogenesis of a variety of cancers is emerging. SAFA stabilizes *LIMD1* mRNA by interacting with LIMD1-AS1 to suppress non-small cell lung cancer progression (21). SAFA associates with DIS3-like 3′–5′ exoribonuclease 2 to promote hepatocellular carcinoma cell progression via SAFA-mediated alternative splicing (22). A SAFA/HNF4A-AS1/CTCF axis drives neuroblastoma progression (23). More recent data show that SAFA plays an essential role in telomere maintenance, 3D organization of interphase chromatin, chromosome positioning, and dynamic epigenetic landscape (24, 25). These collective findings provide a compelling rationale for developing agents targeting SAFA as cancer therapy.

Cell-penetrating peptides are short stretches of amino acids which allow translocation of cargo molecules across cell membranes (26). This strategy has been employed to deliver dominant-negative peptides that abrogate the function of oncoproteins Myc and ATF5, now in clinical trials (27–35). In addition to these targets, numerous peptides have been developed with effects on gastric and colon cancers (36), breast cancer (37), glioma (38), and skin cancer (39). Based on these promising results, the U.S. Food Drug Administration has recently authorized 15 different peptides (7% of all drugs approved from 2015 to 2019), reflecting the intense drug discovery efforts employing this strategy by industry and academia (40).

SAFA possesses both RNA and DNA binding activities conferred by the RGG (arginine-glycine-glycine) domain and the SAP (SAF-A/B acinus, and PIAS) domains, respectively (41–43). Senescence is a key tumor suppressor mechanism (44) and loss of SAFA reduces cell proliferation and induces premature senescence in human fibroblasts (14). Thus, we reasoned that further exploration of SAFA loss-of-function could yield novel cancer therapeutic strategies. We developed SAFA-derived cell-penetrating peptides to interrogate the mechanism(s) of SAFA-mediated functions in cancer cells and to identify dominant-negatives that phenocopy the decreased proliferation and altered gene expression that result from loss of SAFA. We employed Penetratin peptide to deliver the SAP and RGG domains and show widespread effects on cancer hallmarks and the epigenetic and transcriptional landscapes of multiple cancer cells. These results establish significance of SAFA and the efficacy of dominant-negative SAFA domains in cancer.

## Materials and Methods

### Cell Culture

T47D, MDA-MB231, CRL2327, HFF1, MCF10A, UMUC3, HCT116, DU145, and HT1080 were obtained and maintained as per the procedures mentioned in ATCC.

### Antibodies

R-IgG (SC-2027), m-IgG (SC-2025), Actin (SC-47778), H3K9me3 (Cell Signaling, 9754), H3K4me3 (Cell Signaling, 9751; active motif 39159), H3K27me3 (Cell Signaling, 9733), H3K9ac (Cell Signaling, 9649), H3K36me (Cell Signaling, 4909), H3K27ac (ab4729), H3K9ac (ab176916), rabbit polyclonal Ki67 (Vectorlabs), MLL1 (Active motif, 61296), Lamin A/C (E-1), hnRNPC1/C2 (Santa Cruz, SC-32308), SAFA (Santa Cruz, SC-32315), U2AF65 (Santa Cruz, SC-53942), DDX3 (Santa Cruz, SC-365768), hnRNPA1 (Santa Cruz, SC-32301), hnRNPD (abcam, ab61193), DDX21 (Santa Cruz, SC-376953), DNA Damage antibody sample kit (Cell Signaling, 9947), Apoptosis Antibody sampler Kit (Cell signaling, 9915).

### Protein Extraction and Immunoprecipitations (IPs)

Immunoprecipitations were performed as previously described (45).

### Immunoblotting

Immunoblotting were performed as previously reported (46). Briefly, whole-cell lysates or immunoprecipitated samples were separated by 4–20% precast BioRad gels (BioRad). PAGE separated proteins were transferred to the PVDF membrane by Mini Trans-Blot cells (BioRad) as per the manufacturer protocol. Protein-bound PVDF membranes were further sequentially incubated in a blocking buffer followed by primary (protein specific as mentioned in the figures) and secondary antibodies (anti-rabbit or anti-mouse HRP conjugate) for 2 h each. ECL Plus Western Blotting detection system (GE Healthcare) was used to reveal the protein of interest.

### Crystal Violet Assay/Optical Density Method of Cell Quantitation

Cell viability was measured by crystal violet staining as per the manufacturer's protocol (Abcam: ab232855). Three independent wells represent each point on the curve.

### RNA Isolation and Reverse Transcription–PCR Analysis

Total RNA was prepared as per the manufacture's protocol. We used “RNeasy Mini Kit” (Cat.No:74104) from Qiagen to extract the cell's RNA. cDNA is prepared from the RNA using EcoDry Premix Double Primed (Clontech) kits to convert the RNA to cDNA. Quantitative RT-PCR was performed by using SsoFast Evagreen Supermix (Bio-Rad) as per the manufacturer's protocol. PCR amplicons are separated on 3% agarose gels to detect alternative spliced isoforms. Images were captured using the ChemiDoc XRS + system (BioRad). Densitometric analysis was performed on long and short isoform-specific amplicons of the spliced targets using ImgaeJ software to calculate the ratio between exclusion and inclusion.

### Chromatin Immunoprecipitation (ChIP)

Chromatin Immunoprecipitation was carried out as per the manufacturer's protocol (9003S, Cell Signaling).

### siRNA Knockdown

Cells are transfected with control or SAFA specific siRNAs (14) using X-treme GENE HP DNA transfection reagent (Roche, 6366244001) as per manufacturer's instructions.

### Cell Counts for Cell-Penetrating Peptide (CPP) Treatments

Cells were seeded in six-well dishes and incubated with 10 μM concentration of peptides in Opti-MEM reduced serum media for the indicated times. Cell counts were measured by using a hemocytometer.

### Generation of Synthetic Peptides

Lifetein synthesized SAFA-derived peptides at purity >75%. Peptides were dissolved in RNase/DNase free MilliQ water at 2 mg/ml and used at a concentration of 10 μM in Opti-MEM media.

RT-PCR and ChIP-PCR primer sequences are available in Table 1 (Supplementary Material).

### Annexin V-FITC Apoptosis Detection

Activation of apoptosis was measured by Annexin V/PI dual staining by using Annexin V-FITC apoptosis detection kit (ab14085) from Abcam. Seventy percent of confluent cells were treated with 10 μM of SAFA-derived peptides for 12 h in Opti-MEM reduced media. Apoptosis detection assay was performed as per the manufacture's protocol. The stained cells were captured by confocal microscopy.

Immunofluorescence was performed as previously published (47).

### UV-Crosslinked RNA Immunoprecipitation (CLIP)

CLIP was performed as previously described (48). Briefly, confluent cells were treated with SAFA-derived peptides for 12 h in reduced serum media. Cells were washed in PBS and crosslinked in Stratalinker at the rate of 400 mj/cm^2^ at 254 nm wavelength. Lysates were prepared by using NP-40 lysis buffer (50 mM Tris HCl, pH 7.4, 150 mM NaCl, 1 mM MgCl_2_, 0.05% NP-40, 1 mM DTT, 2 mM EDTA, 100 U/ml RNasin, and Protease inhibitor cocktail). Cleared lysate was immunoprecipitated by anti-SAFA and R-IgG antibodies. Enriched protein complexes were subjected to Proteinase K digestion at 37°C for 1 h, followed by RNA isolation using phenol/chloroform extraction and ethanol precipitation.

## Results

### SAFA-Derived Cell-Penetrating Peptide Reduces Proliferation of Human Fibroblasts

**Figure 1:**
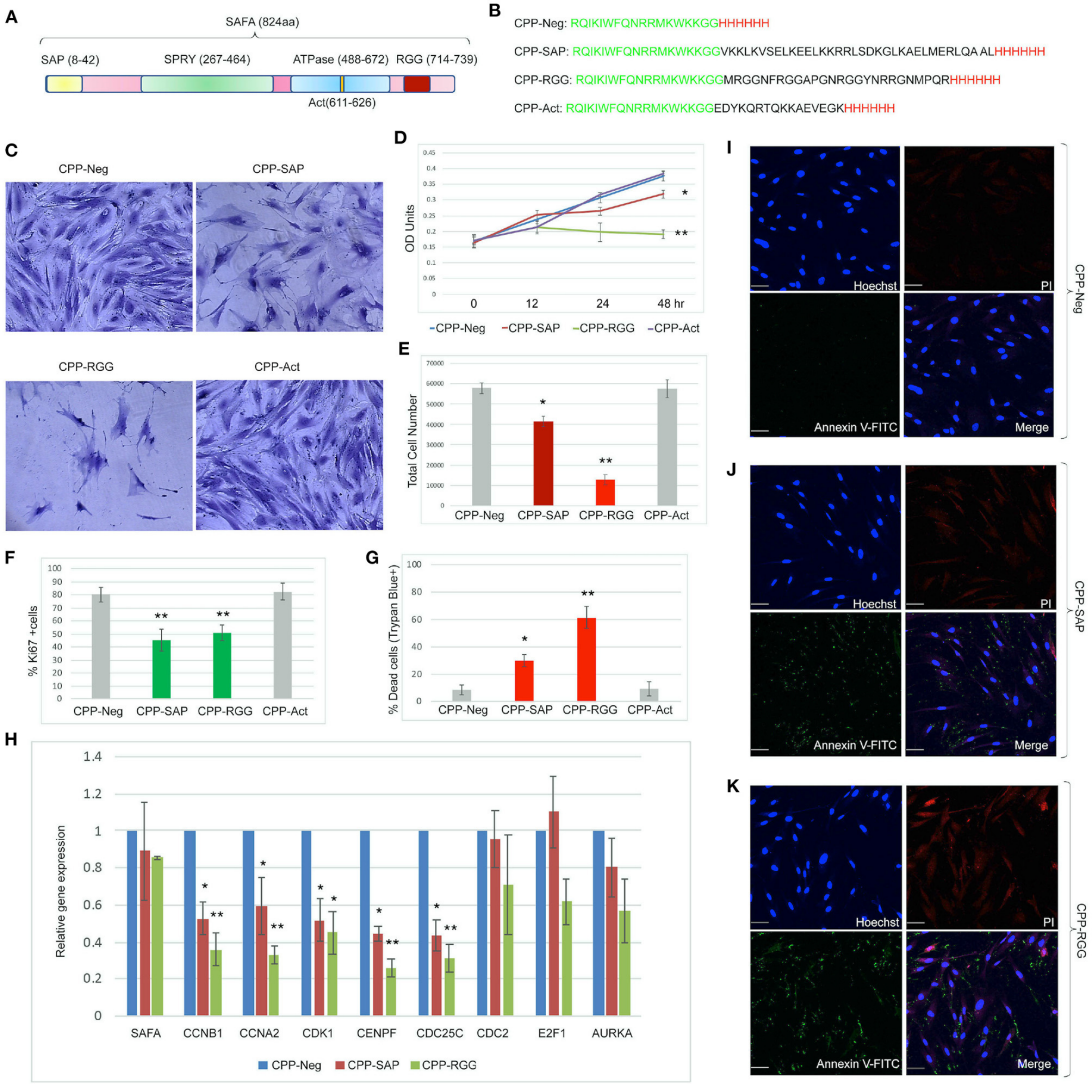
Cell-penetrating SAFA-derived peptides reduce proliferation of primary human fibroblasts. **(A)** Schematic of SAFA protein domains. Numbers in the brackets indicate the amino acids in each domain. **(B)** Sequences of the cell-penetrating peptides CPP-Neg, CPP-SAP, CPP-RGG, and CPP-Act-His. The sequence of Penetratin is in green, his tag in red and SAFA domains in black. **(C)** Representative light microscopic images of crystal violet-stained human foreskin fibroblasts treated for 48 h with 10 μM concentration of SAFA-derived peptides (listed at top). **(D)** Crystal violet assay of HFFs treated with CPP-Neg, CPP-SAP, CPP-RGG, and CPP-Act peptides over the course of 48 h. **(E)** Quantification of total cell number of HFFs after 24 h of treatment. **(F, G)** Quantitation of Ki67^+^ cells **(F)** and -percent dead trypan blue stained cells **(G)** after 24 h of peptide treatment. **(H)** qRT-PCR analysis of cell cycle gene transcripts in total RNA isolated after 24 h of peptide treatment. **(I–K)** Representative immunofluorescence images of Annexin V/PI stained HFFs after 24 h treatment [**(I)**, CPP-Neg; **(J)**, CPP-SAP; **(K)**, CPP-RGG]. Individual channels (Hoechst = Blue, Annexin V = Green, PI = Red) and merged images are shown. Scale bar, 50 μm. Error bars represent standard deviation. *p < 0.05, **p < 0.01 relative to control.

We previously demonstrated that the SAFA RGG domain facilitates the interaction of SAFA and Polycomb complex to regulate expression of pro-proliferation genes (14). We hypothesized that the RGG and SAP domains could have dominant-negative functions that would induce SAFA loss-of-function on cell survival, proliferation, and gene expression. We added Penetratin and a his6-tag to the RGG and SAP domains (Figures 1A,B). We also generated a peptide derived from the SAFA actin dimerization domain because it plays a vital role in SAFA-actin complex formation and transcription. These engineered peptides are henceforth referred to as CPP-RGG, CPP-SAP, CPP-Act: the N-terminal Penetratin domain mediates cellular penetration (49) and the C-terminal his6-tag acts as a detection signal. A his6-tagged Penetratin peptide serves as a negative control (CPP-Neg). Analysis of human primary fibroblasts treated with 10 μM peptide showed that all four peptides entered the nucleus within 4 h of treatment (Supplementary Figures 1.1B–E, negative controls Supplementary Figures 1.1F–J). Quantitation of cell number and viability showed that treatment with CPP-RGG markedly decreases proliferation, total cell number, and Ki67+ cells while the effect of CPP-SAP was more modest (Figures 1D–F). In contrast, CPP-Act had no effect on any of these parameters and we did not pursue this peptide further (Figures 1C–G, Supplementary Figure 1.2). In addition to their effects on proliferation, CPP-RGG and CPP-SAP significantly increased cell death (Figure 1G). SAFA depletion decreases expression of E2F-responsive genes (14) and qRT-PCR revealed decreased transcripts of such genes in CPP-RGG and CPP-SAP treated fibroblasts (Figure 1H). CPP-SAP treated fibroblasts displayed an increased number of Annexin V positive, propidium iodide (PI) negative cells, suggestive of early apoptosis (Figures 1I,J) while CPP-RGG treatment caused an increased number of Annexin V/PI positive cells which are late apoptotic or necrotic cells (Figure 1K). These results are congruent with SAFA loss-of-function and suggest dominant-negative properties of CPP-SAP and CPP-RGG.

### CPP-SAP and CPP-RGG Are Effective Against a Range of Cancer Cell Types

**Figure 2:**
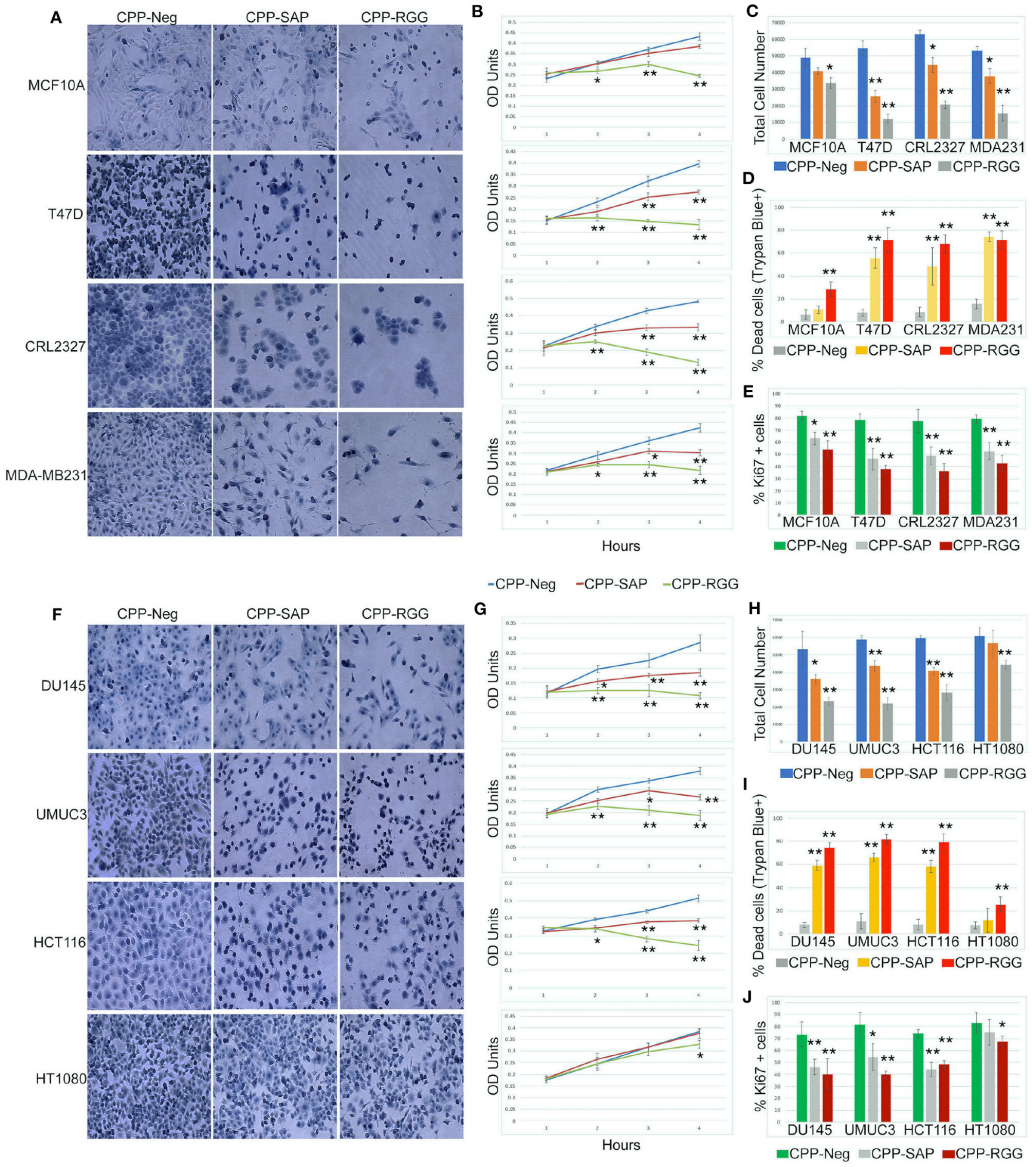
Cell-penetrating SAFA-derived peptides reduce proliferation of a wide range of cells. **(A)** Representative light microscopic images of crystal violet stained MCF10A, T47D, CRL2327, and MDA-MB231 cells treated with SAFA-derived peptides (listed at top), for 48 h at 10 μM. **(B)** Crystal violet assay of MCF10A, T47D, CRL2327, and MDAMB 231 cells treated with CPP-Neg, CPP-SAP, and CPP-RGG peptides over the course of 48 h (1= 0, 2 = 12, 3 = 24, 4 = 48). **(C–E)** Quantification of total cell number, dead cells as measured by trypan blue staining and Ki67^+^ cells after 24 h of CPP-Neg, CPP-SAP, CPP-RGG, and CPP-Act peptide treatments. **(F–J)** As in **(A–E)** with additional cell lines. *p < 0.05, **p < 0.01 relative to control.

SAFA is expressed in a wide range of cancer cells so we tested these peptides on breast (luminal subtypes T47D and CRL2327 and MDA-MB231 triple-negative subtype), bladder (UMUC3), colorectal (HCT116), fibrosarcoma (HT1080), and prostate (DU145) cancer cell lines. We also tested MCF10A cells, a transformed but non-malignant breast epithelial cell line and verified that the peptides enter these cells (Supplementary Figures 2.1A–G). Treatment of all of the cancer cell lines with CPP-RGG decreased cell proliferation as measured by total cell number, crystal violet OD units, and Ki67+ cells (Figures 2A–J). MCF10A and HT1080 cells were markedly less sensitive to treatment. The percentage of dead cells was >60% in all cell lines except MCF10A and HT1080 (Figures 2D,I) demonstrating that the altered growth curves reflect both decreased proliferation and increased death. Analysis of the apoptotic/necrotic response by Annexin V/PI staining showed both early and late apoptotic cells in response to CPP-RGG in MDA-MB231, HCT116, and UMUC3 cells (Supplementary Figures 2.2,2.3) while T47D and DU145 cells exhibited only increased annexin V-positive cells. In contrast, MCF10A and HT1080 cells had only modest increases in Annexin V-positive cells.

Notably, and in contradistinction to CPP-RGG, the effects of CPP-SAP were restricted to cancer cells, with minimal effects on MCF10A cells (Figures 2A–G). Although HT1080 is a fibrosarcoma line, CPP-SAP treatment did not affect their proliferation or viability and these cells were also the least sensitive to CPP-RGG (Figures 2F–I); this is consistent with the low sensitivity of fibrosarcoma to both chemo- and radiation therapies; http://www.nice.org.uk/guidance/csgsarcoma/evidence/improving-outcomes-for-people-with-sarcoma-the-manual2. CPP-SAP treatment increased Annexin V staining in T47D and DU145 cells and increased levels of both Annexin V and PI in MDA-MB231, CRL2327, HCT116, UMUC3 cells; MCF10A and HT1080 cells were again minimally affected (Supplementary Figures 2.2,2.3). Thus, the dominant-negative functions of CPP-SAP and CPP-RGG diverge in their effects on cancer cells vs. benign or chemo-resistant cells.

The cell-type specific effects on gene expression led us to ask whether peptide treatment altered cell cycle kinetics. To this end, we treated DU145, HCT116, and HT1080 cells with peptide for 24 h, stained adherent cells with PI and performed flow cytometry (Supplementary Figure 2.4); we also assayed cell cycle gene expression (Supplementary Figure 2.5). CPP-SAP and CPP-RGG reduced the percentage of DU145 cells in G1 with an increase in S phase, indicating that the peptides cause replication defects and/or DNA damage (Supplementary Figure 2.4A). Despite altered expression of multiple cell cycle genes (Supplementary Figure 2.5E), the phased distribution of cells was not perturbed in HCT116 cells (Supplementary Figure 2.4B) possibly indicating that the progression to death was rapid in this line. Peptide treatments marginally affected cell cycle gene expression in HT1080 cells (Supplementary Figures 2.4C,2.5H) consistent with their lower sensitivity to peptide in previous assays.

### CPP-SAP and CPP-RGG Alter Splicing of SAFA Targets in a Cell-Specific Manner

**Figure 3:**
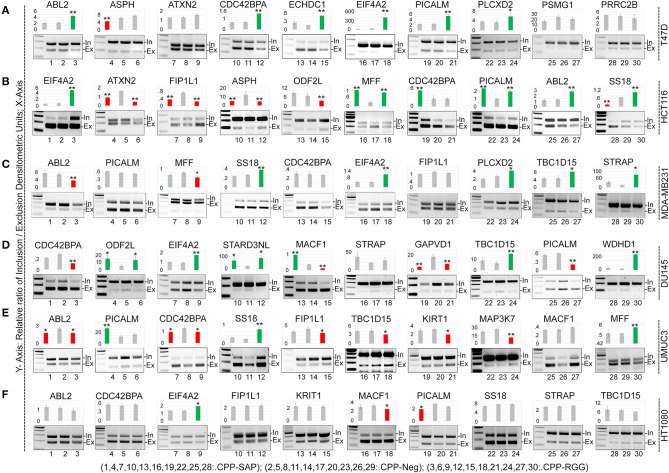
CPP-RGG and CPP-SAP dysregulate alternative splicing of SAFA-targets *in vivo* in wide range of cells. **(A–F)** RT-PCR testing of known SAFA-dependent splicing targets in T47D **(A)**, HCT116 **(B)**, MDA-MB231 **(C)**, DU145 **(D)**, UMUC3 **(E)**, and HT1080 **(F)** cells treated with CPP-Neg, CPP-SAP, and CPP-RGG peptides for 12 h. Bar graph indicates the relative ratio of cassette inclusion to exclusion as determined by densitometry. Red and green bars indicate exclusion and inclusion, respectively. Representative gel pictures are shown below the bar graphs. *p < 0.05, **p < 0.01 relative to control.

SAFA participates in exon inclusion and exclusion of a large number of transcripts via the RGG domain (3). We reasoned that the dominant-negative effect of CPP-RGG could arise from antagonism of SAFA-mediated splicing. To test this, we assayed splice variants of 20 known SAFA targets in response to peptide treatment in cancer and MCF10A cells (Figure 3, Supplementary Figures 3.1,3.2). Densitometric values of PCR amplicons were used to calculate the exon inclusion vs. exclusion ratio and are represented as bar graphs for each target; representative agarose gel pictures are also shown. While both peptides had effects on alternative splicing, this was more common and robust with CPP-RGG. There was no bias or predictable pattern toward exon inclusion or exclusion of a particular target (compare *CDC42BPA* across cell lines), with the exception of *EIF2A* which had increased ratio of inclusion to exclusion in most cell types only in response to CPP-RGG. Even the two breast cancer lines (T47D and MDA-MB231) had divergent splicing responses to peptide treatment. Once again, HT1080 was the least affected cancer cell line (Figure 3F). Overall peptide treatment alters the stoichiometry of isoforms in diverse, context-specific ways, as has been reported elsewhere for SAFA (50, 51). These data provided strong evidence for an RGG-mediated dominant-negative effect on SAFA-mediated splicing regulation.

### RGG Peptide Alters the Binding of SAFA With RNA Targets *in vivo*

**Figure 4:**
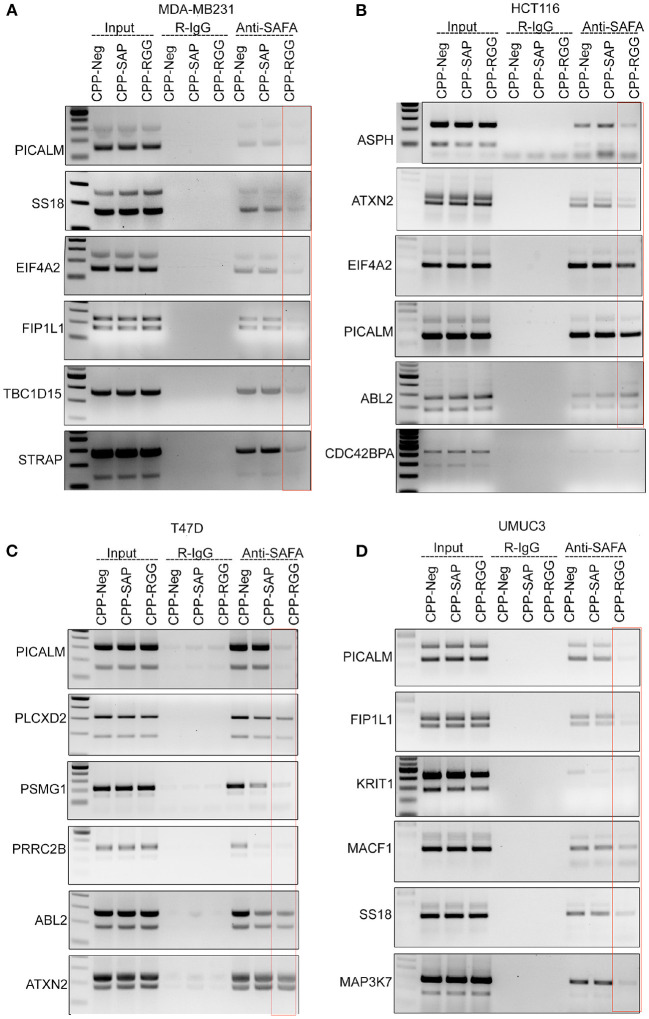
CPP-RGG and CPP-SAP disrupt endogenous SAFA association with specific mRNAs and spliceosome components. **(A–D)** Crosslinked RNA-IP (CLIP) with anti-SAFA and R-IgG followed by RT-PCR for transcript detection. Red box highlights the lack of SAFA interaction with specific transcripts in CPP-RGG treated cells. *p < 0.05, **p < 0.01 relative to control.

We next examined SAFA's interaction with its spliced targets by CLIP analysis to determine whether altered splicing in response to peptide correlated with altered SAFA RNA-binding (Figures 4A–D, Supplementary Figure 4.1). CPP-Neg. CLIP shows SAFA binding to each transcript in the cells tested. CPP-RGG markedly decreased SAFA binding to isoforms of most targets in all cell lines while CPP-SAP had variable effects as exemplified by *PICALM* (Figures 4A–D). These results are consistent with the pivotal role of the RGG domain in the RNA-binding functions of SAFA (52). In contrast, CPP-SAP-domain influences the splicing of only a few of these targets. Thus, CPP-SAP and CPP-RGG have distinct effects on alternative splicing.

### CPP-SAP and CPP-RGG Peptides Alter Levels of Spliceosome Complex Proteins and RBPs in HCT116 Cells

**Figure 5:**
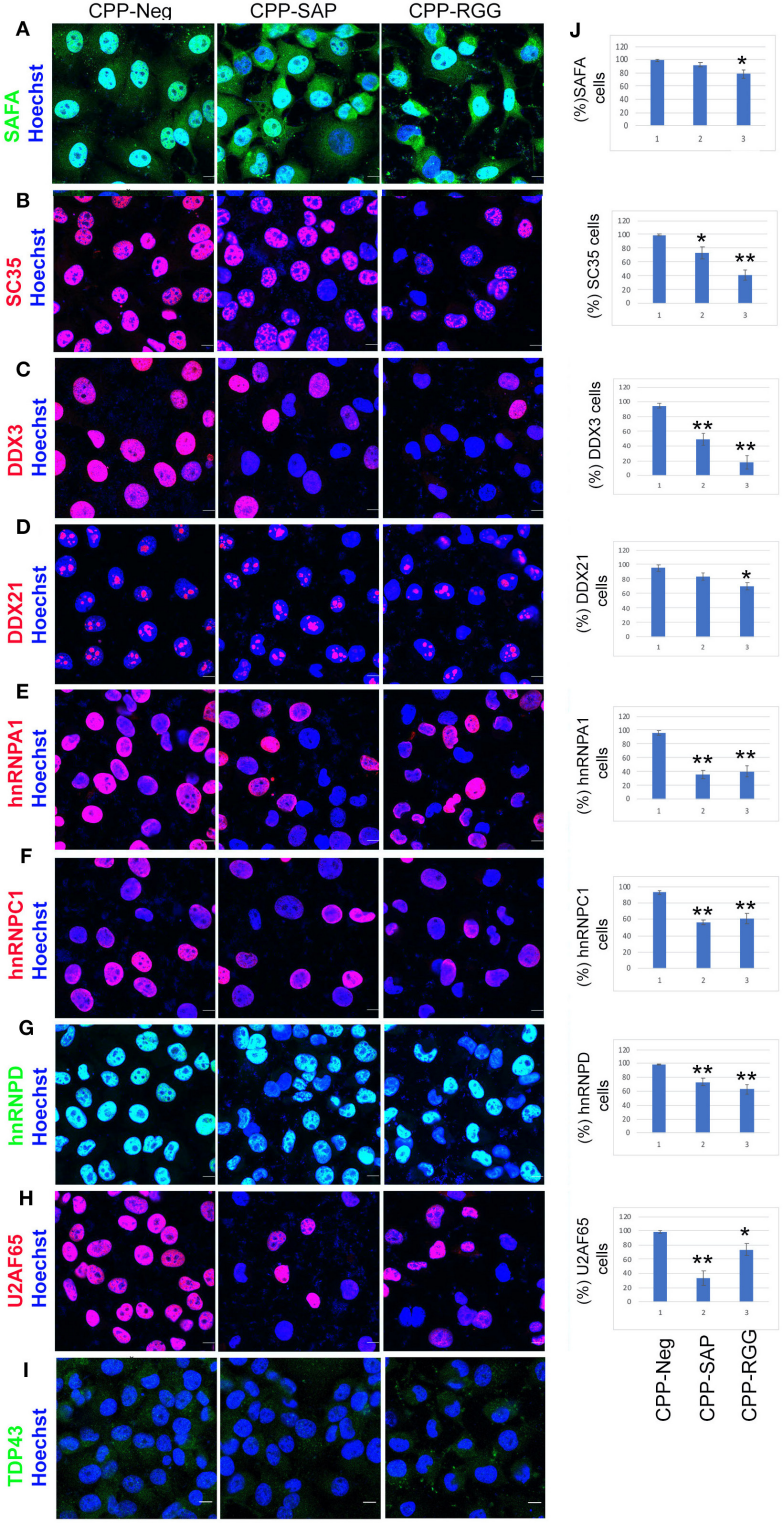
CPP-RGG and CPP-SAP decrease levels of SAFA-interacting spliceosome components. **(A–I)** Immunofluorescence for spliceosome interactors (red or green signal) and nuclei (Hoechst, blue) in peptide treated HCT116 cells. Scale bar, 10 μm. **(J)** Quantification of % of HCT116 cells positive for proteins indicated at left. *p < 0.05, **p < 0.01 relative to control.

Several spliceosome components are known to interact with SAFA (3, 53) therefore, we decided to determine the consequence of peptide treatment on the levels of SAFA-interacting RNA-binding proteins (RBP) relevant to splicing, cell cycle progression, and survival. We did these experiments in HCT116 cells because this line responded similarly to both peptides with regard to splicing (Figure 3B). We first examined the effects of peptide on amount and location of SAFA since altered levels or location could contribute to the splicing defects observed and to any alterations in its interacting partners: while CPP-SAP did not alter the SAFA levels or subcellular localization, CPP-RGG treatment caused only a modest reduction of SAFA expression in HCT116 cells (Figure 5A, Supplementary Figures 4.2.11,4.2.12). We think it is unlikely that this small decrement in SAFA levels is a significant contributor to the dramatic phenotypes observed in response to CPP-RGG.

The peptides had marked effects on the levels and localization of numerous SAFA splicing interacting proteins. SC35, a known nuclear speckle marker, was drastically reduced in response to both peptides with a decrease in SC35+ cells of >60% by CPP-RGG [Figure 5B, quantitation at right of image (Figure 5J)]. We also noted a decrease in speckle size in those cells retaining SC35. Thus, the downregulation of SC35 coincides with the splicing defects induced by the dominant-negative domains of SAFA. Loss of SC35 induces widespread alterations in splicing as well as genomic instability, and cell cycle arrest (54) as does SAFA depletion, suggesting a convergent role for SAFA and SC35 in spliceosome speckle organization and function. DDX3 and DDX21 are multifunctional Dead-box RNA helicases involved in spliceosome formation, RNA processing, and cell cycle regulation (55). Again, both peptides decreased these nuclear proteins and CPP-RGG had the most pronounced effect (Figures 5C,D). Loss of hnRNPA1, hnRNPC1, or hnRNPD induces cell death and proliferation arrest in cancer models (56–62) and these proteins were decreased in response to peptide treatment (Figures 5E–G). U2AF65 plays an instrumental role in splicing regulation and other RNA processing events (63, 64). The effect of CCP-SAP was more pronounced (>80% of cells had decreased or no U2AF65 signal, Figure 5H) than CPP-RGG. In the case of the multifunctional RBP TDP-43, CPP-SAP treatment caused a modest increase in TDP43 signal intensity while in CPP-RGG treated cells, TDP43 was redistributed as punctae in the cytoplasm in >90% of cells (Figure 5I, Supplementary Figures 4.2.15, 4.2.16). Previous reports have demonstrated such a pattern for TDP43 (65) and suggested that these punctae are localized within stress granules (66). Together these results argue that effects of SAFA-derived peptides on levels and localization of multiple key splicing factors and RBPs contribute to splicing defects as a mechanism for the decreased cell proliferation and survival in response to peptides.

### CPP-SAP and CPP-RGG Treatment Do Not Affect SAFA Chromatin Occupancy on Target Gene Promoters

We previously showed that SAFA binds to the promoters of pro-senescence genes and represses their transcription in fibroblasts (14). Deletion studies suggest that the SAP-domain plays a pivotal function in SAFA-mediated chromatin activities (67, 68). Hence, we asked whether CPP-SAP treatment disrupted SAFA's association with target promoters. We isolated SAFA-bound chromatin complexes from HCT116 and MDA-MB231 cells treated with CPP-SAP, CPP-RGG, and CPP-Neg. peptides. We choose them based on their similarly severe cell death response in the Annexin V/PI experiments (Supplementary Figures 2.2B,2.3B). Quantitative ChIP-qPCR employing a series of primer pairs that scan the promoters of a randomly selected group of known SAFA target genes detected specific association of SAFA with all the promoters at baseline. Surprisingly, there was only a modest reduction of SAFA association with three of these promoters by CPP-SAP and only in HCT116 cells (Supplementary Figure 4.3). Expression of *CDK1, CDC25C*, and *CCNB1* is markedly deceased by both peptides in fibroblasts and HCT116 cells (Figure 1H, Supplementary Figure 2.5E). So, we tested whether this was related to SAFA binding however, we did not detect SAFA occupancy at these promoters (Supplementary Figures 5A,B) in either MDA-MB231 or HCT116 cells. These results indicate that the mechanism for peptide efficacy is not based on disruption of SAFA chromatin binding.

### CPP-SAP and CPP-RGG Peptide Alter Levels and Localization of Nuclear Matrix Proteins

**Figure 6:**
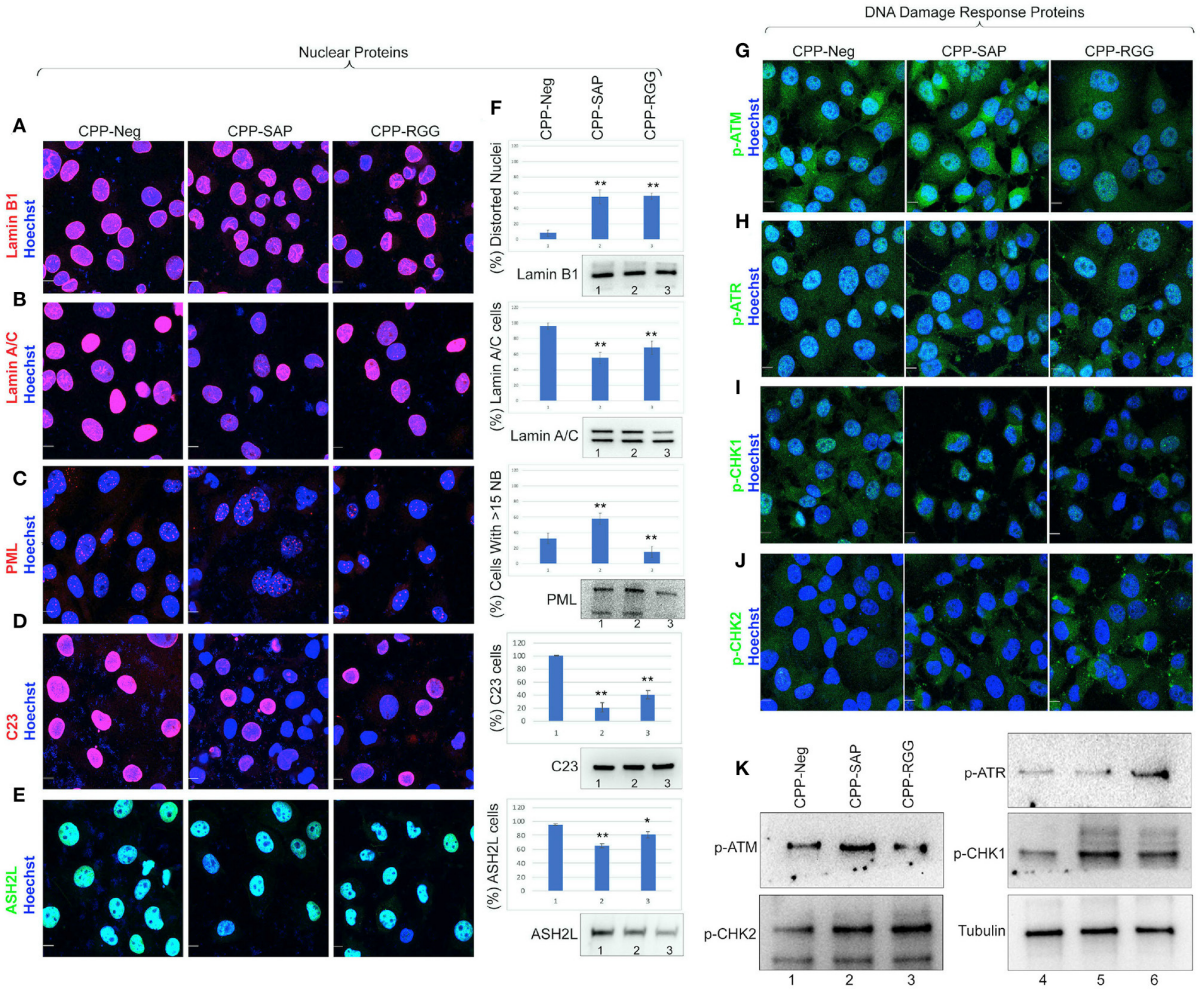
CPP-RGG and CPP-SAP disrupt nuclear architecture and nuclear protein levels and activate DDR pathways in HCT116 cells. **(A–E)** Representative immunofluorescence images for lamin B1, lamin A/C, PML, C23 (red); ASH2L (green); nuclei (Hoechst, blue) after 24 h of treatment. Scale bar, 10 μm. **(F)** Quantitation of cells for protein or nuclear feature. Immunoblots showing the endogenous protein levels. **(G–J)** Immunofluorescence for DDR effectors (green) and nuclei (Hoechst, blue) in CPP-treated HCT116 cells. Scale bar, 10 μm. **(K)** Immunoblots of p-ATM, p-ATR, p-CHK1, p-CHK2, and tubulin levels in HCT116 cells treated with SAFA-derived peptides. *p < 0.05, **p < 0.01 relative to control.

SAFA associates with the nuclear matrix and mediates tethering of higher-order chromatin loops to the nuclear matrix via scaffold/matrix attachment regions (S/MARs); the SAP domain is needed for this aspect of SAFA function (67). Since there are over 400 nuclear matrix associated proteins (69) we chose a small group of these based on SAFA interaction, representation of four different aspect of nuclear architecture and these additional criteria: (1) Lamin B1 and A/C play a key role in nuclear membrane structure and organization, spatial positioning of the genome, and global gene regulation and disruption of SAFA causes widespread remodeling of chromatin-lamina interactions at the nuclear periphery (24); (2) PML nuclear bodies associate with the nuclear matrix (70, 71); (3) SAFA loss-of-function causes a substantial reduction of C23 which, is an essential nucleolar protein of nucleolus that, when decreased, causes nucleolar disruption, increased H3K9me3 marks, and cell cycle arrest (72); (4) ASH2L and SAFA are recruited during X chromosome inactivation and function in layering of the *Xist* lncRNA and subsequent maintenance of Xi repression (16, 73). We found that both CPP-SAP and CPP-RGG lead to diminished lamin B1 in the nuclear envelope in ~50% of cells, as well as nuclear distortion despite overall maintenance of lamin B1 protein levels (Figures 6A,F). Interestingly, lamin B1 is expressed in all cells, whereas lamin A/C is restricted to differentiated cells (74) and primarily regulates nuclear stiffness (75). We scored peptide treated cells for lamin A/C levels and evaluated for the presence of distortion or other irregularities of the nuclear envelope. While we found a significant difference in lamin A/C staining in both CPP-RGG and CPP-SAP treated cells (Figures 6B,F, Supplementary Figures 6.1.13,6.1.14), abnormal nuclei were more common and severe in the CPP-SAP treated cells (Figures 6A–E). Consistent with this, the effects on PML bodies and protein levels were very different: CPP-SAP increased PML number, size, and the total amount of PML protein whereas CPP-RGG significantly reduced these features (Figures 6C,F). Both peptides caused a significant decrease in C23 intensity in the nucleus and nucleolar regions (Figures 6D,F) comparable to the effect of SAFA knockdown (24); consistent with this, nucleolar size and number increased (best visible in Figures 6C,E). This is notable because it is a marker of senescence and apoptosis (76–78). Both peptides decrease the number of ASH2L+ cells and ASH2L protein levels (Figures 6E,F). Assay of the ASH2L partner MLL1 did not show any peptide effect (Supplementary Figure 6.1.17). Thus, peptides specifically effect select nuclear proteins. All of these findings indicate diverging effects of these peptides on distinct nuclear proteins regulating multiple aspects of nuclear architecture.

We considered the marked differences in PML bodies and levels in response to CPP-SAP vs. CPP-RGG in light of reports that increased PML (seen with CPP-SAP) correlates with the DNA response (DDR) (79) while decreased PML (seen with CPP-RGG) correlates with progression to apoptosis (80). This prompted us to investigate possible modes of action in cell cycle and apoptosis. RGG-CPP caused a marked increase of cleaved caspase-7 (Supplementary Figure 6.2C), but not cleaved caspase-3 or−9 (Supplementary Figures 6.2A,B). Analysis of DDR markers revealed increased levels of p-ATM and its substrate pCHK2 by CPP-SAP (Figures 6G,J,K). Conversely, CPP-RGG treatment increased p-ATR and its downstream target p-CHK1 (Figures 6H,I,K). A surprising finding was an increase in p-CHK1 in the cytoplasm of CPP-SAP treated cells (Figure 6I) and p-CHK2 in the cytoplasm of CPP-RGG treated cells. Cytoplasmic functions for these proteins in cellular metabolism and homeostasis are emerging (81). In total these findings indicate complex and differential cellular stresses induced by CPP-SAP vs. CPP-RGG treatment.

### CPP-SAP Induces Heterochromatinization in a Cell-Specific Manner

**Figure 7:**
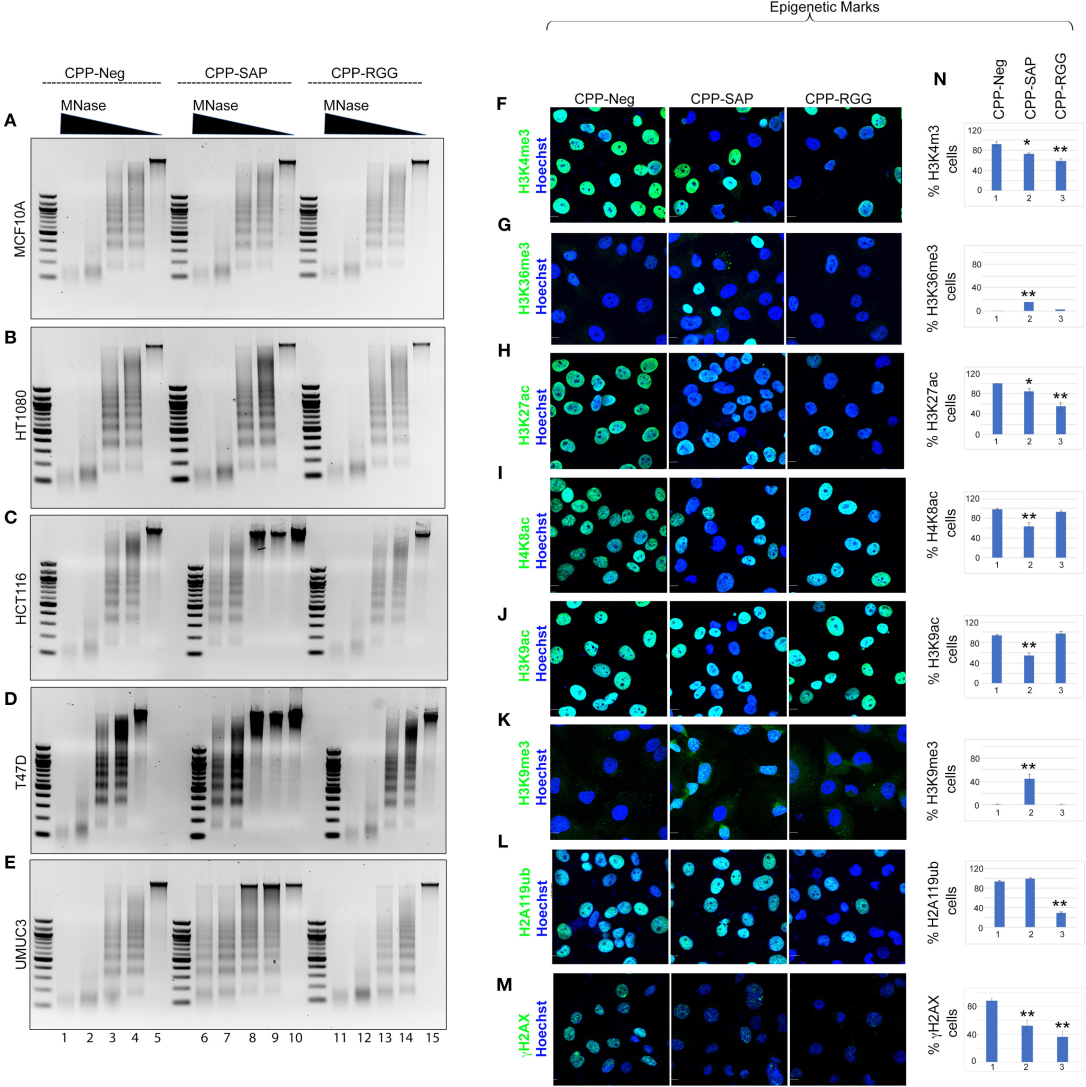
CPP-RGG and CPP-SAP alter chromatin organization and epigenetic marks in HCT116 cells. **(A–E)** Agarose gel images of MNase digested chromatin from cells pretreated with SAFA-derived peptides for 24 h. Relative MNase concentration gradient at top. **(F–M)** Immunofluorescence analysis of histone marks in treated HCT116 cells. Scale bar, 10 μm. **(N)** Quantification of % cells positive for the indicated histone epigenetic marks in response to peptide treatment. *p < 0.05, **p < 0.01 relative to control.

We previously demonstrated that silencing SAFA in human primary fibroblasts results in alteration of chromatin structure with increases in senescence-associated heterochromatin foci (SAHFs) and in marks of transcriptionally silent chromatin (H3K9me2/3, H3K27me3, H2A119ub; 14). We reasoned that SAFA-derived dominant-negative peptides would similarly disrupt higher-order chromatin structure in cancer cells and so we isolated native chromatin from a selection of cancer and benign cells and digested with micrococcal nuclease (MNase) to assess the effects of peptide treatment. As in previous experiments, MCF10A and HT1080 cells were unaffected by peptides, evident by the virtually identical MNase ladders between control and CPP-SAP or CPP-RGG peptides (Figures 7A,B). In contrast, chromatin structure in HCT116, T47D, and UMUC3 cancer cells was markedly compacted by CPP-SAP treatment, but not CPP-RGG (Figures 7C-E). This nuclease resistance was previously reported in response to SAFA knockdown in AML12 hepatocytes (24). From these results, we infer that SAFA facilitates active chromatin hubs in cancer cells and conclude that CPP-SAP leads to heterochromatinization which predicts widespread repression of gene expression that likely contributes to CPP-SAP mediated effects on proliferation and death of cancer cells. This is another example of differential effects of CPP-SAP and CPP-RGG and are consistent with the DNA-binding function of the SAP domain.

### SAP and RGG Peptides Induce Wide Spectrum of Alterations in Global Epigenetic Marks

SAFA interacts directly with active (H3K4me3, H3K36me, H3K9ac) and repressive (H3K27me) histones (14). The increase in heterochromatin in response to CPP-SAP would be expected to correlate with globally altered histone marks. We tested this in HCT116 cells and found that H3K9me3 repressive marks were increased by CPP-SAP (Figures 7K,N) while most active marks were decreased (H3K4me3, H3K27ac, H4K8ac, H3K9ac, Figures 7F–J,N). CPP-RGG decreased some active marks but had no effect or minimal effects on repressive marks with the exception of H2A119ub (Figures 7L,N, Supplementary Figures 7.11,7.12). The high levels of γH2AX in these cells at baseline (Figure 7M, CPP-Neg) (82, 83) was markedly decreased by both CPP-SAP and CPP-RGG; in the CPP-SAP treated cells, this finding is consistent with the fact that γH2AX requires relaxed chromatin to bind and recruit other DDR proteins to generate H2AX foci. The explanation for the decrement of γH2AX in the CPP-RGG cells is not clear. Previously we reported that PRC1 complex interacts with SAFA in a lncRNA dependent manner via the RGG domain and silencing of SAFA reduces global H2A119ub marks (14). Consistent with this, CPP-RGG treatment phenocopied SAFA loss-of-function and showed reduced H2A119ub signal intensity as well as loss of the H3K4me3 epigenetic mark catalyzed by the Trithorax complex (ASH2L, MLL1, RbBP5, and WDR5, Figures 7A,N). SAFA and ASH2L are part of the X-chromosome inactivation machinery, and the RGG domain is required for SAFA recruitment by *XIST* RNA. Altogether, these experiments suggest divergent roles for the SAP and RGG domains in SAFA-mediated chromatin organization and global epigenetic regulation.

## Discussion

SAFA is a multimodular protein involved in the structural organization of nuclear matrix (42), genome integrity (84), transcription regulation (8), alternative splicing (3), and mRNA stability (11). Alterations in SAFA levels and function are linked to numerous diseases, including cancer and to cardiovascular, neurological, and developmental disorders (4, 17, 18, 20, 85). Recent reports suggest that SAFA plays an instrumental role in cell fate decisions by regulating gene expression of coding and non-coding genes (14, 86). Despite its importance, mechanistic understanding of SAFA in cancer cell behavior is still primitive and it has not been probed as a therapeutic target. We demonstrate the therapeutic potential of SAFA-derived cell-penetrating peptides from the DNA (SAP) and RNA (RGG)-binding domains on a wide range of cancer cells.

Our main finding is the functional divergence of SAFA-derived peptides in controlling multiple key cellular processes in normal vs. cancer cells which suggests the potential for SAFA-based treatments via these novel therapeutic molecules. Treatment of cancer cells with SAFA-derived peptides significantly dysregulated the transcripts and isoforms of multiple genes involved in neoplastic transformation, apoptosis, cell cycle regulation, and proliferation in a peptide-specific manner. CPP-SAP had marked effects on proliferation, growth and the epigenetic landscape by driving chromatin compaction and global loss of active histone marks in cancer cells; benign (MCF10A) or treatment-resistant cancer cells (HT1080) were un- or minimally affected. In contrast, CPP-RGG had effects on proliferation, survival, and splicing/splicing machinery in all cell types; combined with its very minimal effects on the epigenetic landscape, these findings indicate that SAFA's RNA-binding and processing functions are essential for cell survival. These peptides also had divergent effects on nuclear architecture and levels of DDR effectors. These different modes of action offer distinct opportunities for targeted therapies.

The dramatic alteration of chromatin structure and histone marks in cancer cells in response to CPP-SAP are consistent with previous work showing that the depletion of SAFA itself leads to chromatin compaction and loss of long-range chromatin interactions (24). CPP-SAP treatment leads to chromatin compaction by increasing heterochromatin marks, suppressing the expression of proliferation-promoting genes, and inhibiting cancer cell growth. Expanded euchromatin is a hallmark of the epigenetic landscape in cancer so the resistance of MCF10A and HT1080 cells to CPP-SAP-mediated chromatin compaction underscores the importance of SAFA in establishing active chromatin environment in cancer cells and provides additional incentive to pursue this peptide for clinical purposes. RGG-derived peptide reduces global H3K4me3 marks, likely due to the association of SAFA with ASH2L, a part of the Trithorax histone methyltransferase complex (73). Combined with data showing that SAFA and ASH2L are critical for X-chromosome inactivation and the requirement for the RGG domain for SAFA's association with *XIST* RNA (15), our data support important functional crosstalk between SAFA and ASH2L in regulating the H3K4me3 mark. We show profound effects of these peptides on nuclear architecture and organelles and divergent effects on PML and PML bodies: CPP-SAP markedly increased levels of PML protein and number of PML bodies which is notable since PML overexpression is associated with the DDR, repressive histone epigenetic marks, altered cell cycle blockade, and induction of senescence (87). This notion is concordant with our finding of elevated levels of p-ATM and its downstream target p-CHK2.

CPP-RGG peptide treatment disrupts SAFA-mediated splicing and the splicing machinery in all cell lines tested suggesting that RNA association of SAFA is essential for alternative splicing, spliceosome complex dynamics, and RBP interactions. *In vivo* RNA-binding analyses (CLIP assays) indicate that CPP-RGG sequesters SAFA's RNA-binding activity and functions as a splicing antagonist in a wide range of cells. There may be other mechanisms underlying its effects on splicing, such as altered protein-protein interactions and activity of signal transduction pathways. Moreover, cell cycle arrest, loss of proliferation, and apoptosis induction are the common phenotypes observed in response to splicing machinery loss-of-function (88, 89). CPP-SAP exhibited a modest effect on the alternative splicing of SAFA target genes in cancer cells. Since the CLIP showed that CPP-SAP did not disrupt SAFA binding to its RNA targets, this effect is not the manifestation of a co-transcriptional splicing mechanism, as has been postulated (90). Rather it suggests that splicing dysregulation by CPP-SAP may be a secondary consequence of transcriptional dysregulation. Hence, our study demonstrates an important division of labor between the DNA and RNA binding domains of SAFA in a wide range of cells.

CPP-SAP and CPP-RGG altered binding of SAFA to chromatin of only a subset of known target promoter regions and the effect was target- and cell type-specific. Further investigation is needed to identify SAP-domain-independent mechanisms for SAFA binding to the unaffected targets, and to determine how broadly DNA binding is affected by both CPP-SAP and CPP-RGG across the genome. This could reveal distinct SAFA functions (and peptide specific targets and pathways) in different types of cancer cells as also indicated by target- and cell-specific effects on splicing and levels of cell cycle gene transcripts.

Several groups have reported anti-cancer strategies with nucleic acid-based knockdowns or pharmacological inhibition of cancer drivers however, many of these proteins play critical functions in both healthy and cancer cells which like other cancer treatments, can result in life threatening side effects thereby which could limit their utility or render them no more effective than standard chemotherapy. This is the obvious advantage of identifying interventions whose effects are restricted to cancer cells (91–93) such as the CPP-SAP peptide. If the observed effects of peptide treatments are via off-target or non-specific actions, we would expect the same responses to CPP-SAP and CPP-RGG. To our knowledge we are the first to explore and develop hnRNP-derived cell-penetrating peptides as potential cancer therapies and the CPPs we describe hold promise as seen with other anti-tumor peptides such as OmoMYC and d/n/ATF5-2 (91, 93, 94). We are undertaking the next step of preclinical testing of these peptides using mouse models of BC and patient-derived tumor xenografts to assess their ability to interfere with tumor formation, progression, and metastasis *in vivo*. Our results serve as a proof-of-principle that the cell-penetrating dominant-negative domains of SAFA are efficacious, selective, and non-toxic.

## Data Availability Statement

The raw data supporting the conclusions of this article will be made available by the authors, without undue reservation.

## Author Contributions

PP conceived, designed, executed the project, and co-wrote the manuscript. AM provided the valuable support and co-wrote the manuscript. Both authors contributed to the article and approved the submitted version.

## Conflict of Interest

The authors declare that the research was conducted in the absence of any commercial or financial relationships that could be construed as a potential conflict of interest.
