# Supplementary figures and images for: Novel Cell-Penetrating Peptides Derived From Scaffold-Attachment- Factor A Inhibits Cancer Cell Proliferation and Survival
Source: Front Oncol. 2021 Mar 30;11:621825. doi: 10.3389/fonc.2021.621825 (PMC8042391; doi:10.3389/fonc.2021.621825)

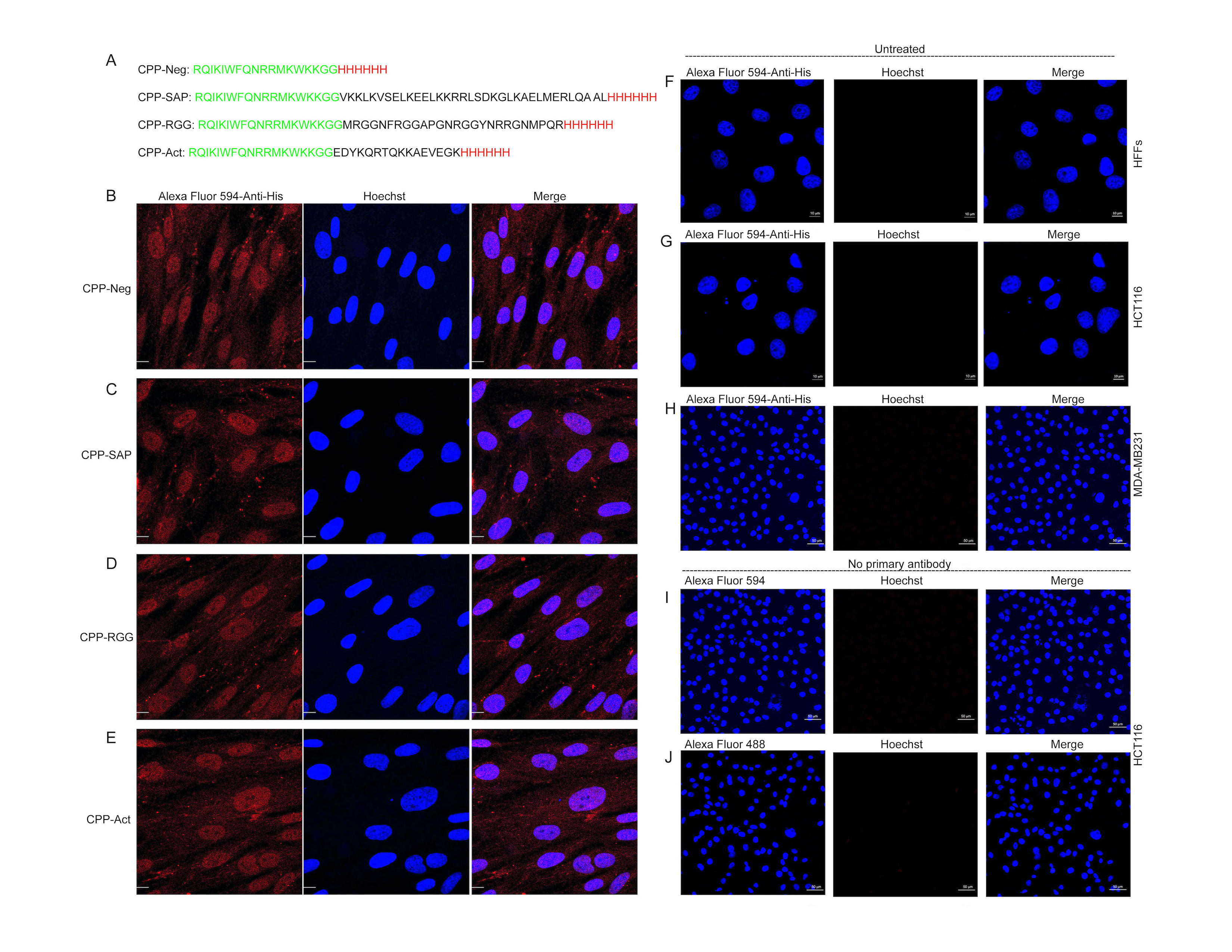

Supplement: Supplementary file 1 [file Data_Sheet_1.zip › 1/S. Fig. 1.1.jpg]

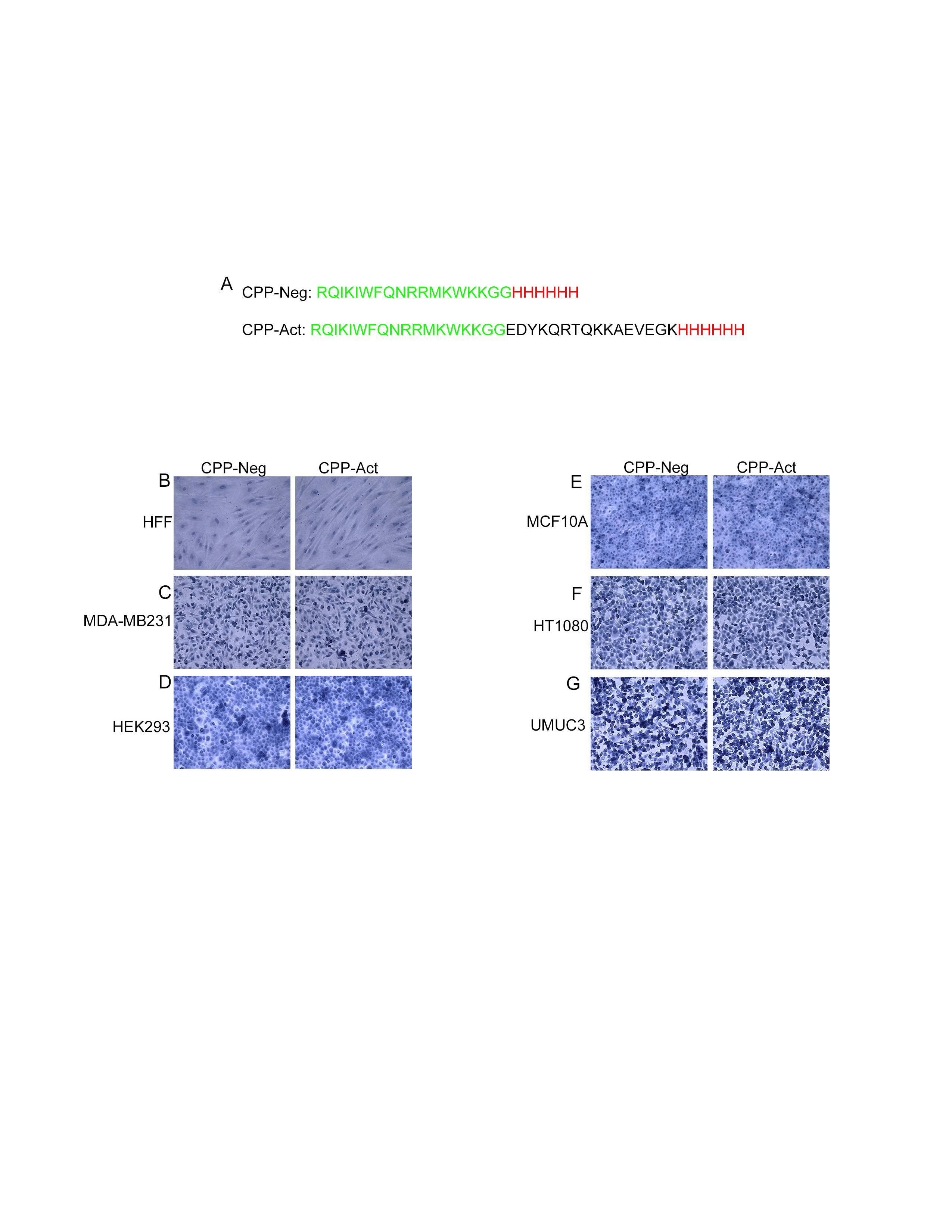

Supplement: Supplementary file 1 [file Data_Sheet_1.zip › 1/S. Fig. 1.2.jpg]

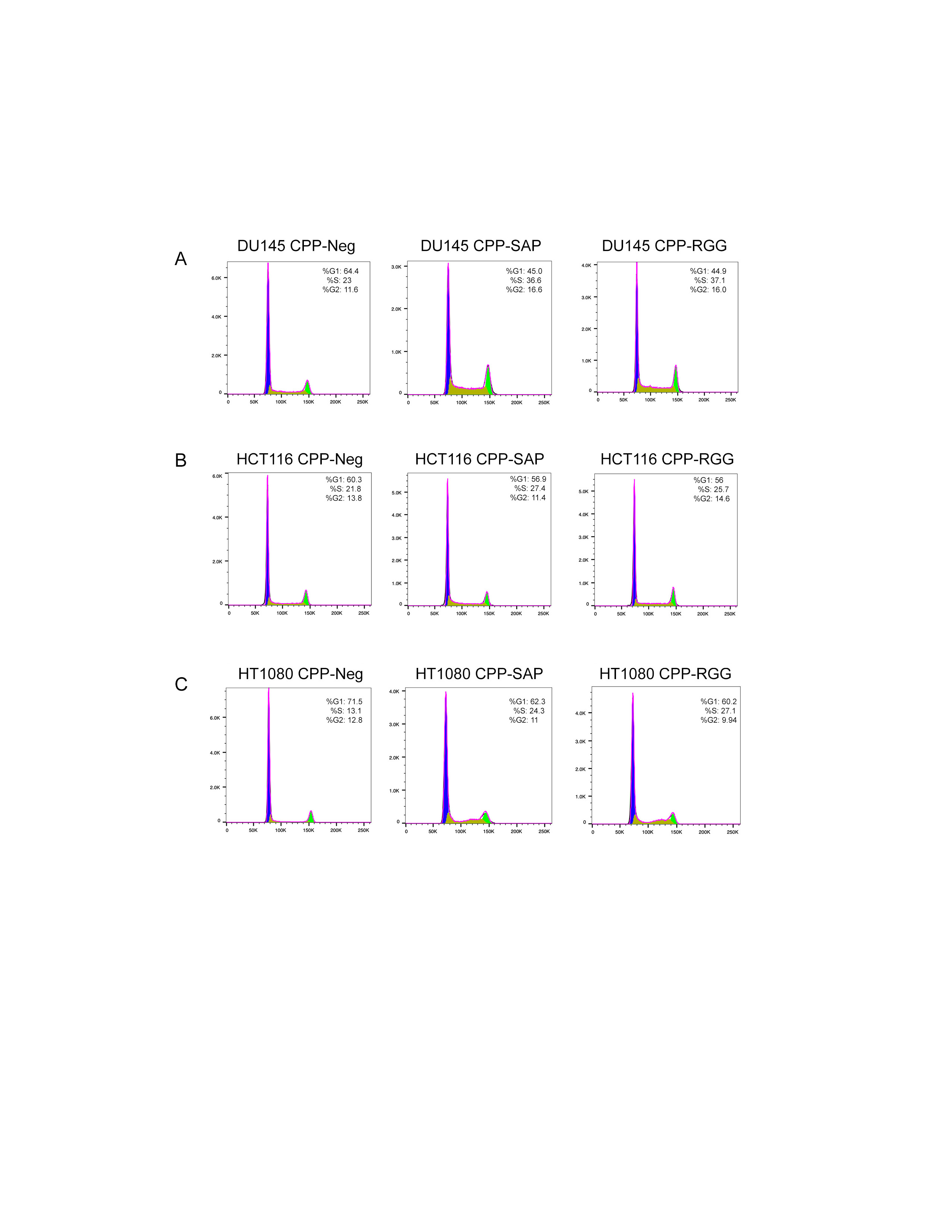

Supplement: Supplementary file 2 [file Data_Sheet_2.zip › 2/S. Fig. 2.4.jpg]

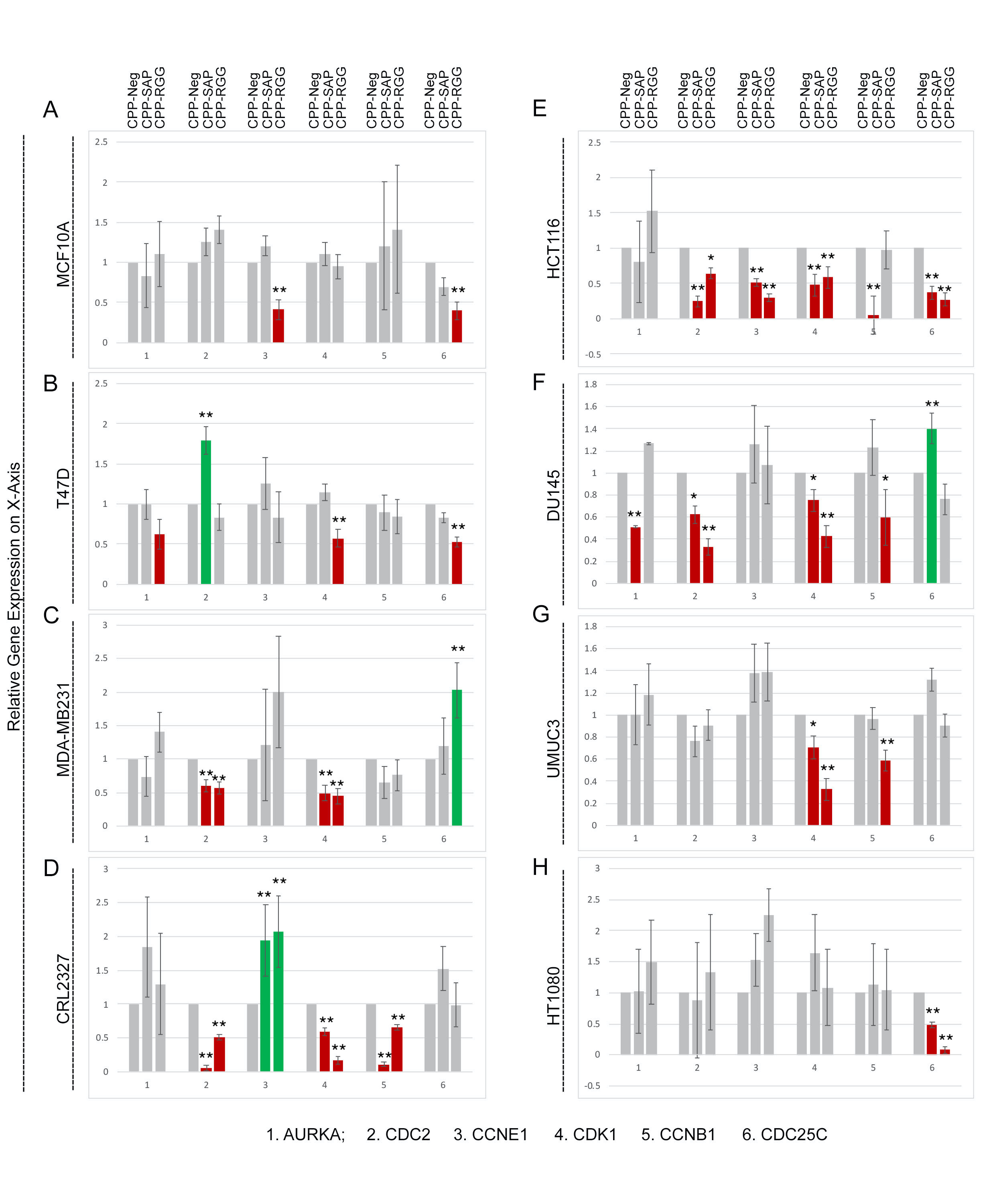

Supplement: Supplementary file 2 [file Data_Sheet_2.zip › 2/S. Fig. 2.5.jpg]

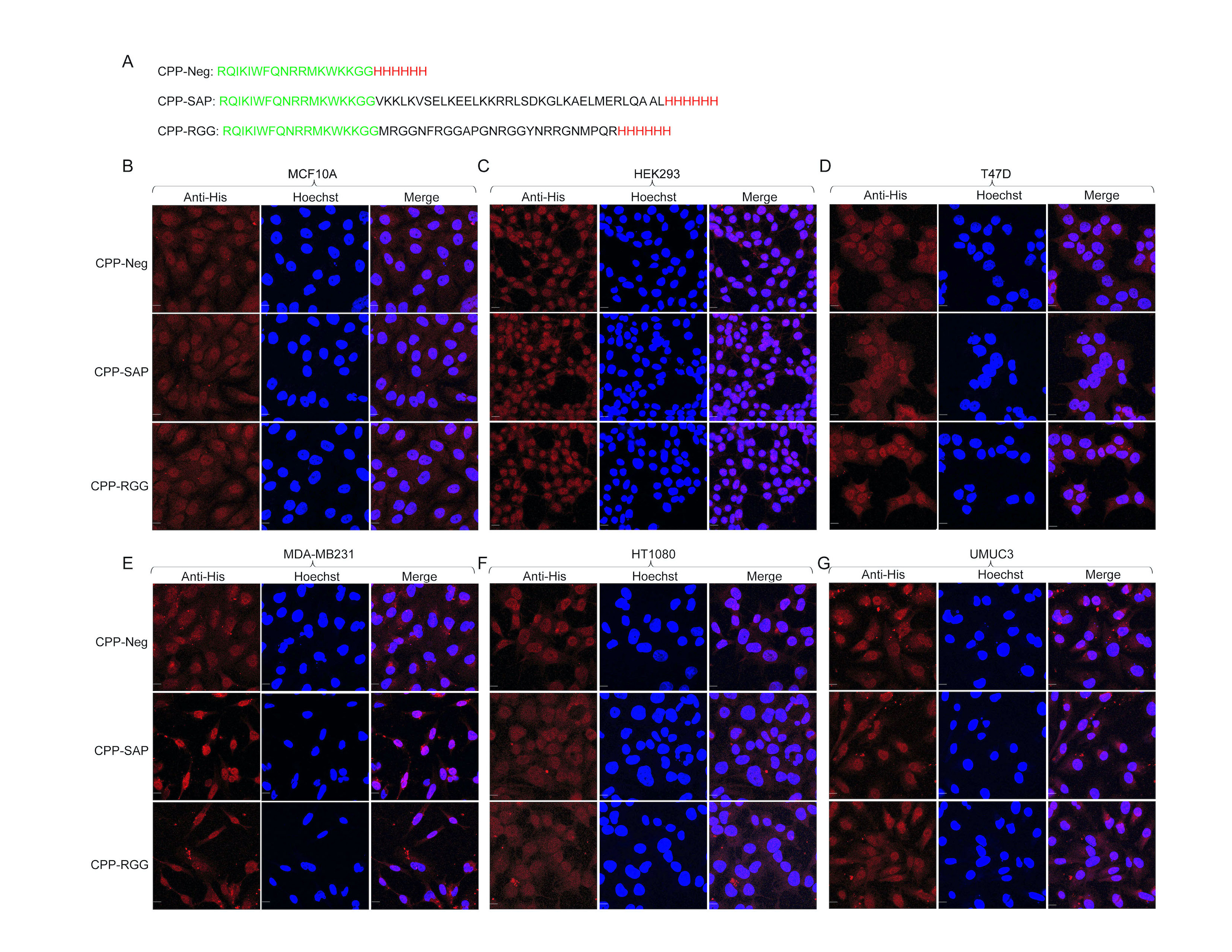

Supplement: Supplementary file 2 [file Data_Sheet_2.zip › 2/S. Fig. 2.1.jpg]

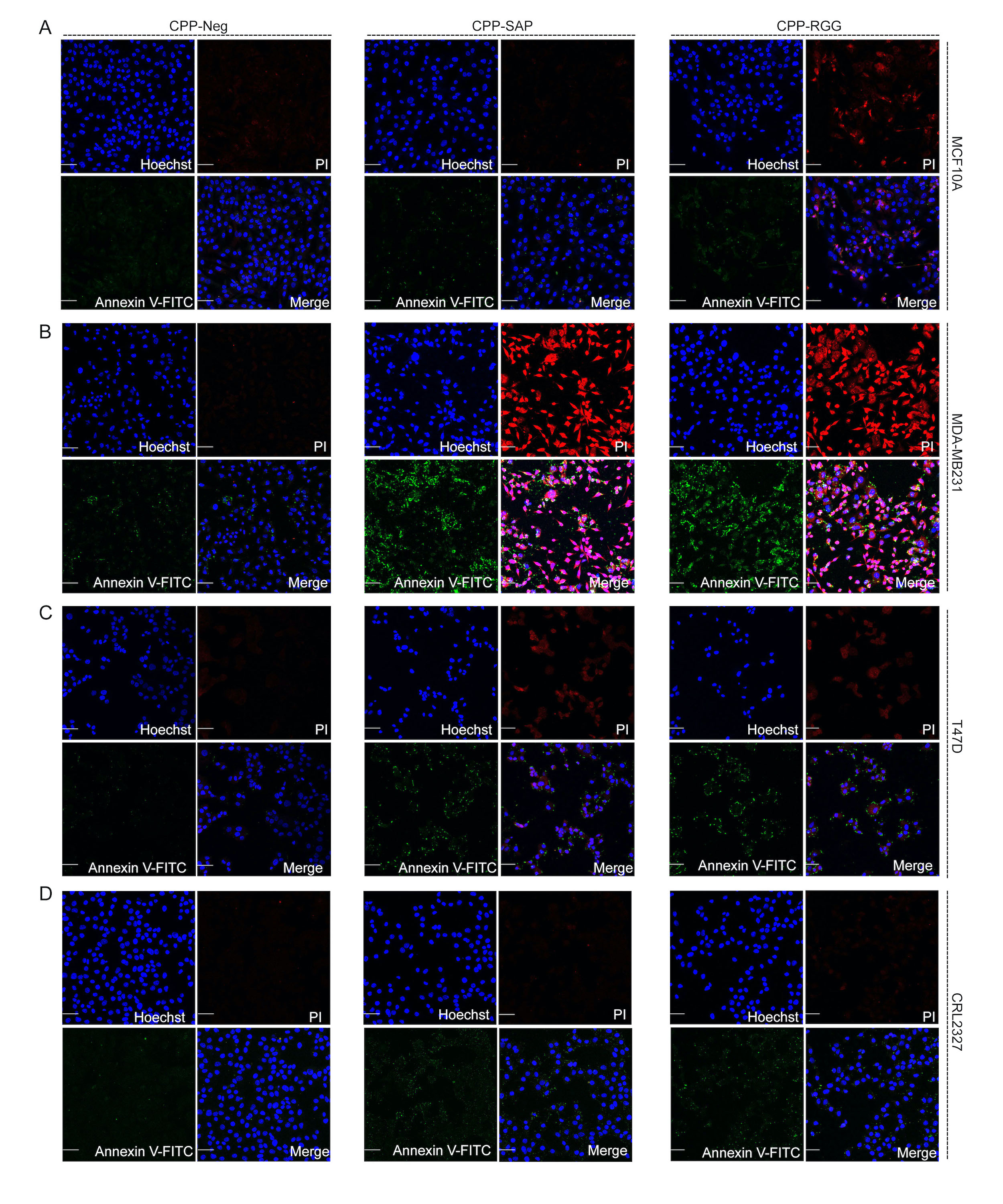

Supplement: Supplementary file 2 [file Data_Sheet_2.zip › 2/S. Fig. 2.2.jpg]

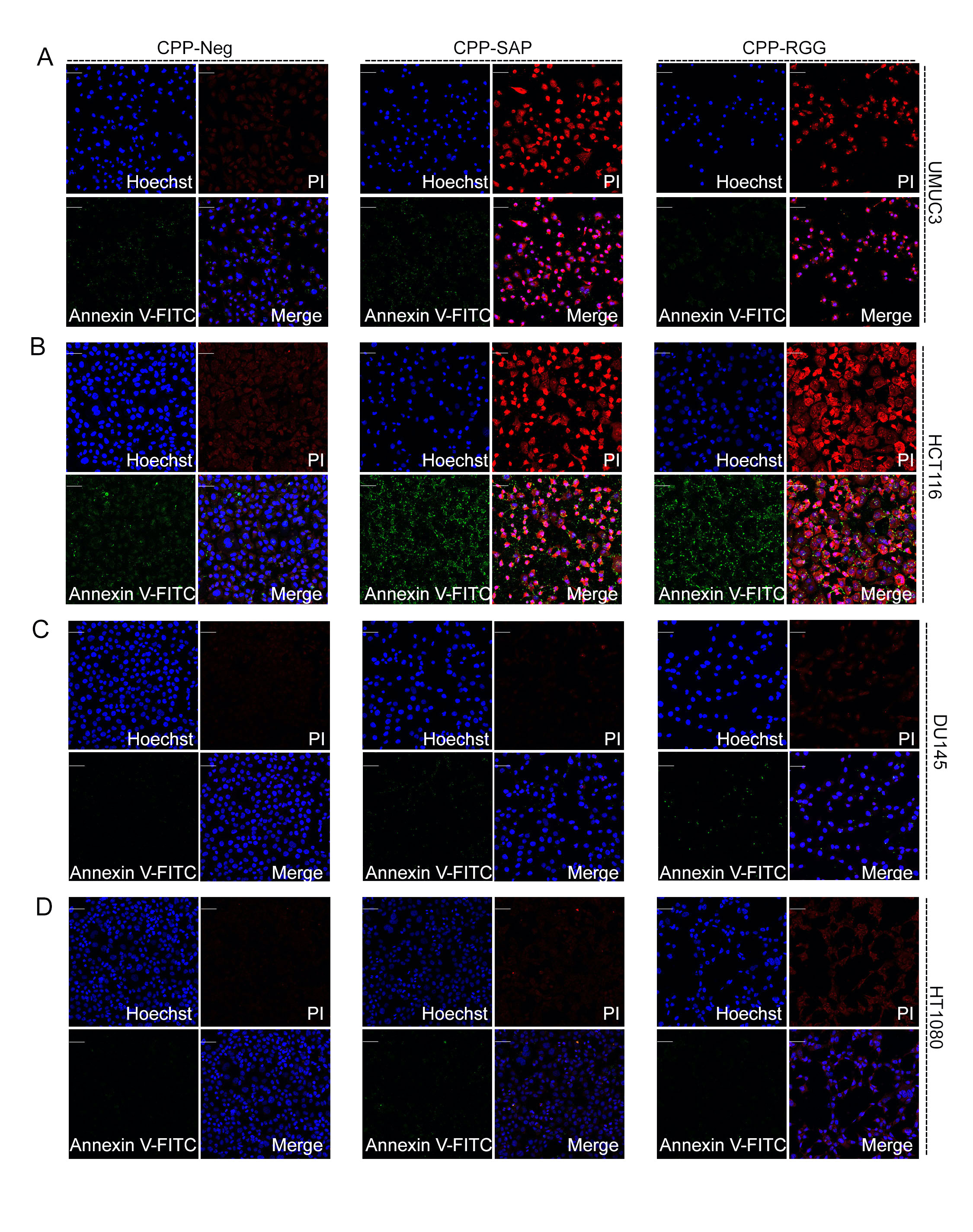

Supplement: Supplementary file 2 [file Data_Sheet_2.zip › 2/S. Fig. 2.3.jpg]

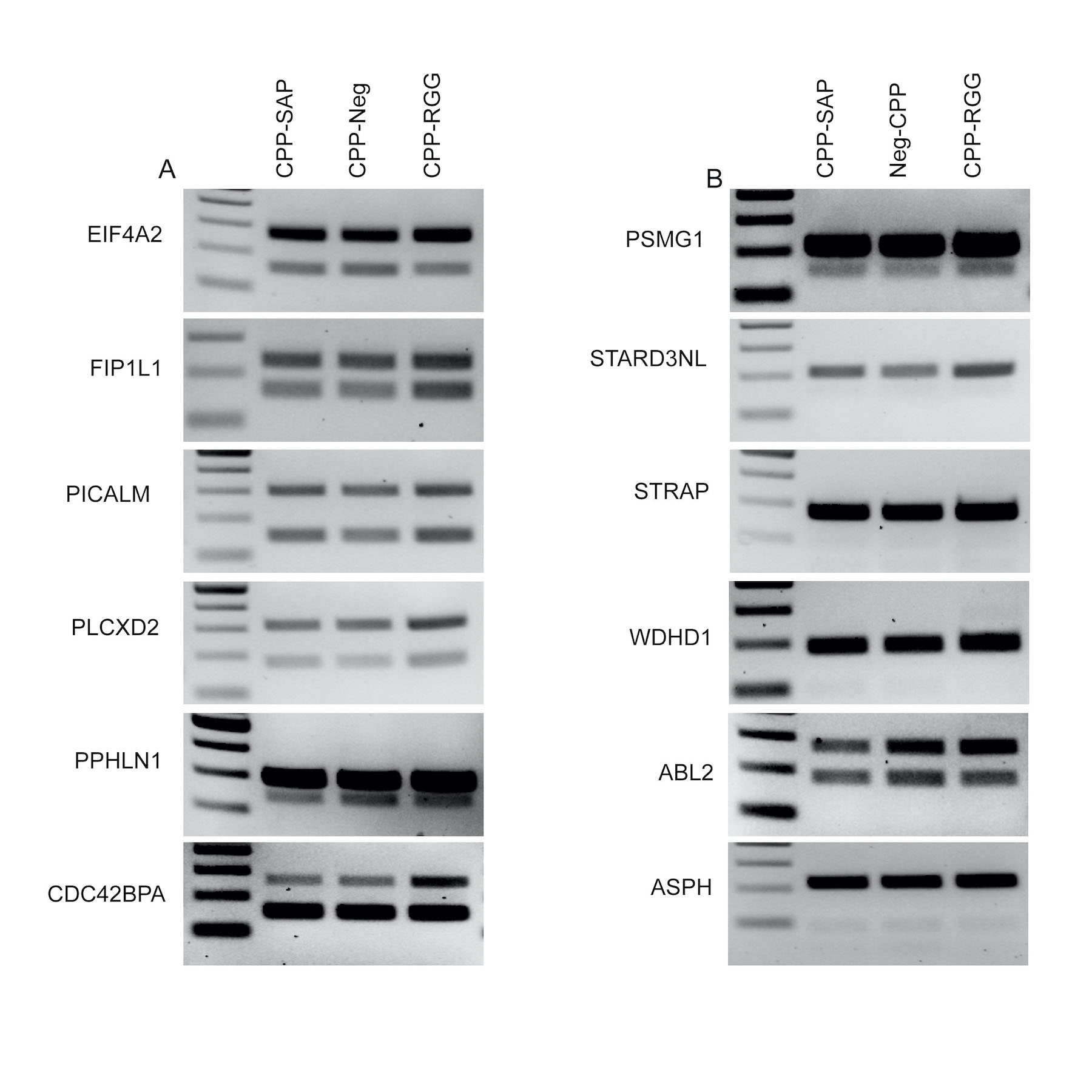

Supplement: Supplementary file 3 [file Data_Sheet_3.zip › 3/S. Fig. 3.2.jpg]

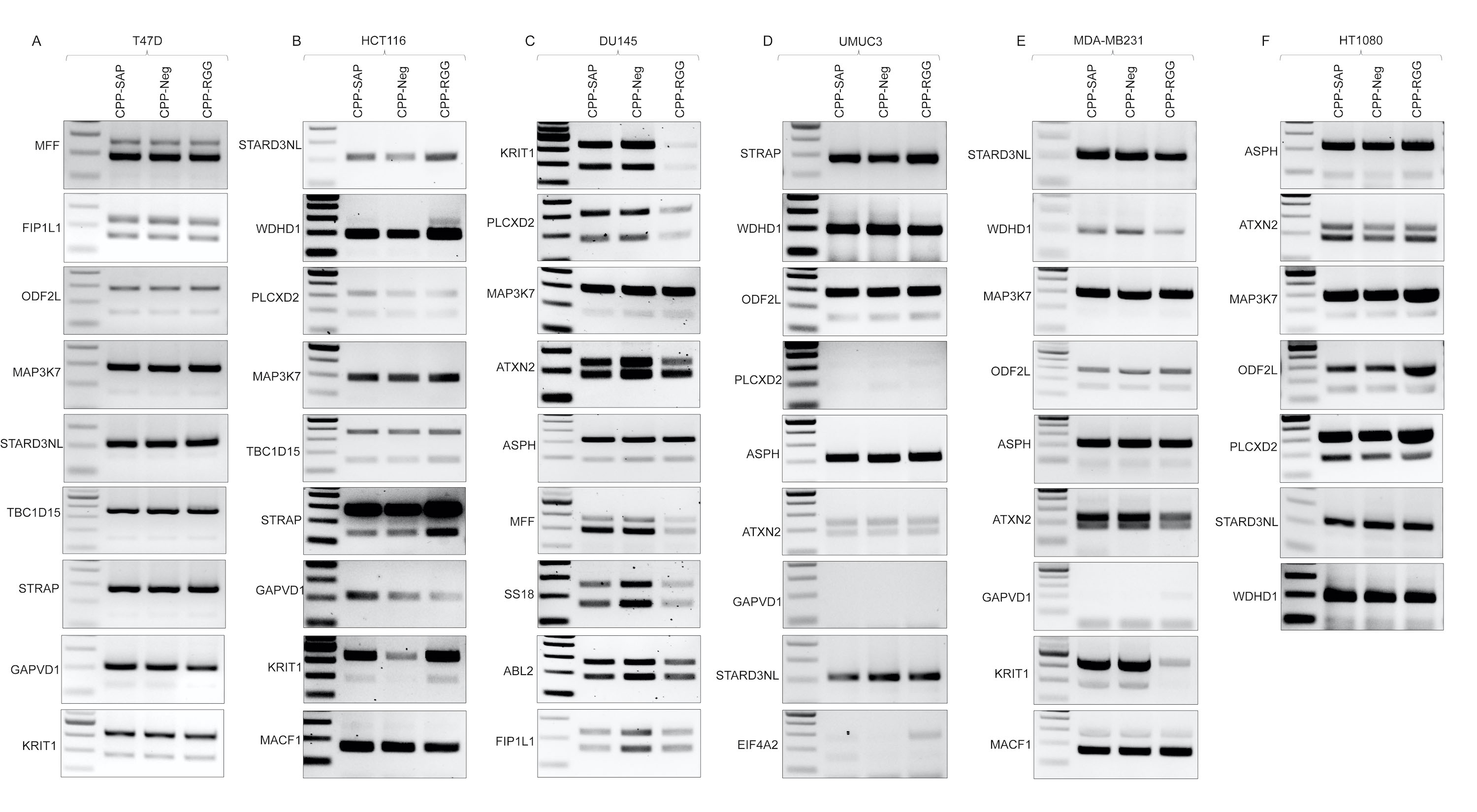

Supplement: Supplementary file 3 [file Data_Sheet_3.zip › 3/S. Fig. 3.1.jpg]

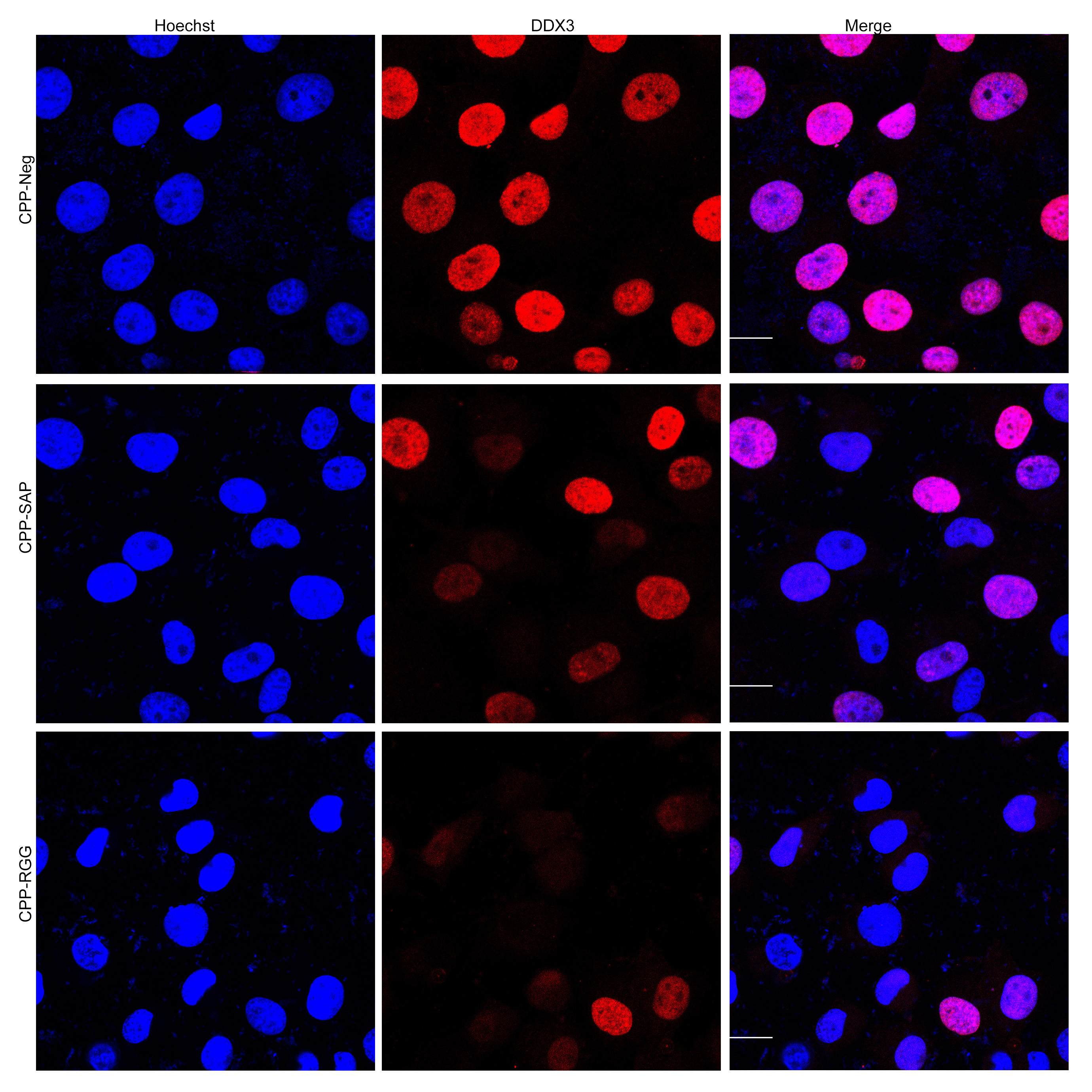

Supplement: Supplementary file 4 [file Data_Sheet_4.zip › Data Sheet 4/S. Fig. 4.2.2.jpg]

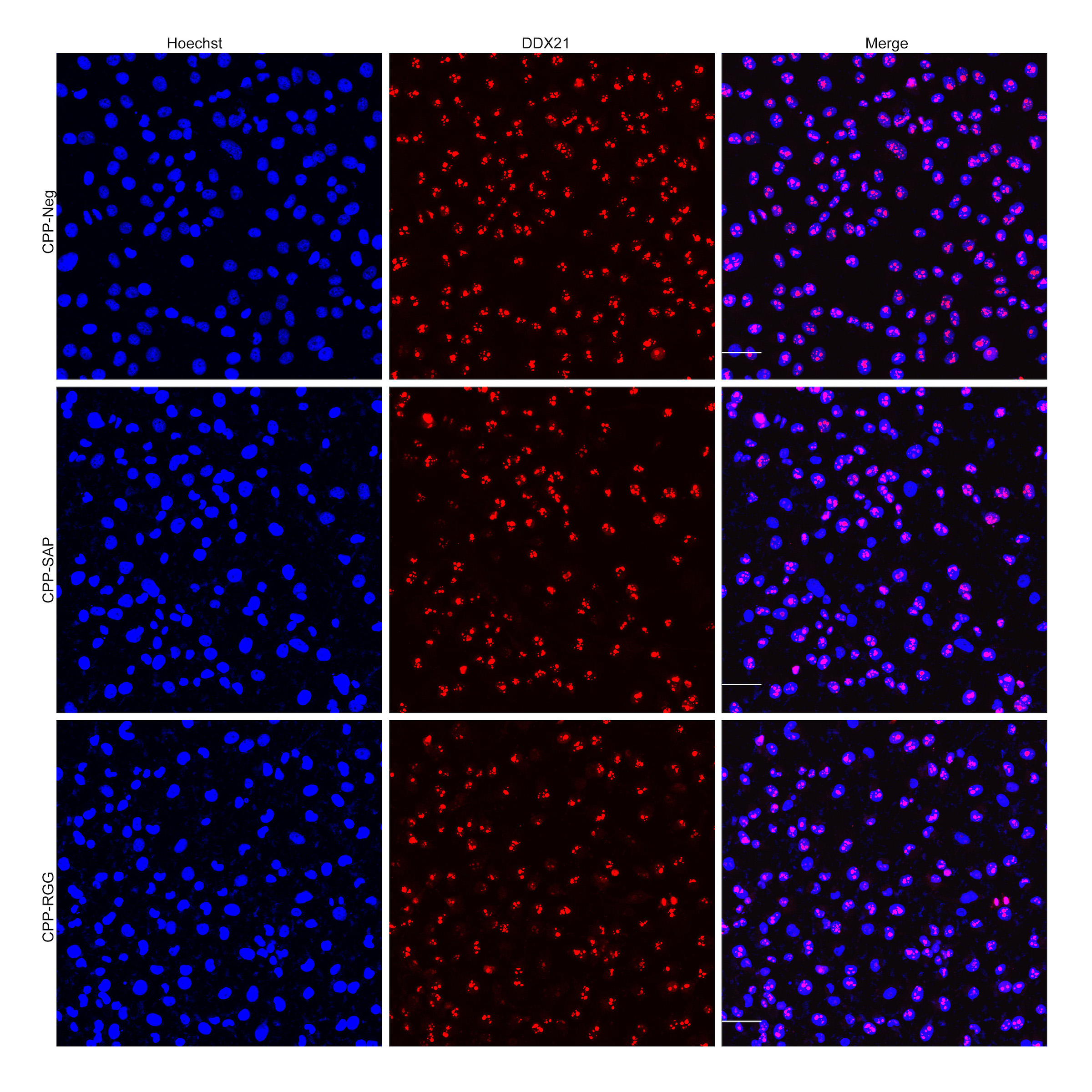

Supplement: Supplementary file 4 [file Data_Sheet_4.zip › Data Sheet 4/S. Fig. 4.2.3.jpg]

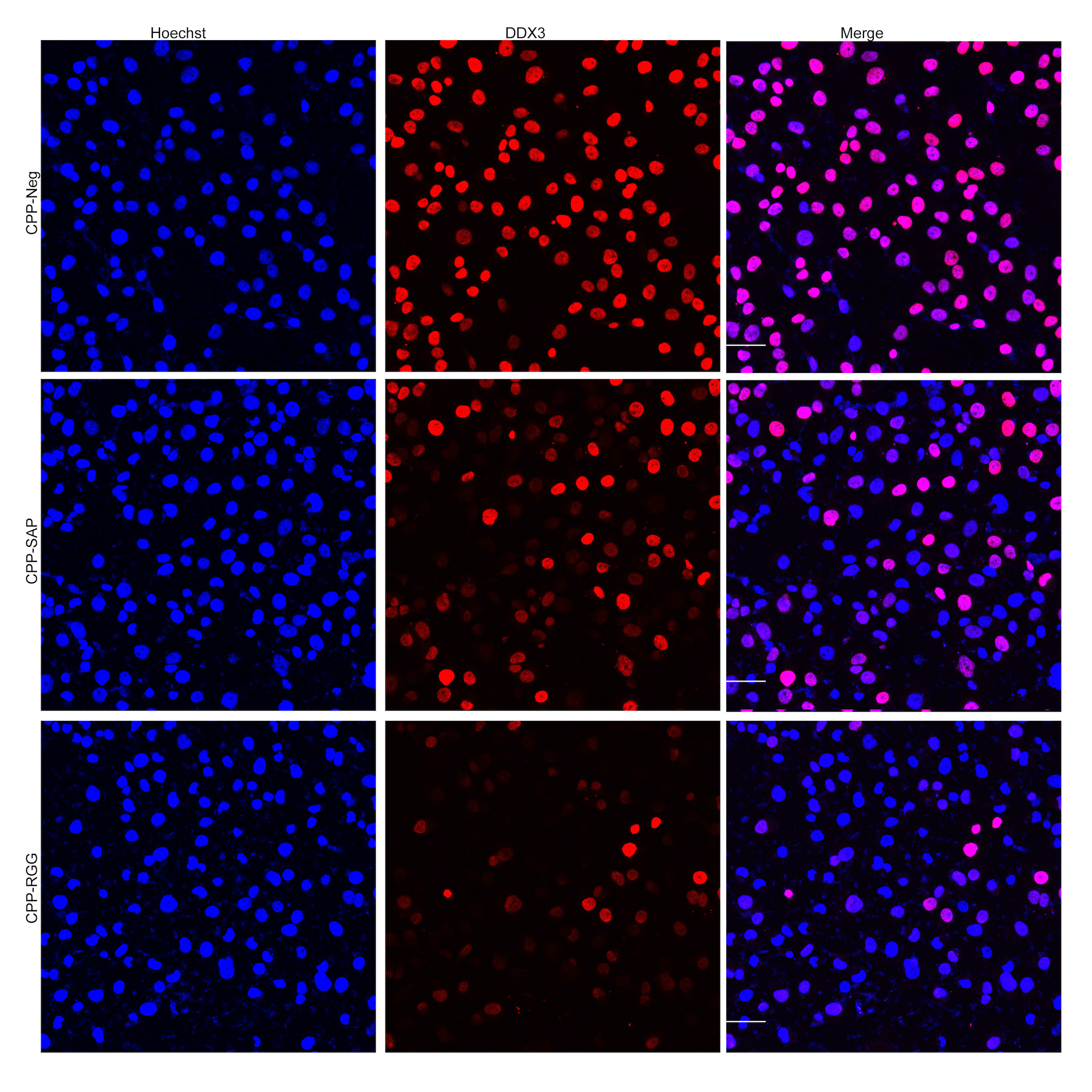

Supplement: Supplementary file 4 [file Data_Sheet_4.zip › Data Sheet 4/S. Fig. 4.2.1.jpg]

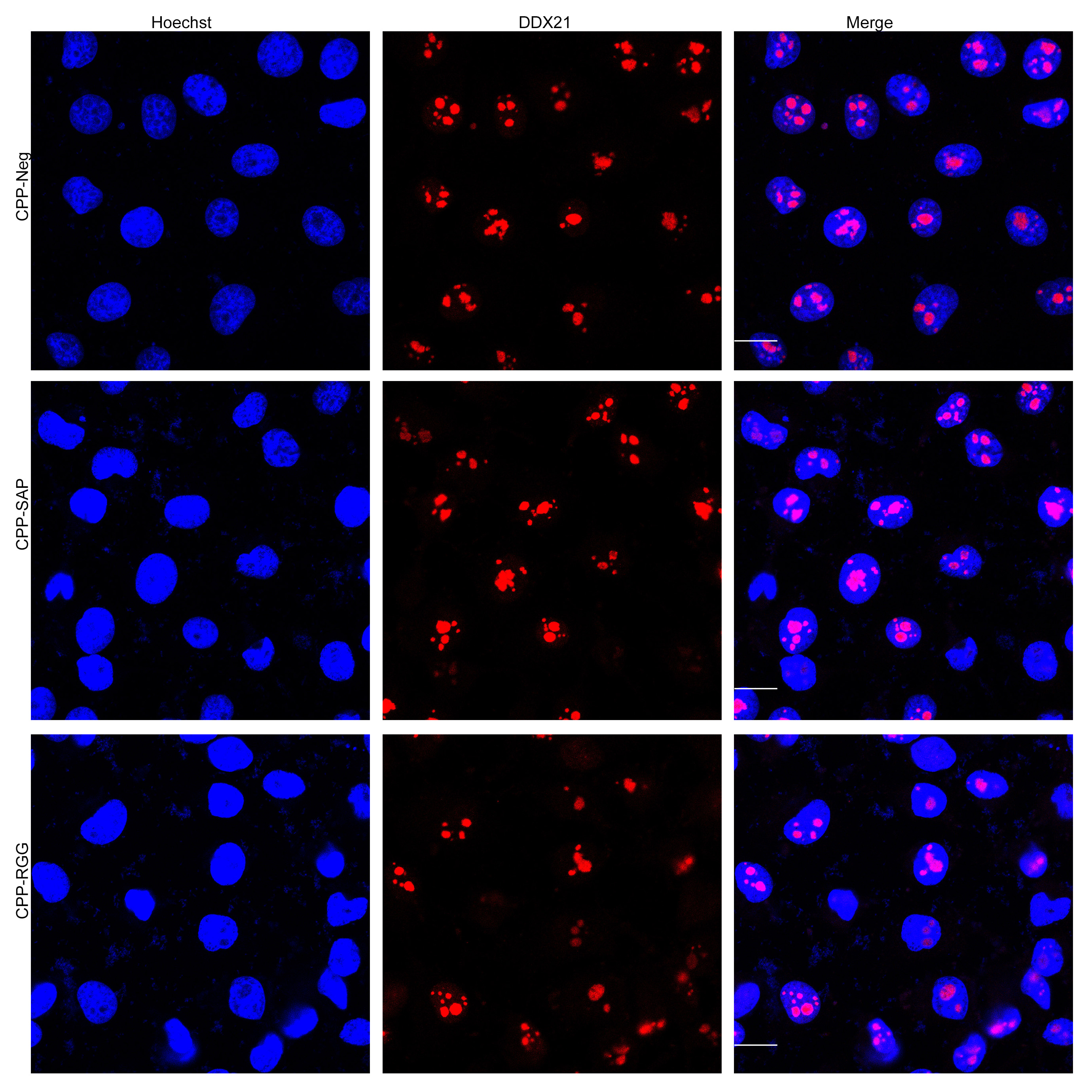

Supplement: Supplementary file 4 [file Data_Sheet_4.zip › Data Sheet 4/S. Fig. 4.2.4.jpg]

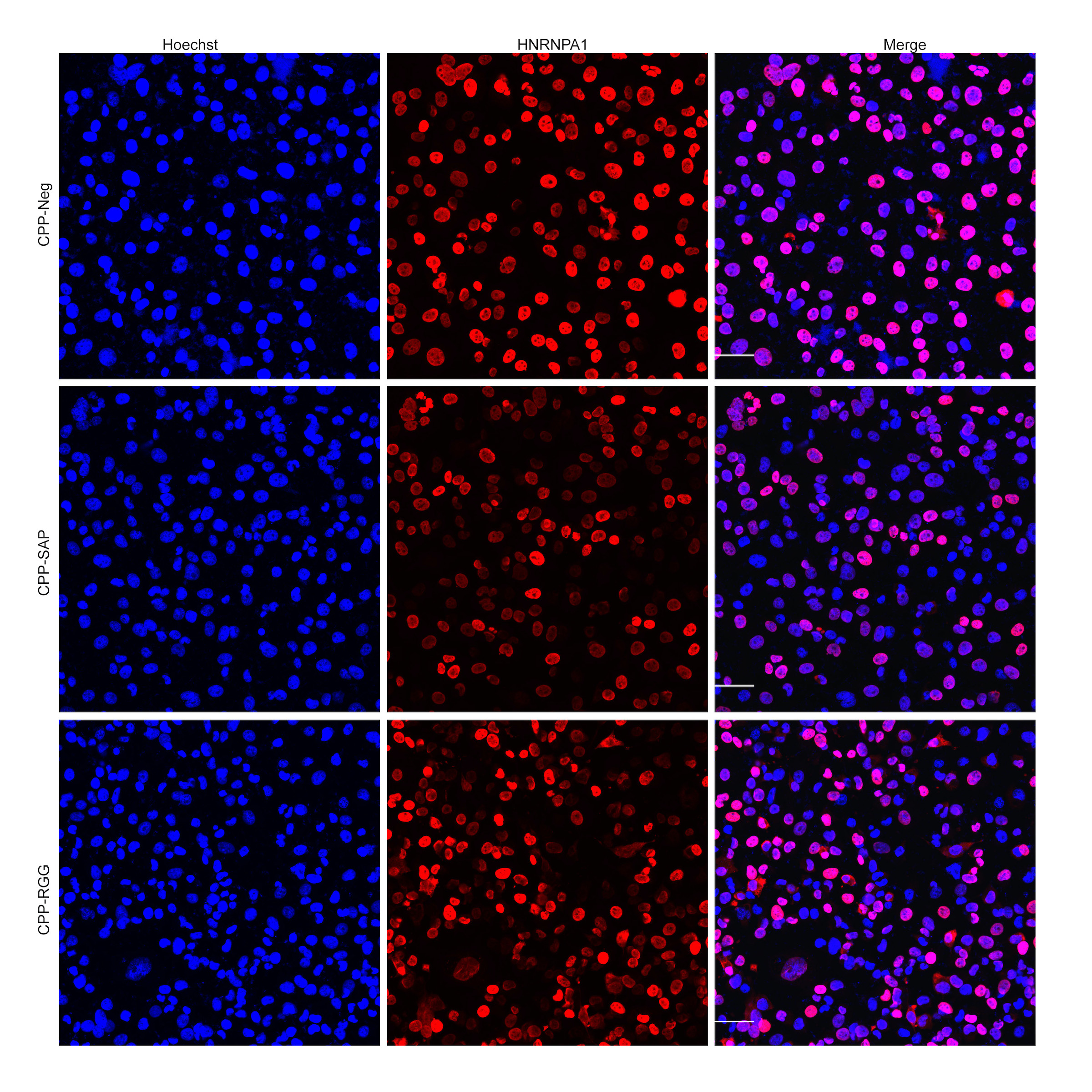

Supplement: Supplementary file 4 [file Data_Sheet_4.zip › Data Sheet 4/S. Fig. 4.2.5.jpg]

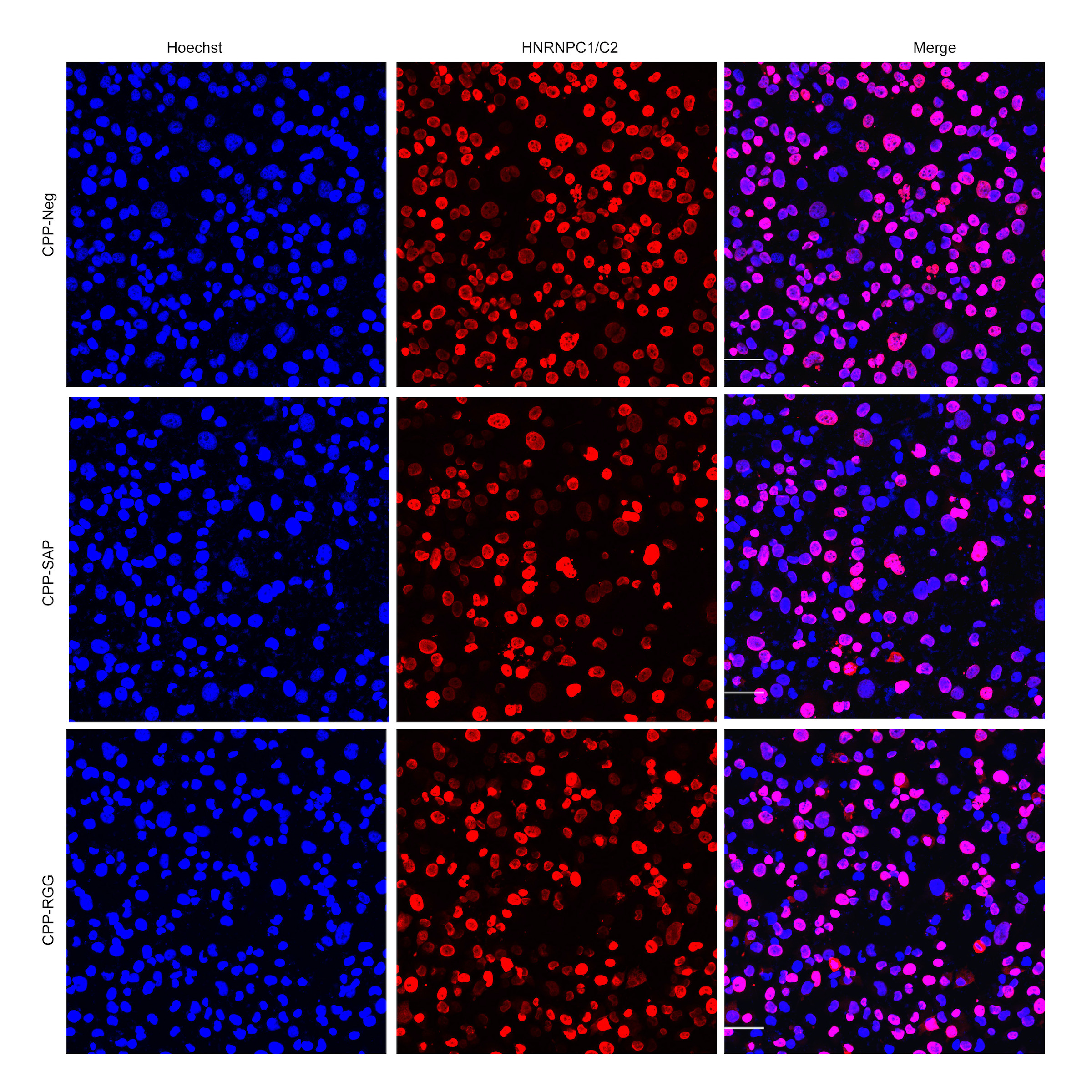

Supplement: Supplementary file 4 [file Data_Sheet_4.zip › Data Sheet 4/S. Fig. 4.2.7.jpg]

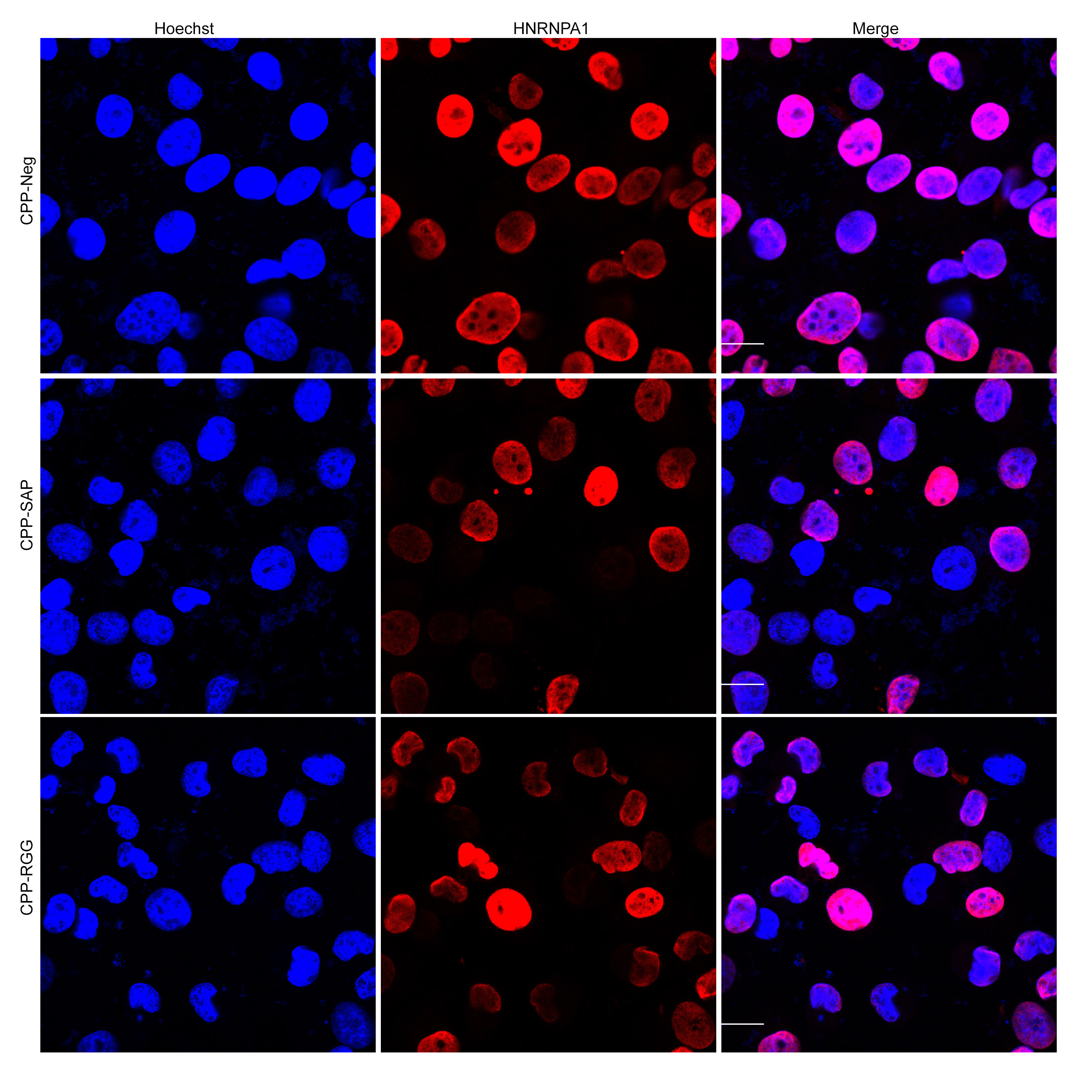

Supplement: Supplementary file 4 [file Data_Sheet_4.zip › Data Sheet 4/S. Fig. 4.2.6.jpg]

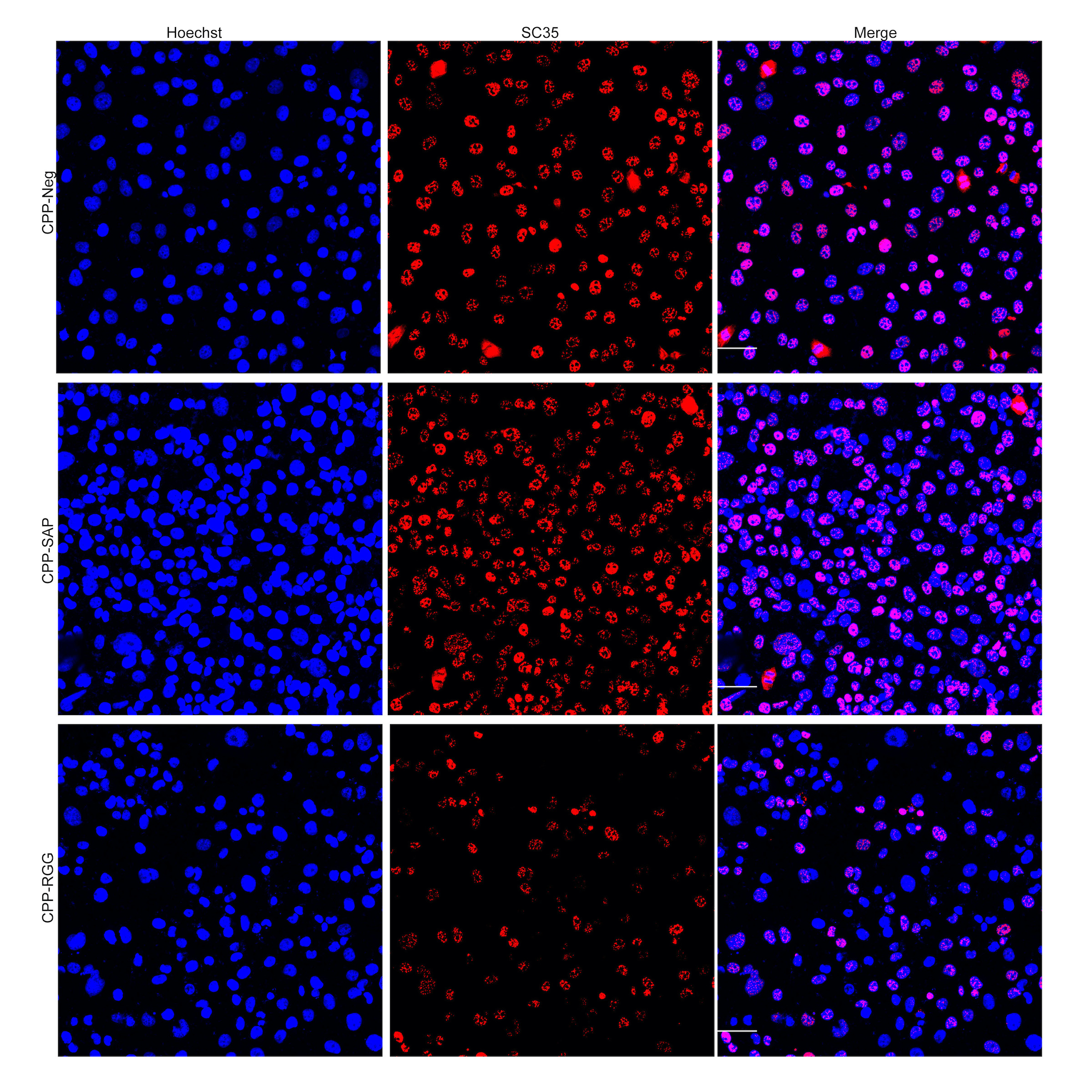

Supplement: Supplementary file 4 [file Data_Sheet_4.zip › Data Sheet 4/S. Fig. 4.2.13.jpg]

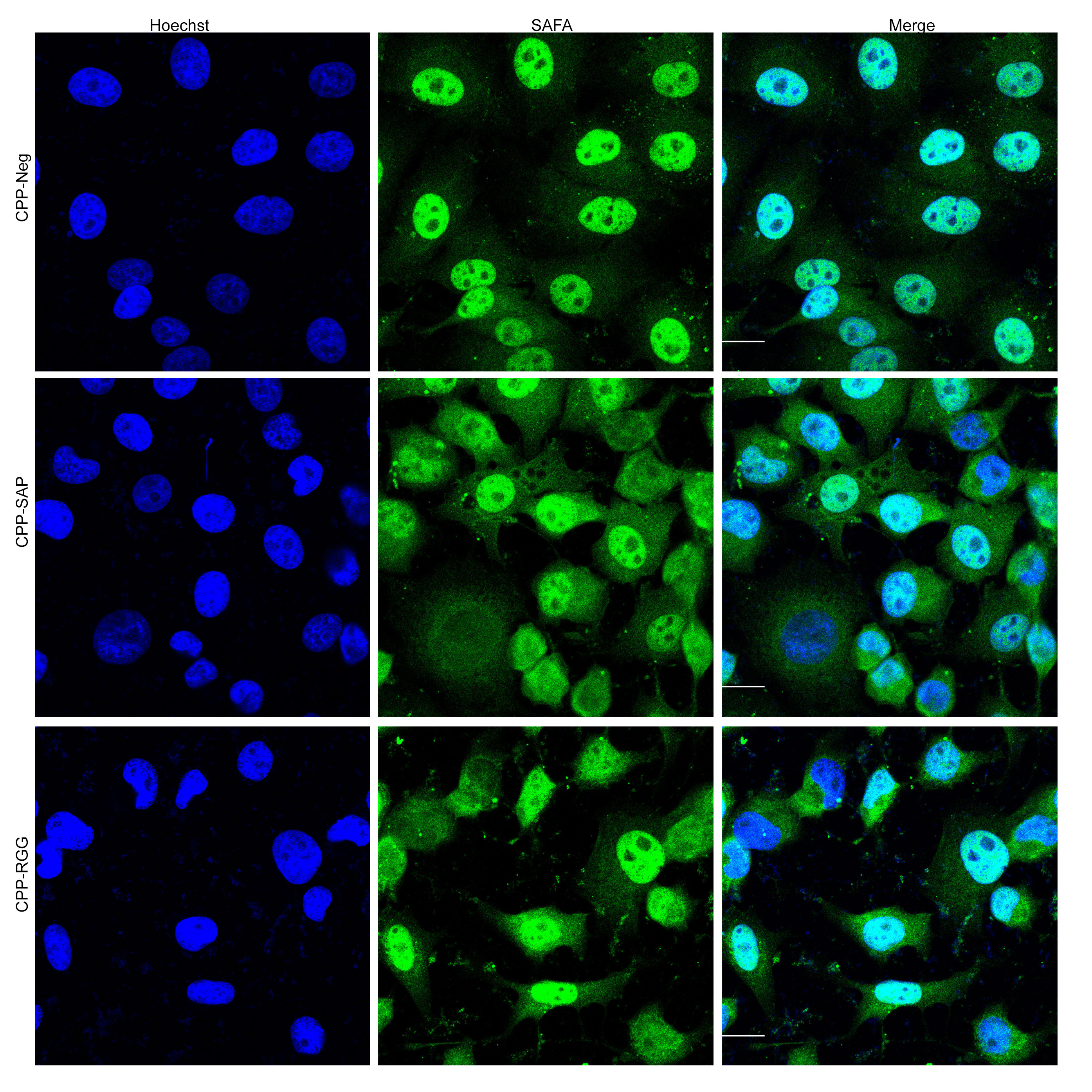

Supplement: Supplementary file 4 [file Data_Sheet_4.zip › Data Sheet 4/S. Fig. 4.2.12.jpg]

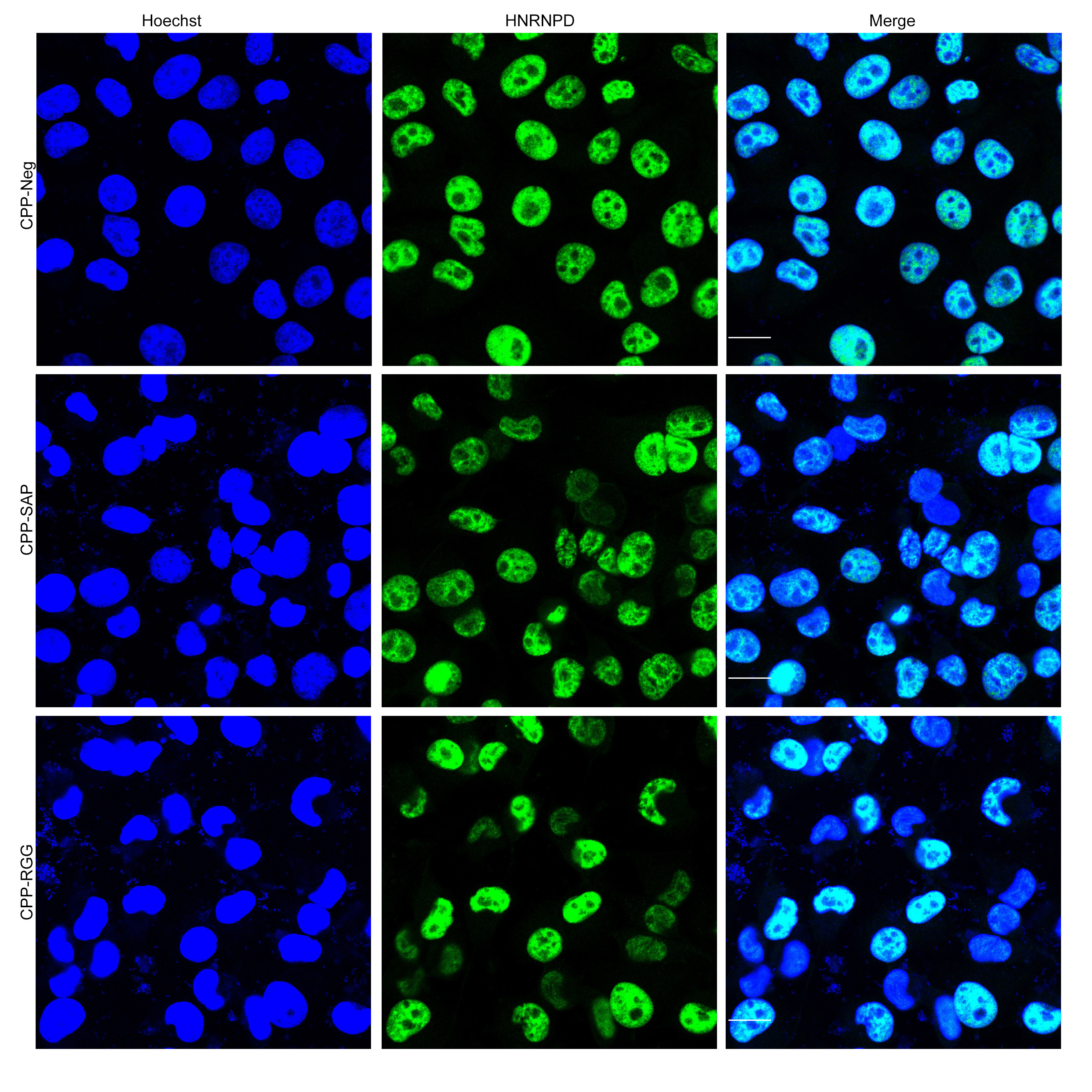

Supplement: Supplementary file 4 [file Data_Sheet_4.zip › Data Sheet 4/S. Fig. 4.2.10.jpg]

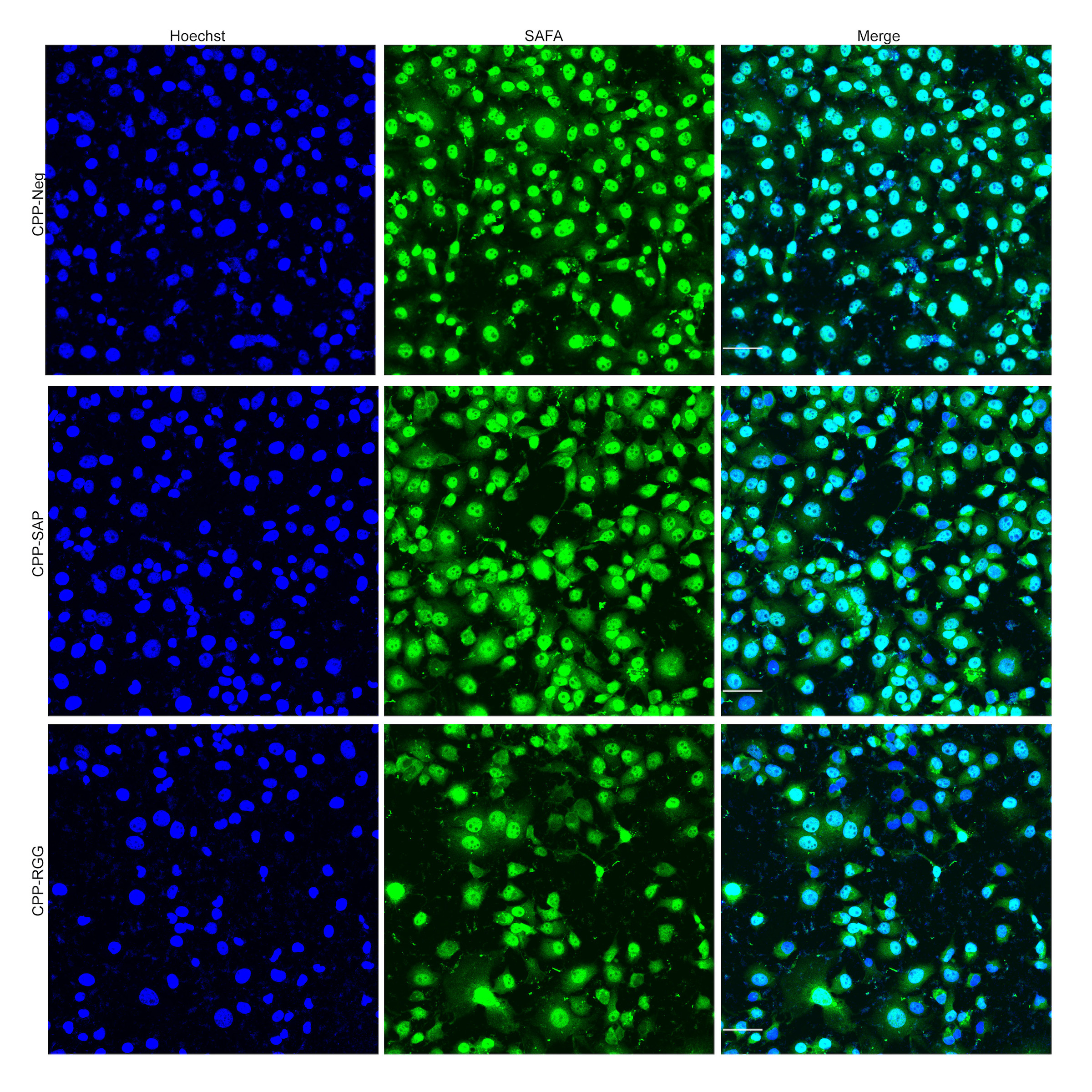

Supplement: Supplementary file 4 [file Data_Sheet_4.zip › Data Sheet 4/S. Fig. 4.2.11.jpg]

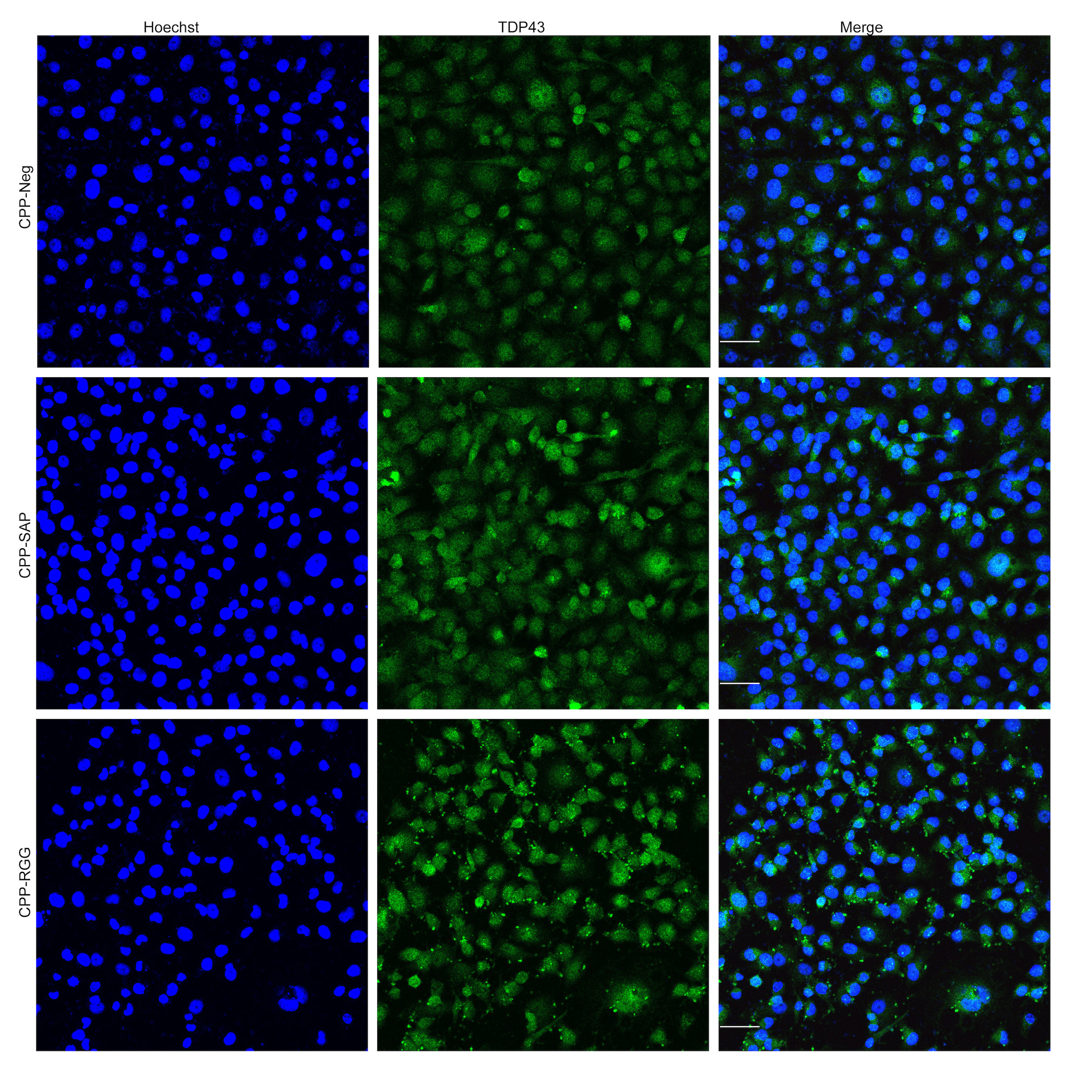

Supplement: Supplementary file 4 [file Data_Sheet_4.zip › Data Sheet 4/S. Fig. 4.2.15.jpg]

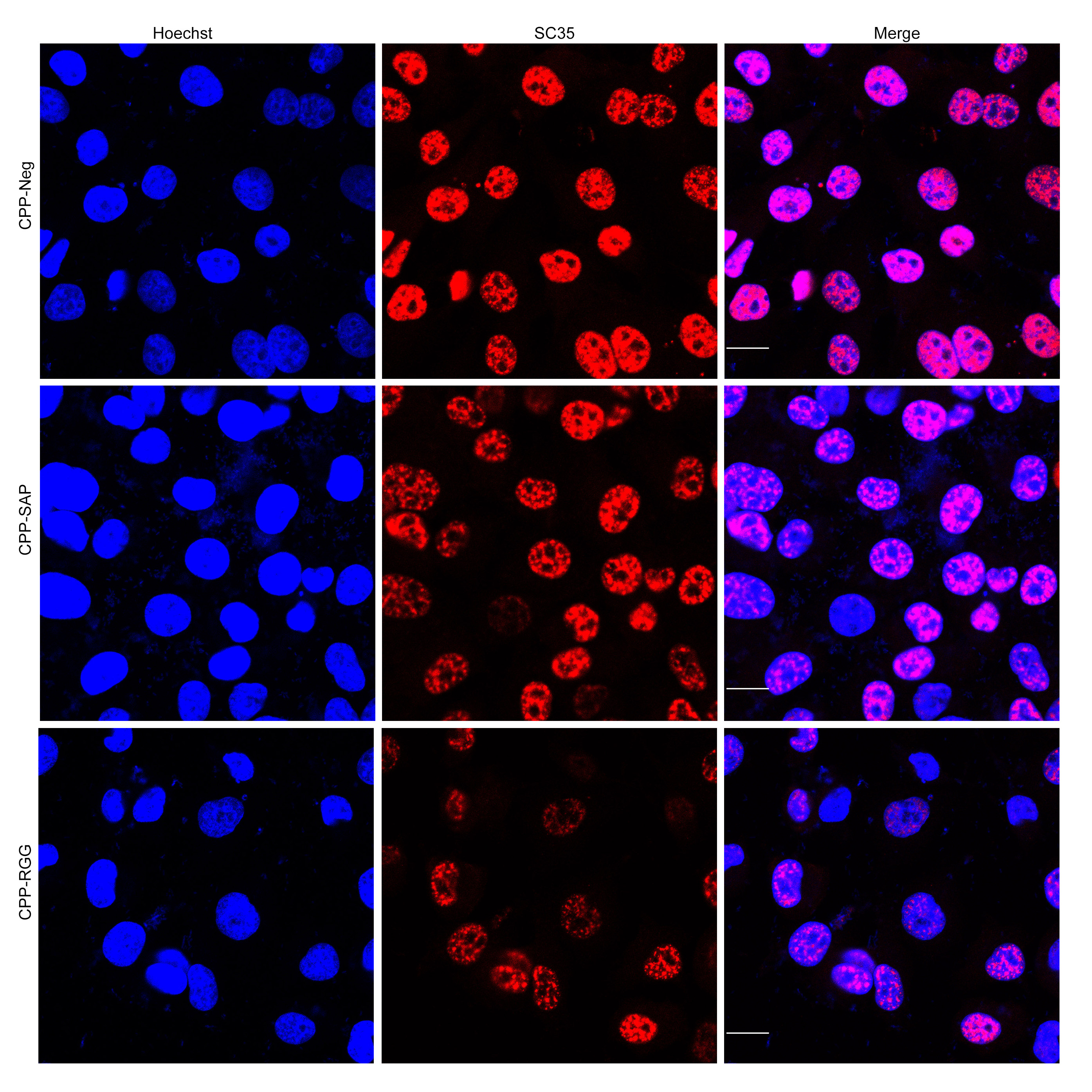

Supplement: Supplementary file 4 [file Data_Sheet_4.zip › Data Sheet 4/S. Fig. 4.2.14.jpg]

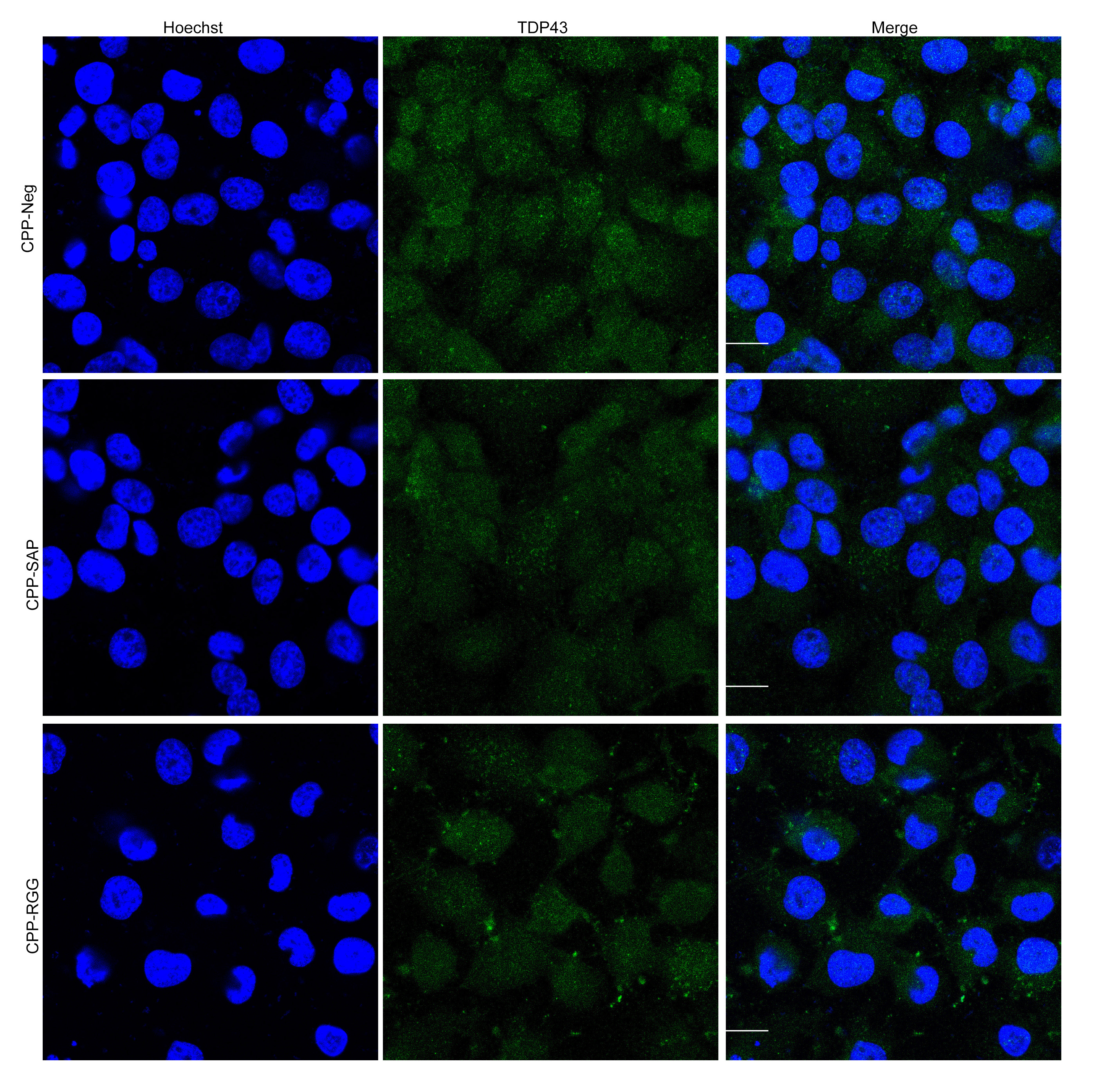

Supplement: Supplementary file 4 [file Data_Sheet_4.zip › Data Sheet 4/S. Fig. 4.2.16.jpg]

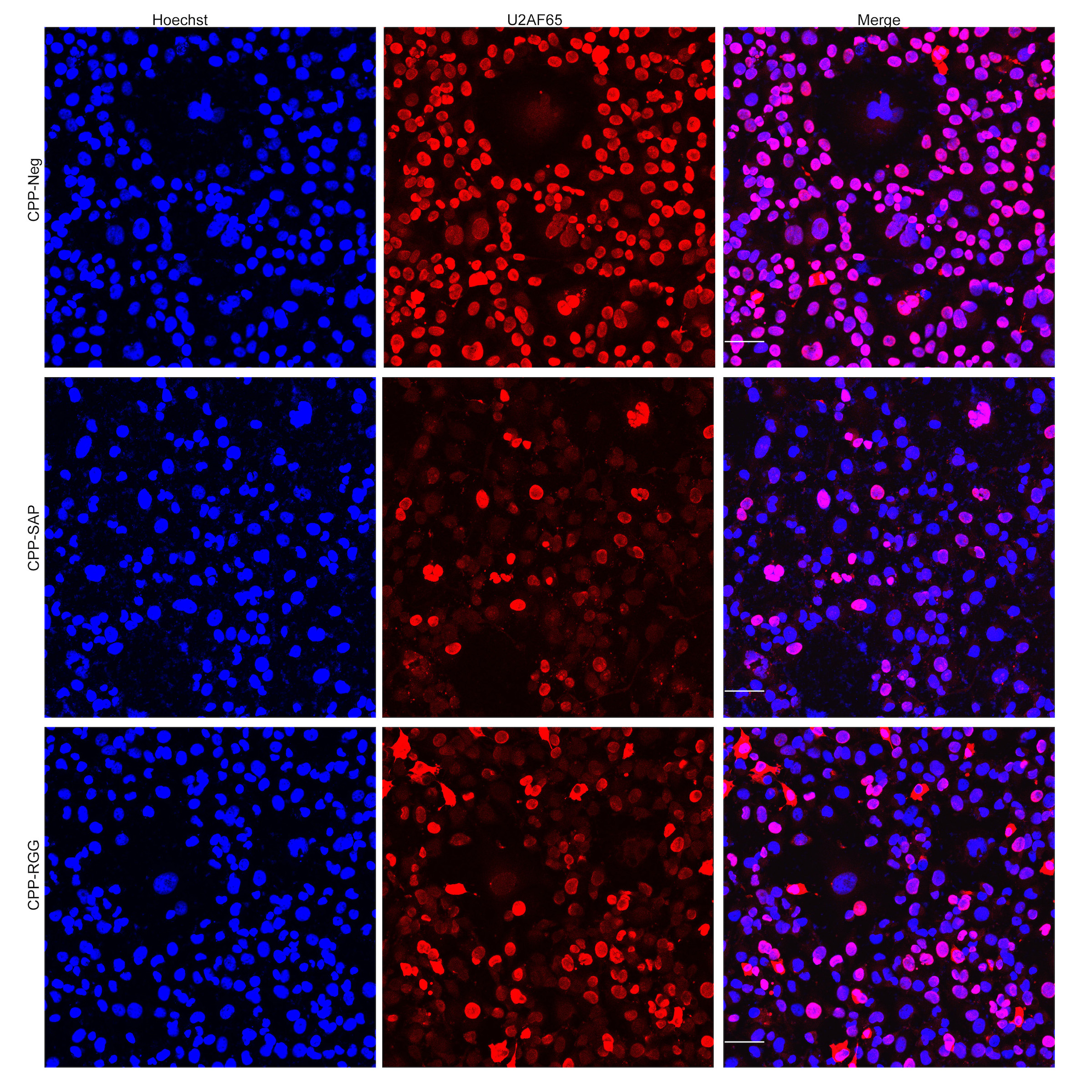

Supplement: Supplementary file 4 [file Data_Sheet_4.zip › Data Sheet 4/S. Fig. 4.2.17.jpg]

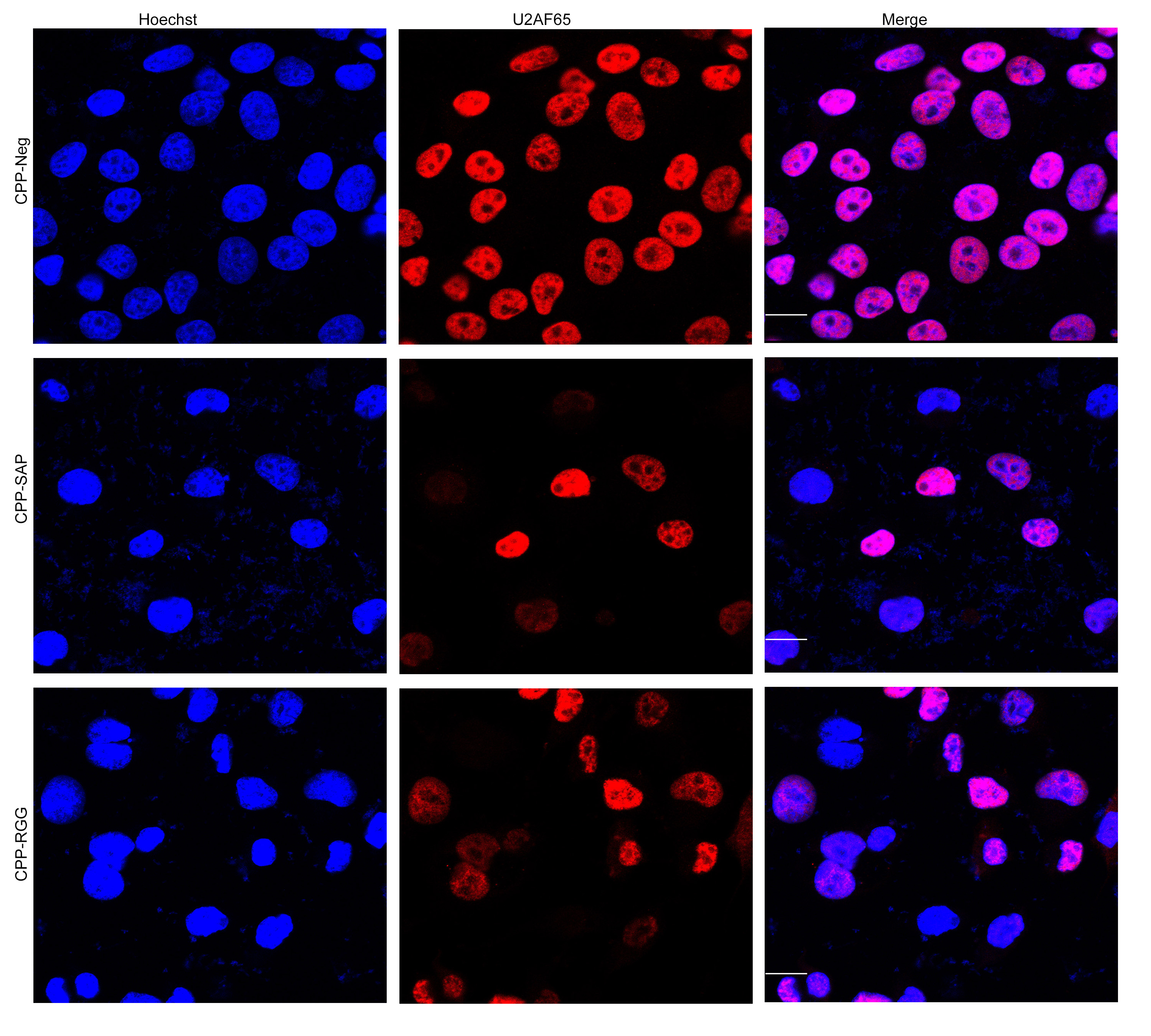

Supplement: Supplementary file 4 [file Data_Sheet_4.zip › Data Sheet 4/S. Fig. 4.2.18.jpg]

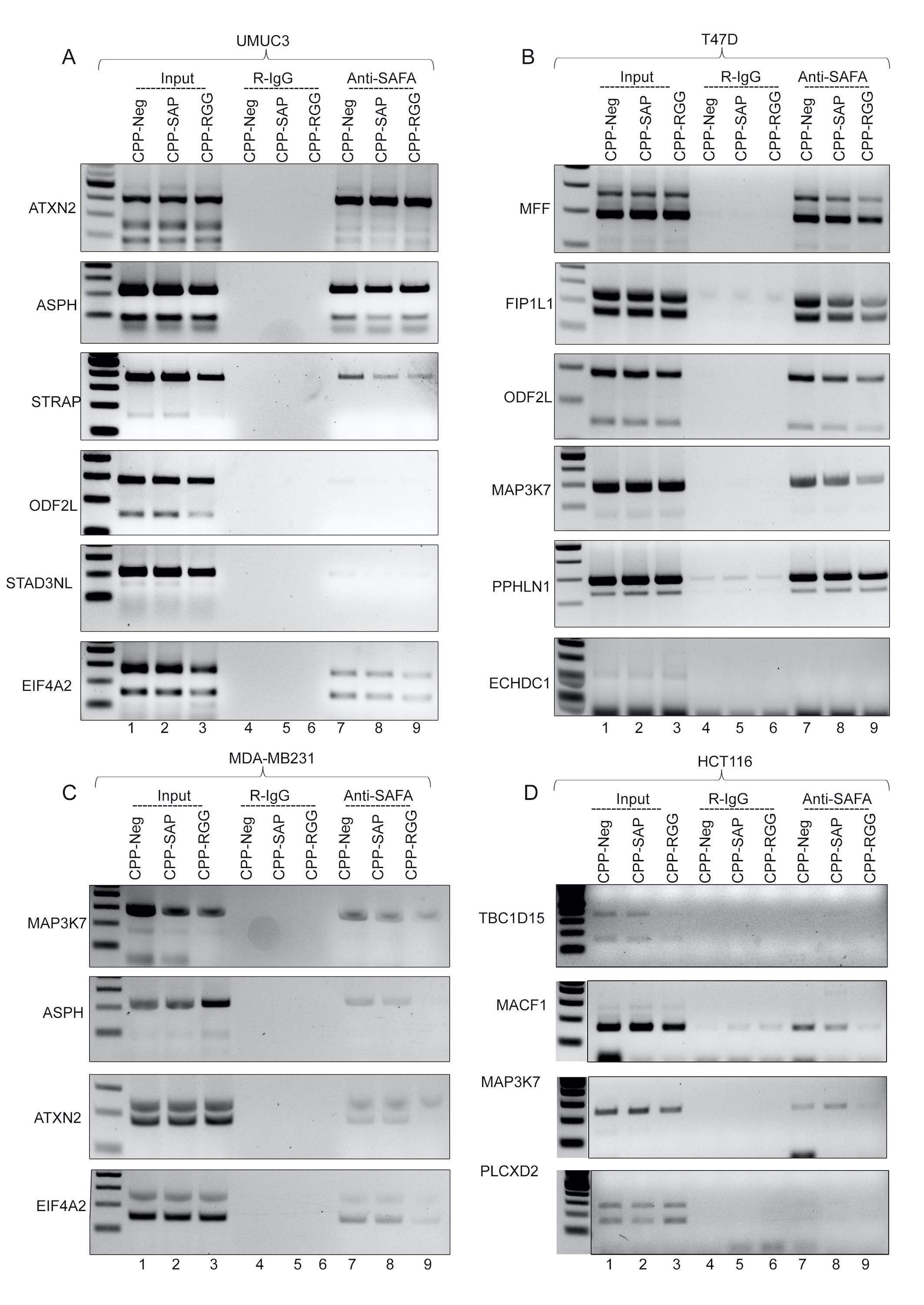

Supplement: Supplementary file 4 [file Data_Sheet_4.zip › Data Sheet 4/S. Fig. 4.1.jpg]

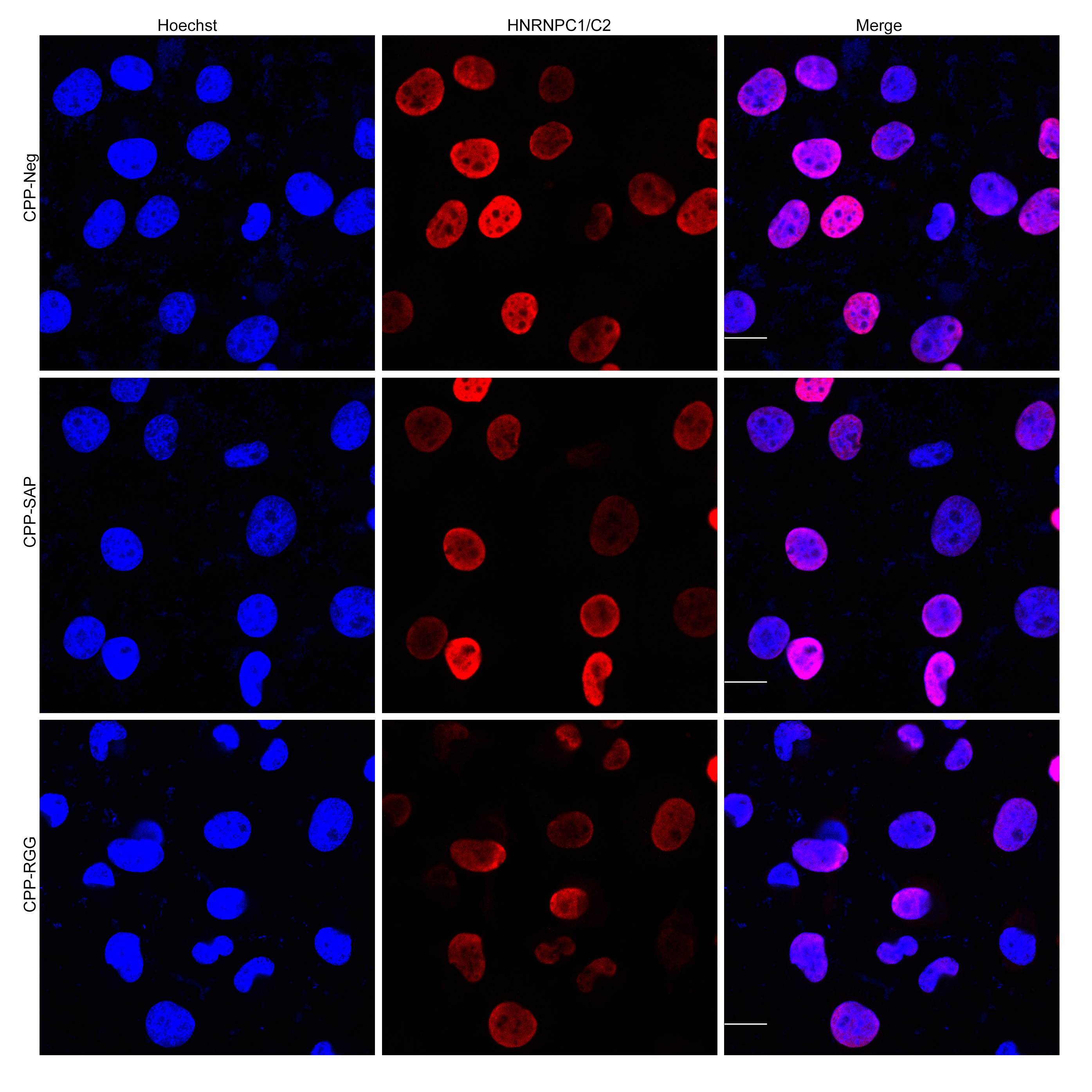

Supplement: Supplementary file 4 [file Data_Sheet_4.zip › Data Sheet 4/S. Fig. 4.2.8.jpg]

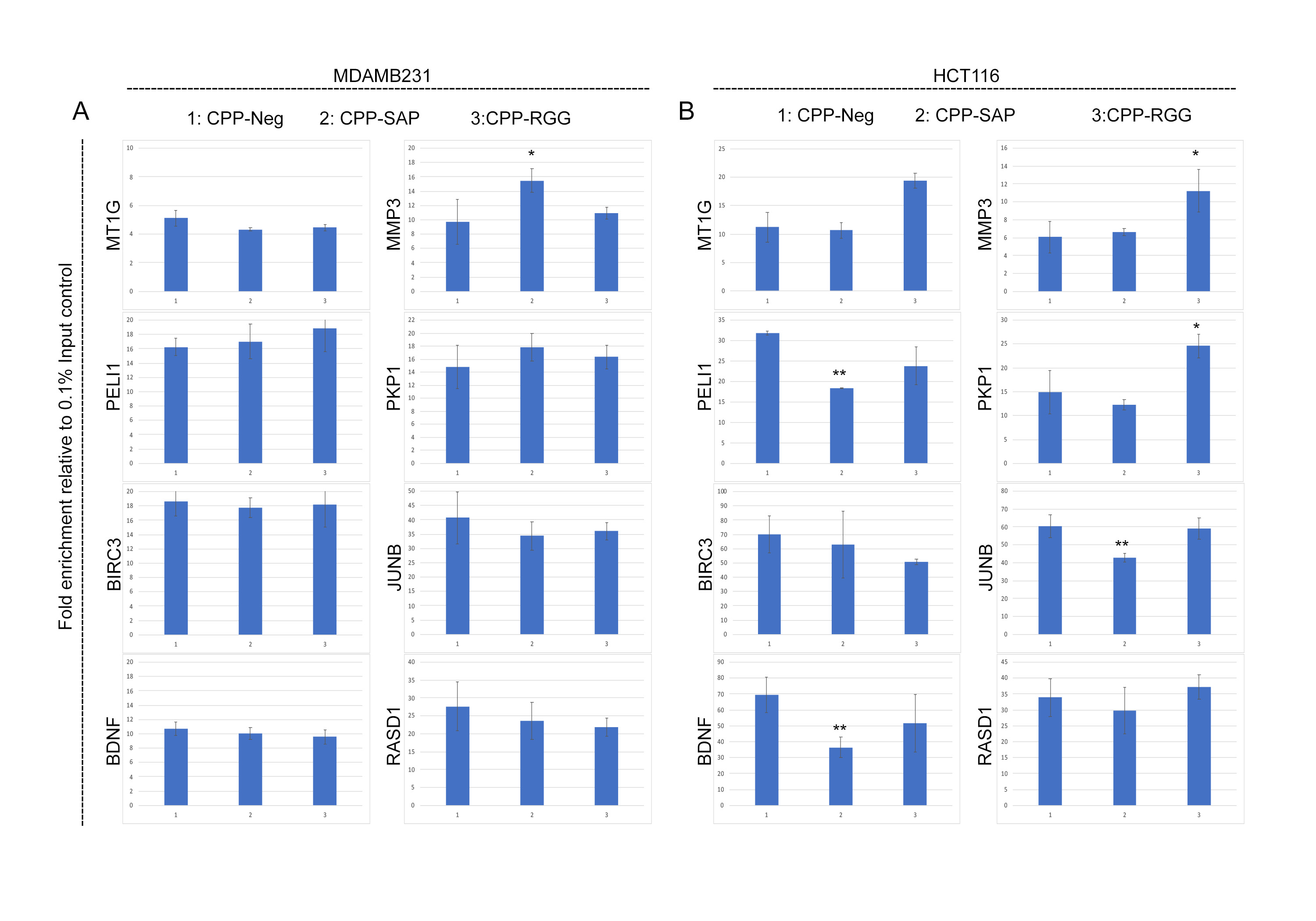

Supplement: Supplementary file 4 [file Data_Sheet_4.zip › Data Sheet 4/S. Fig. 4.3.jpg]

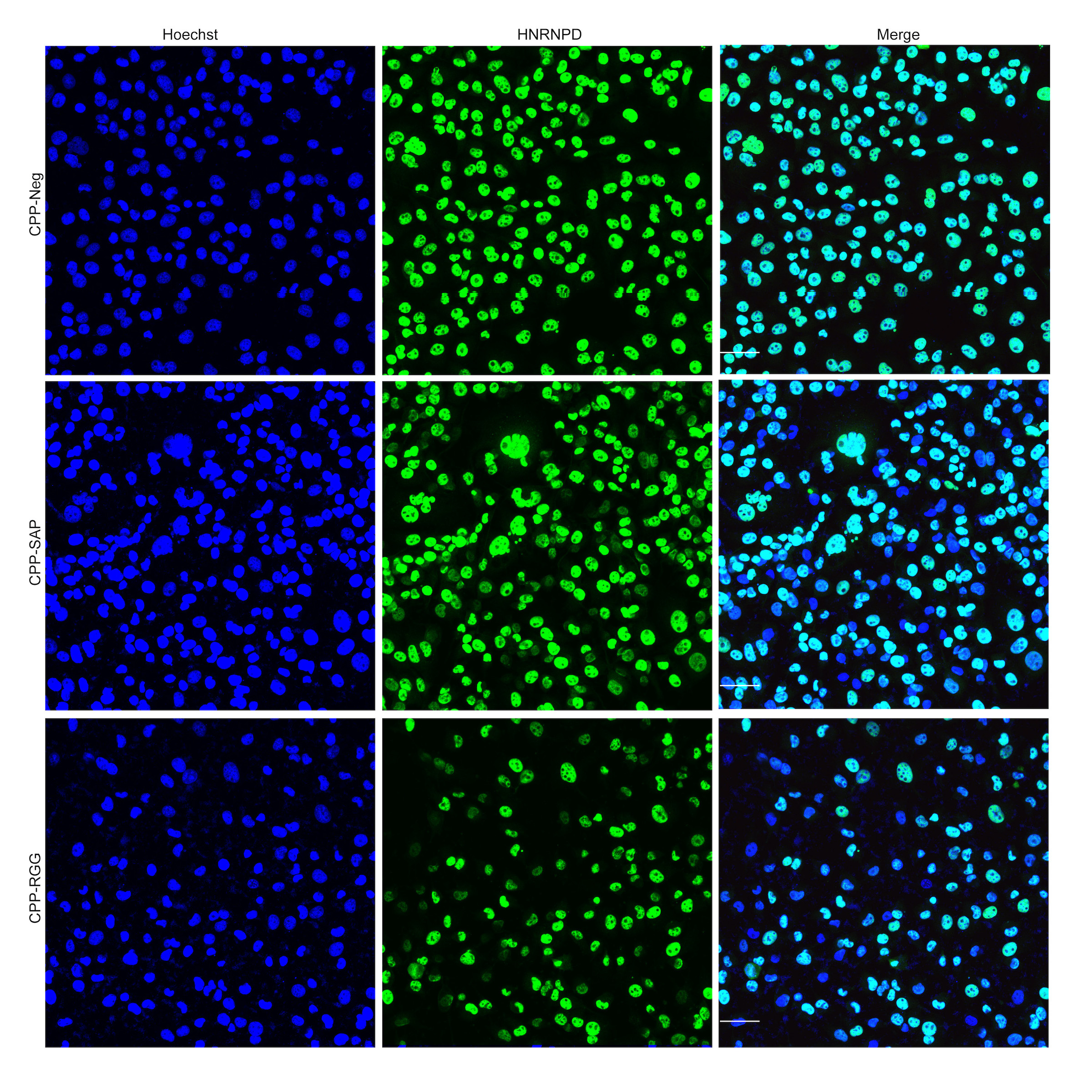

Supplement: Supplementary file 4 [file Data_Sheet_4.zip › Data Sheet 4/S. Fig. 4.2.9.jpg]

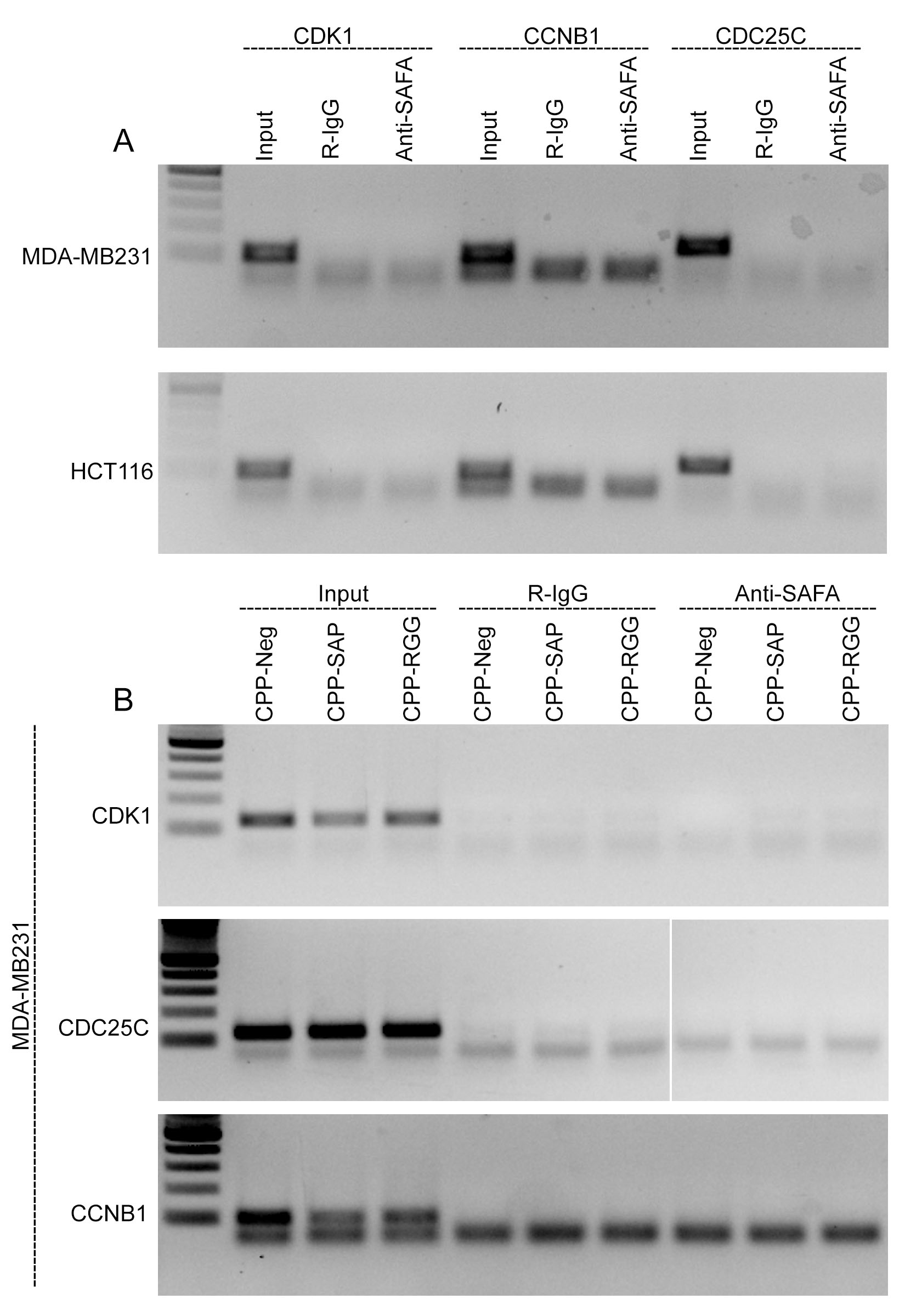

Supplement: Supplementary file 5 [file Data_Sheet_5.zip › 5/S.Fig.5.jpg]

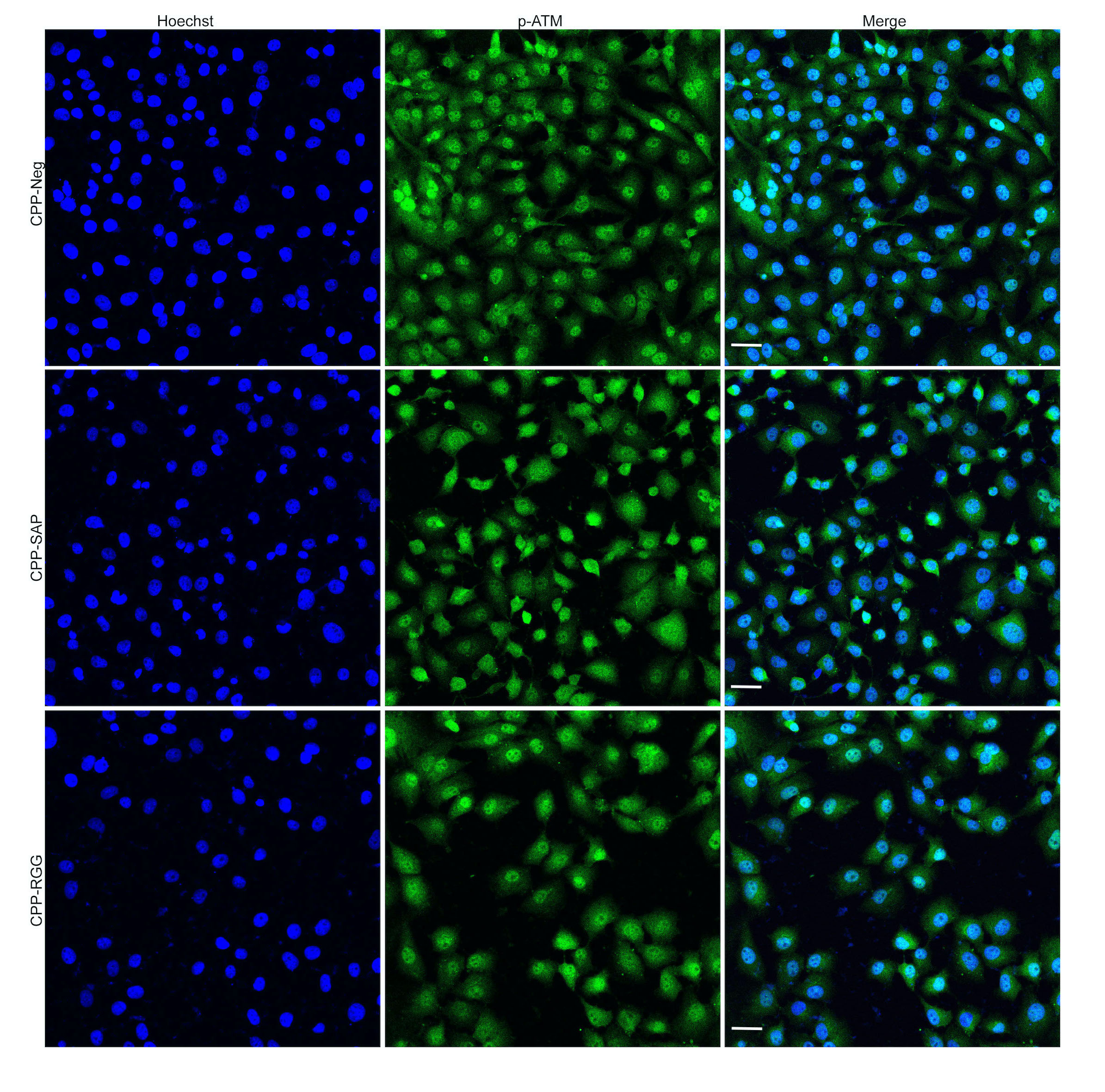

Supplement: Supplementary file 6 [file Data_Sheet_6.zip › 6/S. Fig. 6.1.5.jpg]

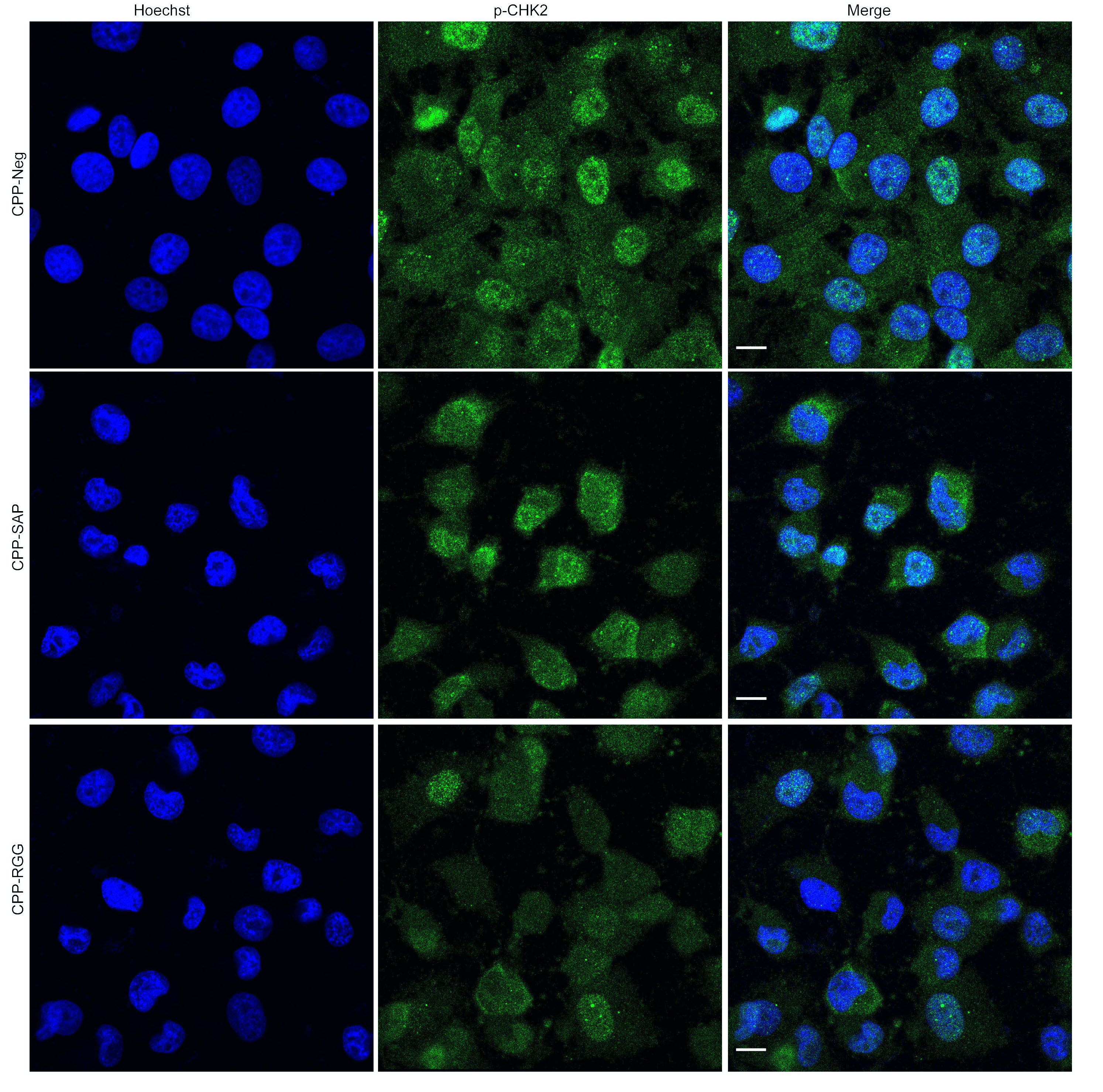

Supplement: Supplementary file 6 [file Data_Sheet_6.zip › 6/S. Fig. 6.1.4.jpg]

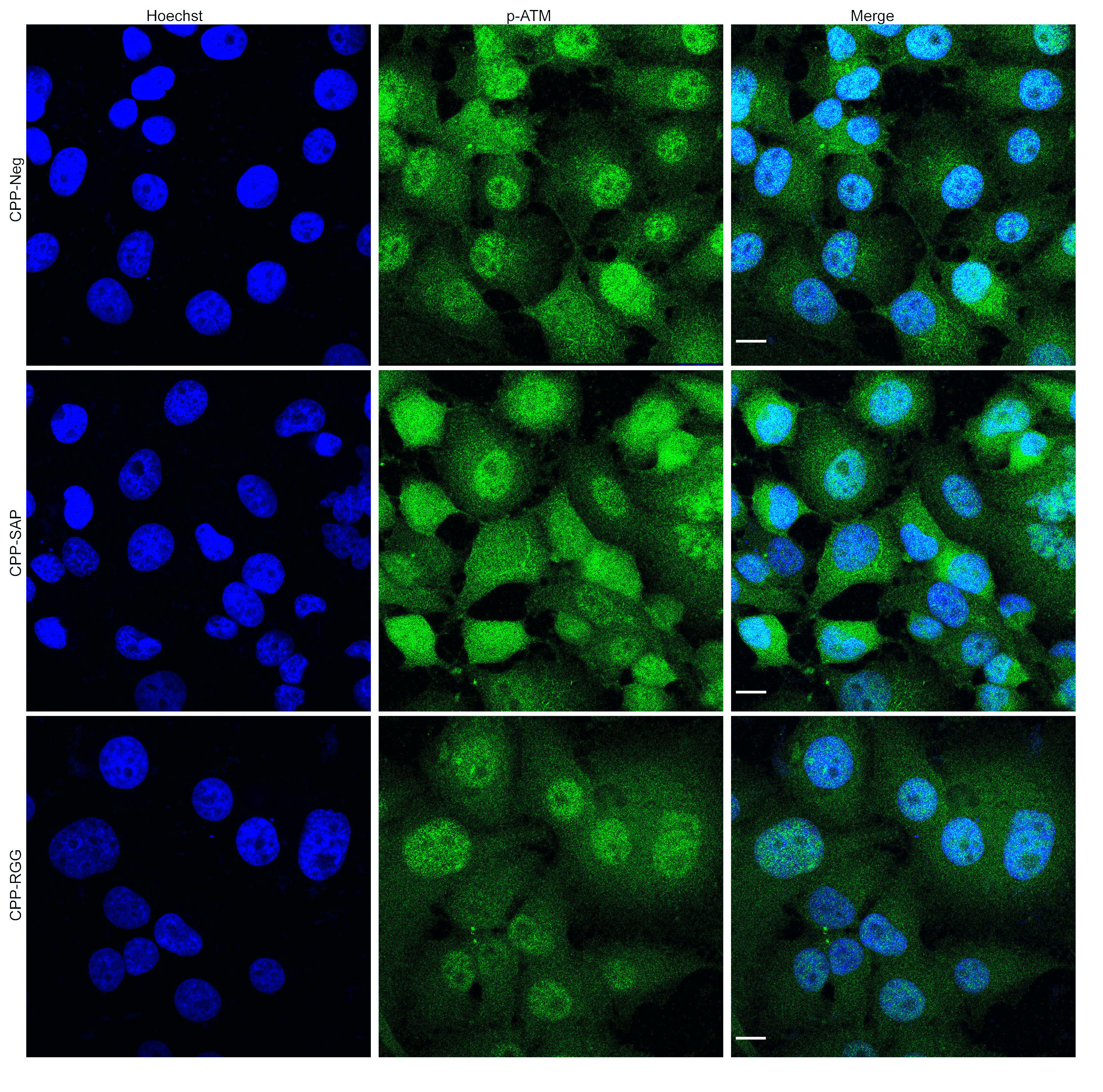

Supplement: Supplementary file 6 [file Data_Sheet_6.zip › 6/S. Fig. 6.1.6.jpg]

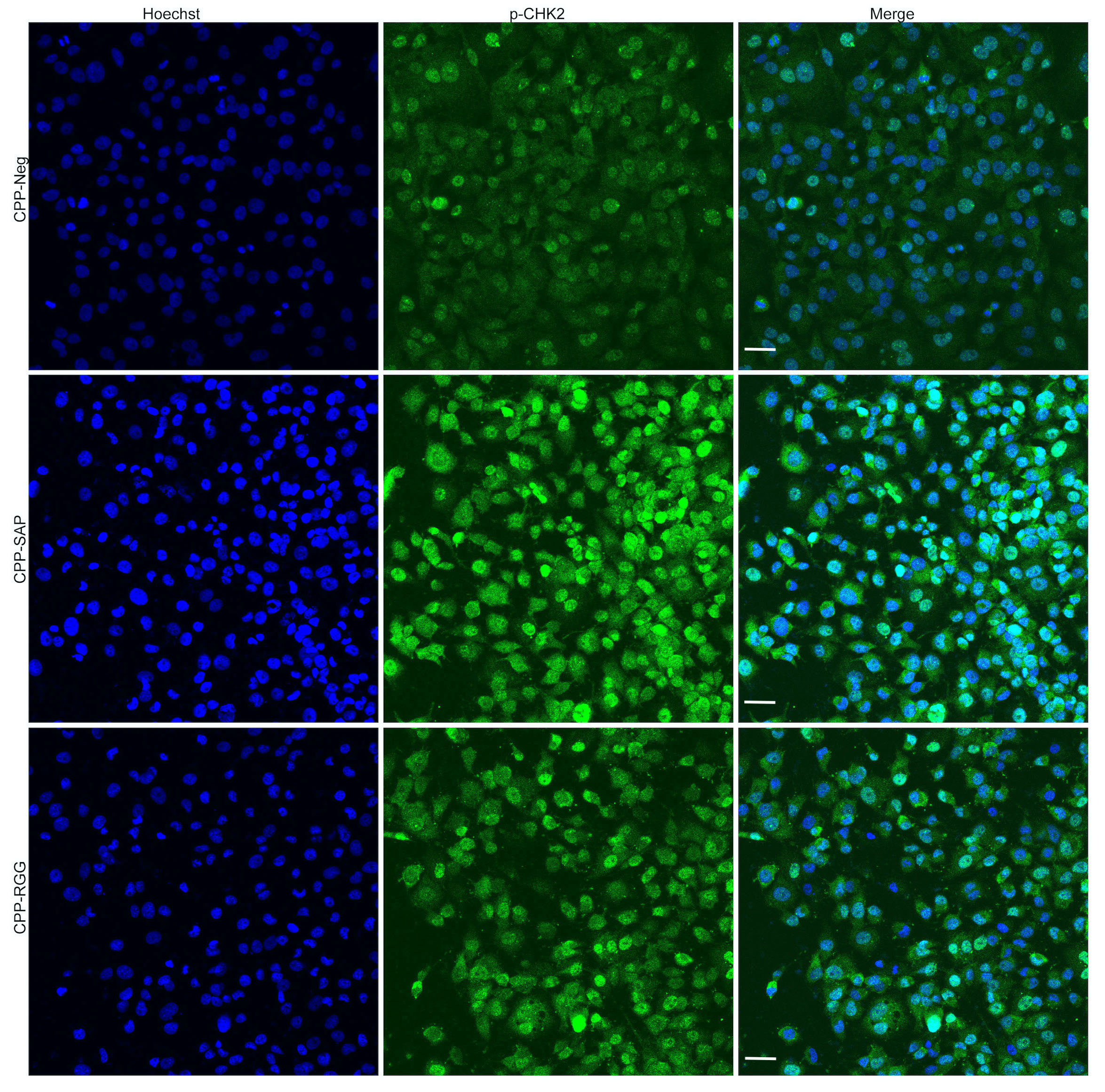

Supplement: Supplementary file 6 [file Data_Sheet_6.zip › 6/S. Fig. 6.1.3.jpg]

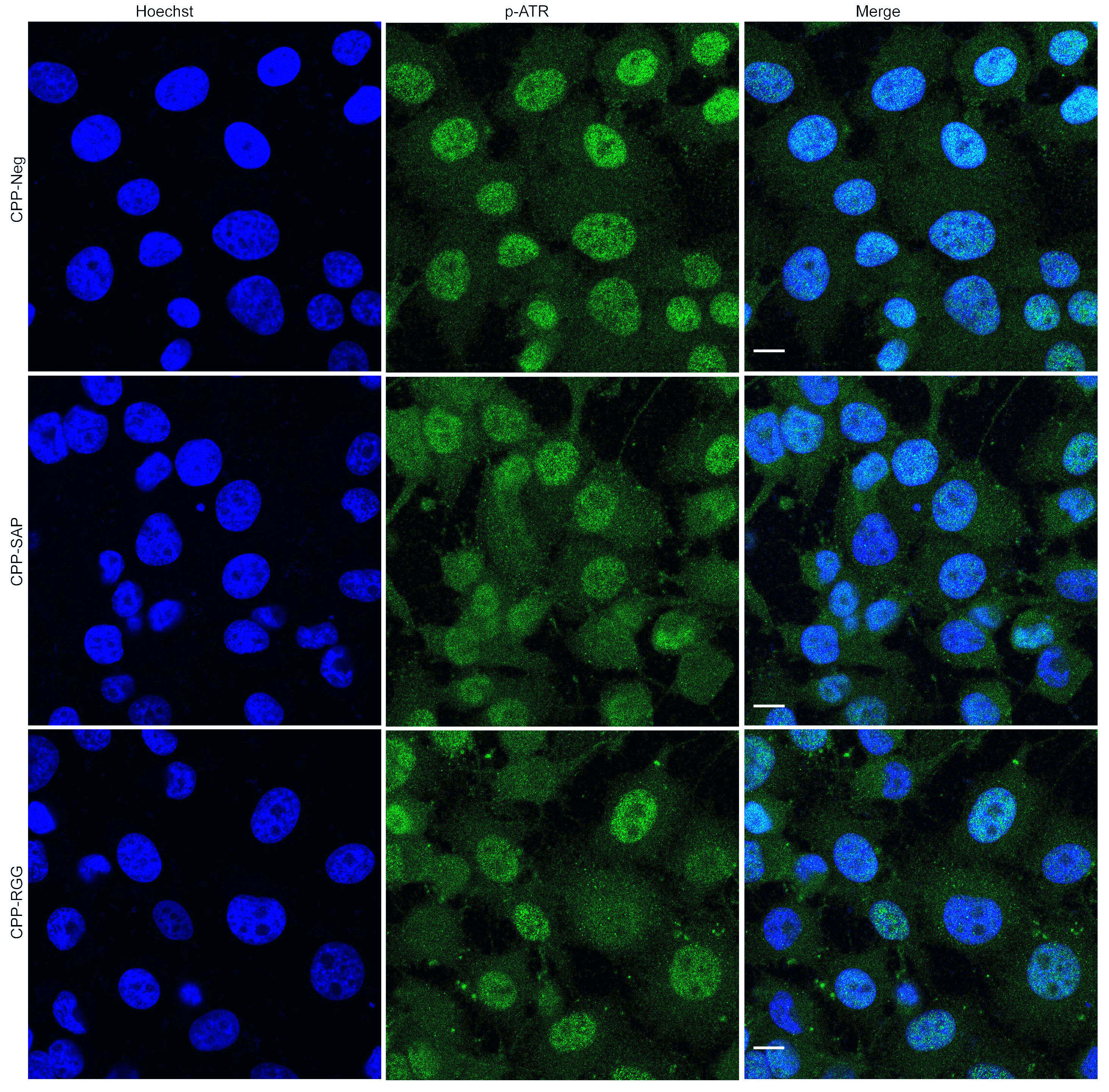

Supplement: Supplementary file 6 [file Data_Sheet_6.zip › 6/S. Fig. 6.1.2.jpg]

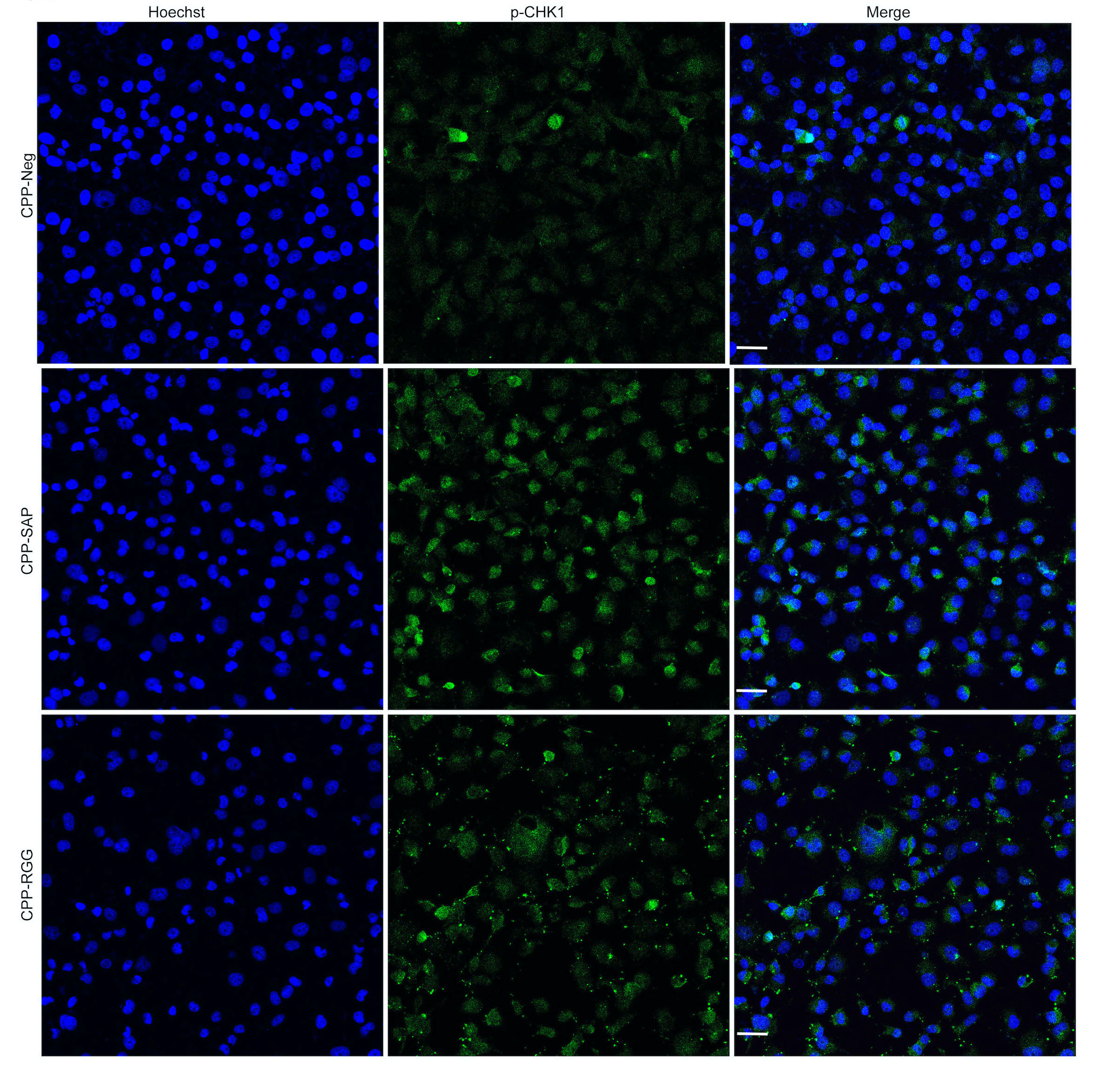

Supplement: Supplementary file 6 [file Data_Sheet_6.zip › 6/S. Fig. 6.1.7 .jpg]

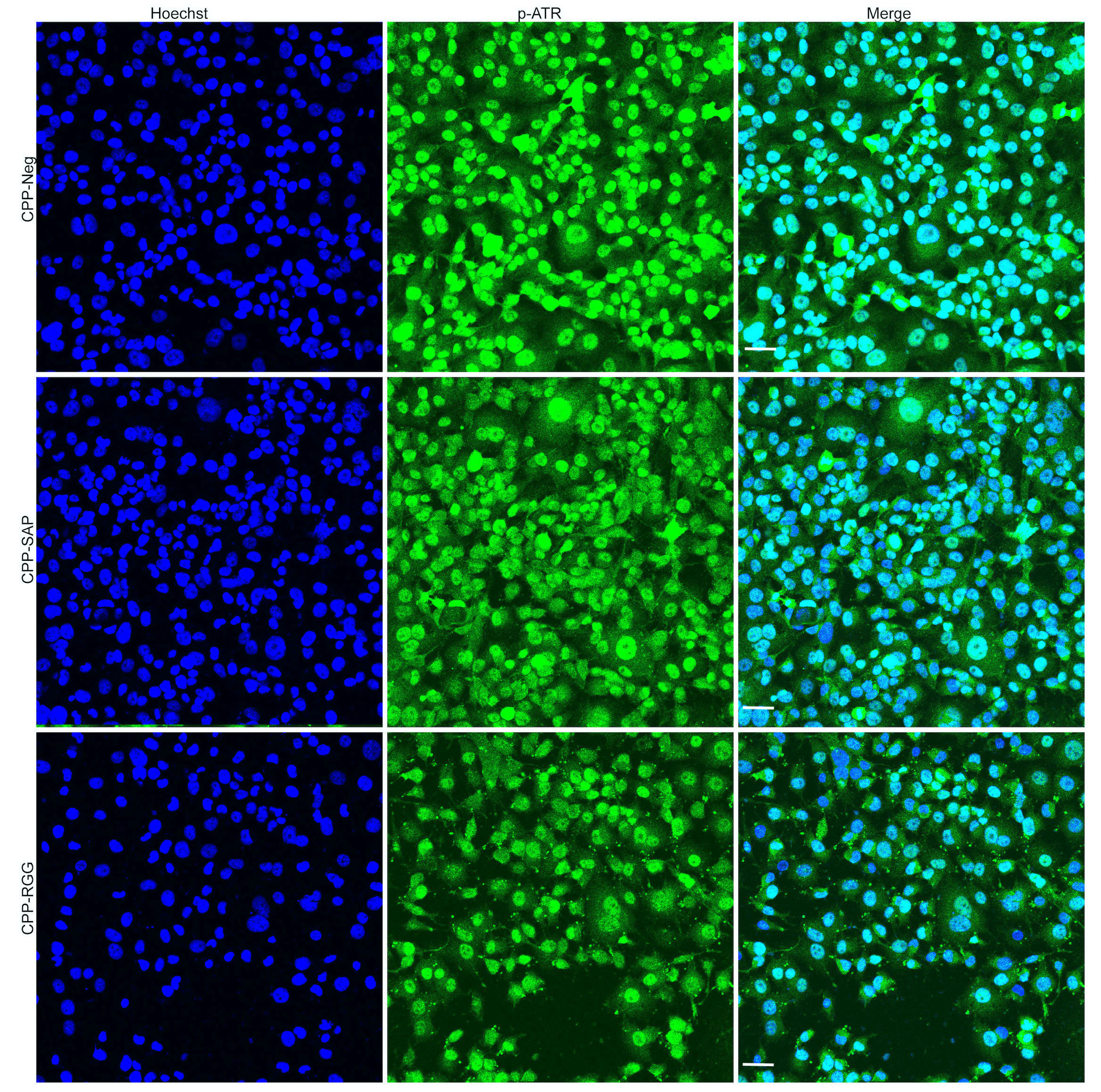

Supplement: Supplementary file 6 [file Data_Sheet_6.zip › 6/S. Fig. 6.1.1.jpg]

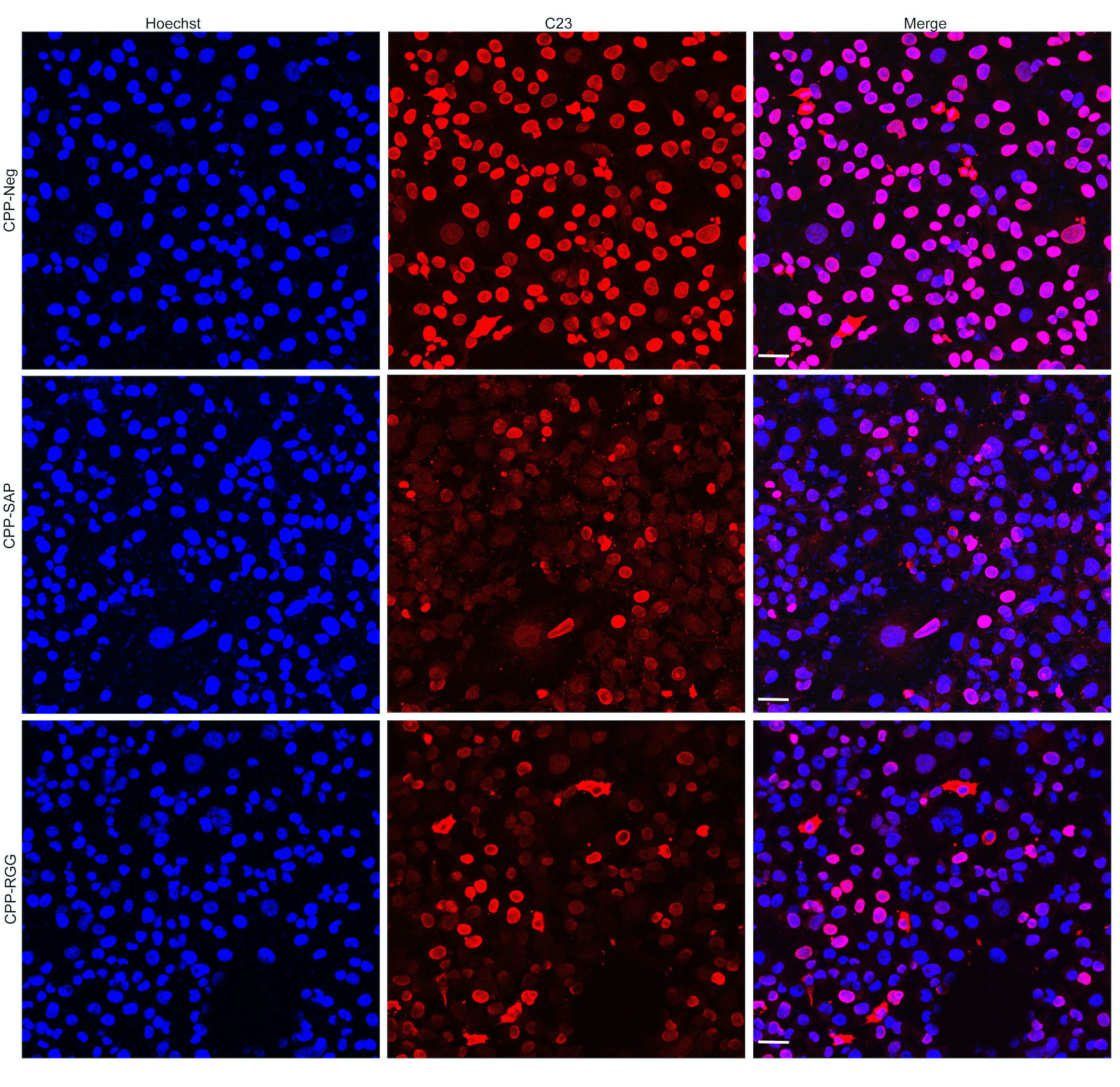

Supplement: Supplementary file 6 [file Data_Sheet_6.zip › 6/S. Fig. 6.1.11.jpg]

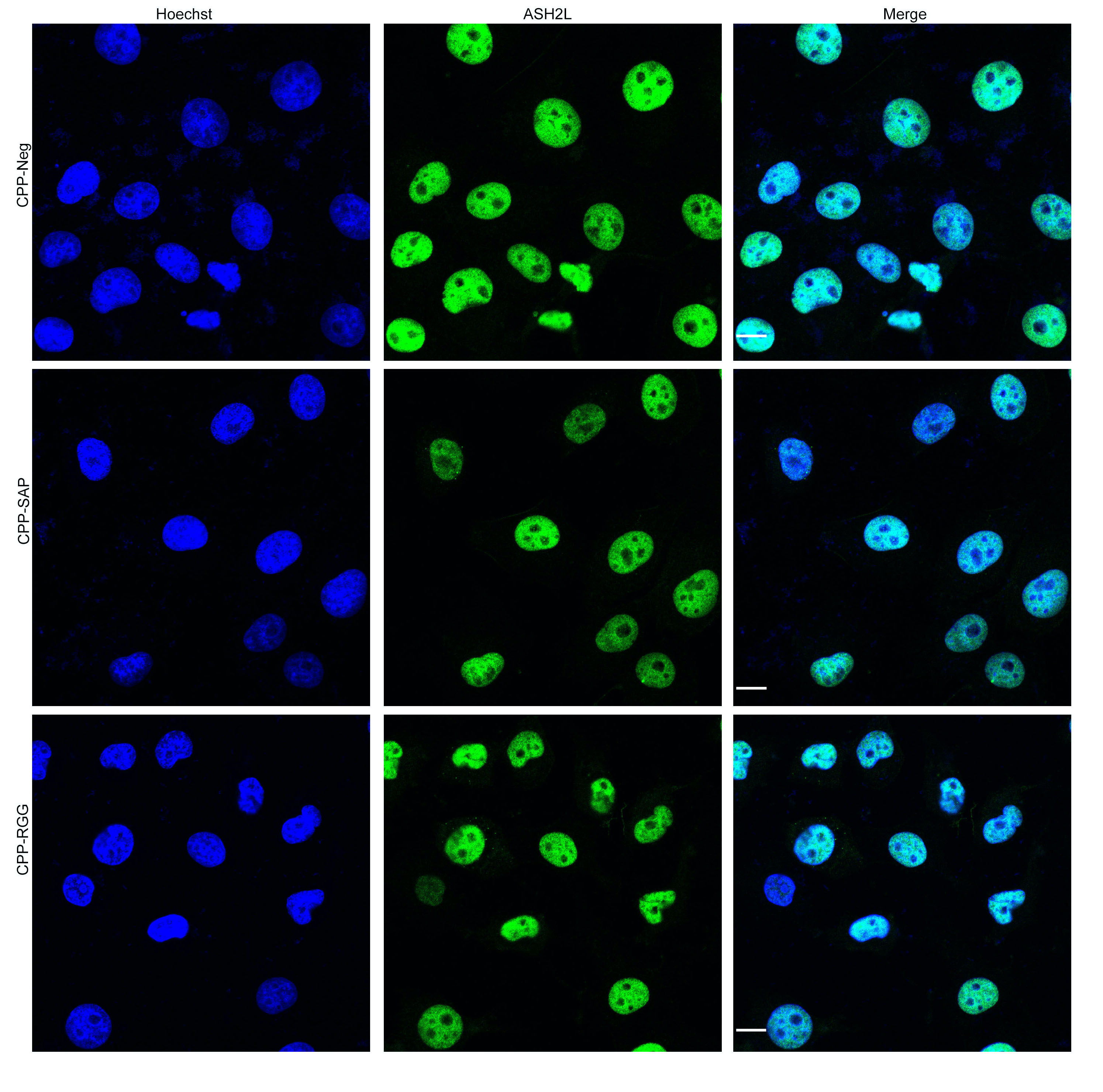

Supplement: Supplementary file 6 [file Data_Sheet_6.zip › 6/S. Fig. 6.1.10.jpg]

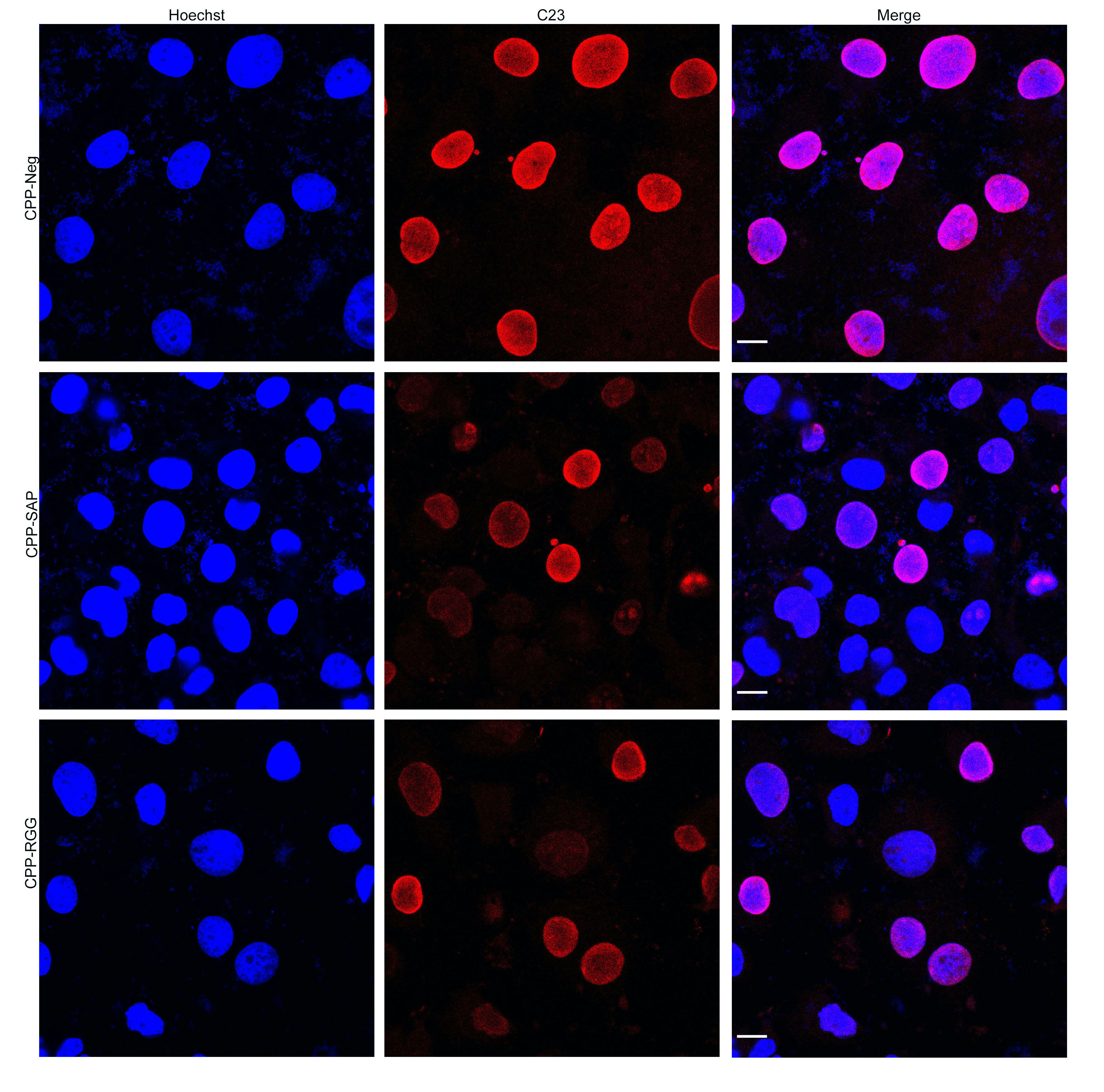

Supplement: Supplementary file 6 [file Data_Sheet_6.zip › 6/S. Fig. 6.1.12.jpg]

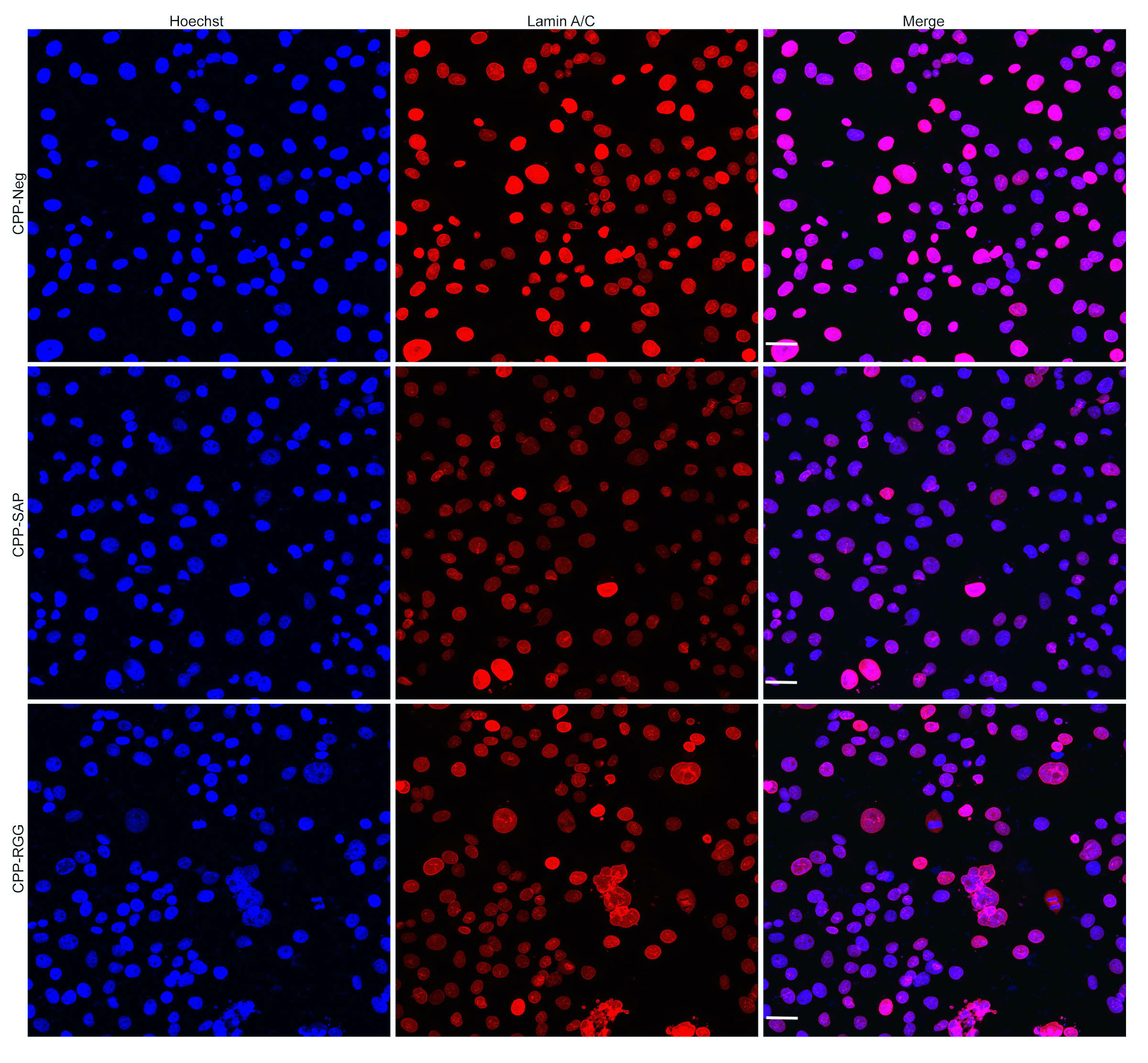

Supplement: Supplementary file 6 [file Data_Sheet_6.zip › 6/S. Fig. 6.1.13.jpg]

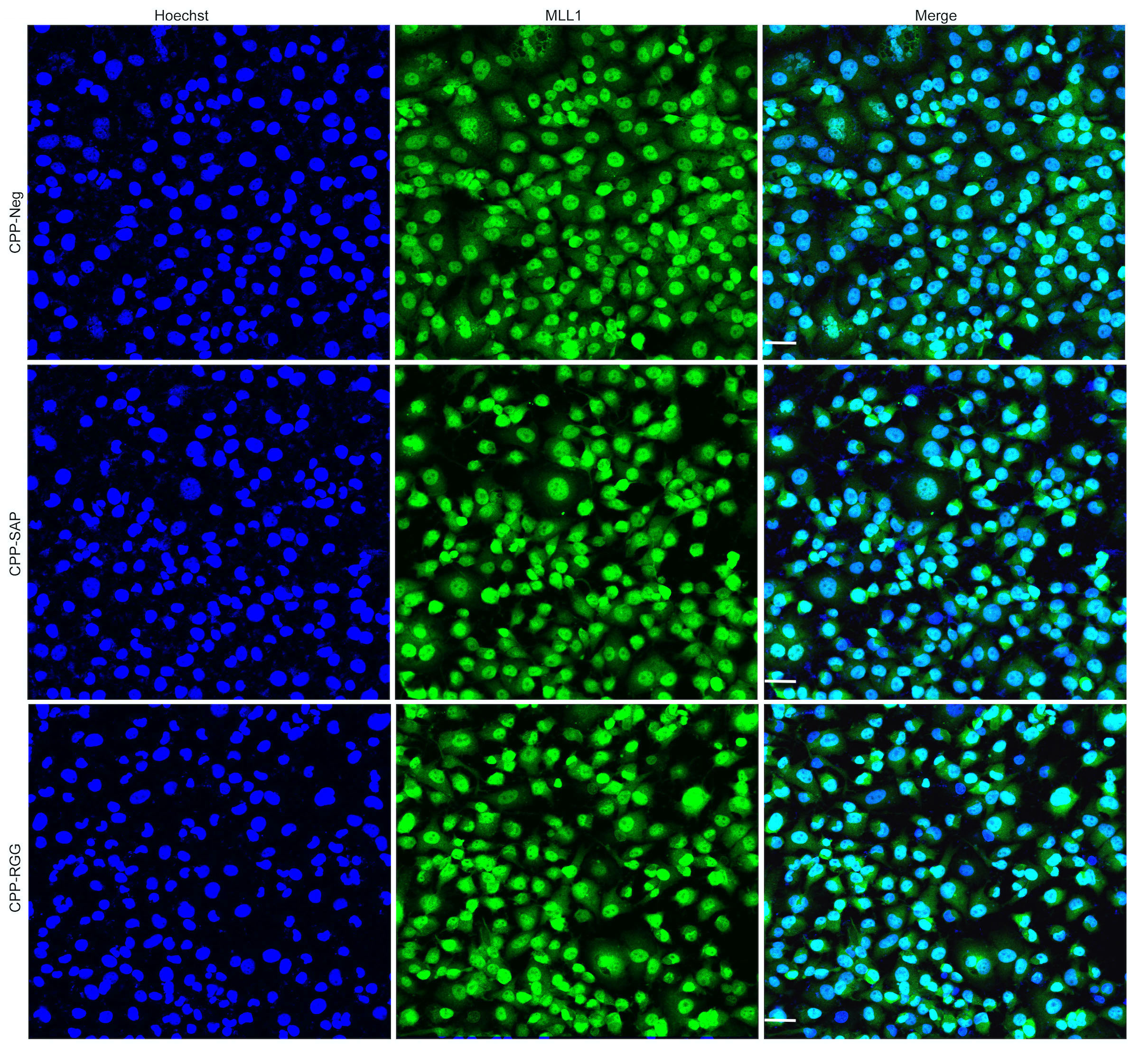

Supplement: Supplementary file 6 [file Data_Sheet_6.zip › 6/S. Fig. 6.1.17.jpg]

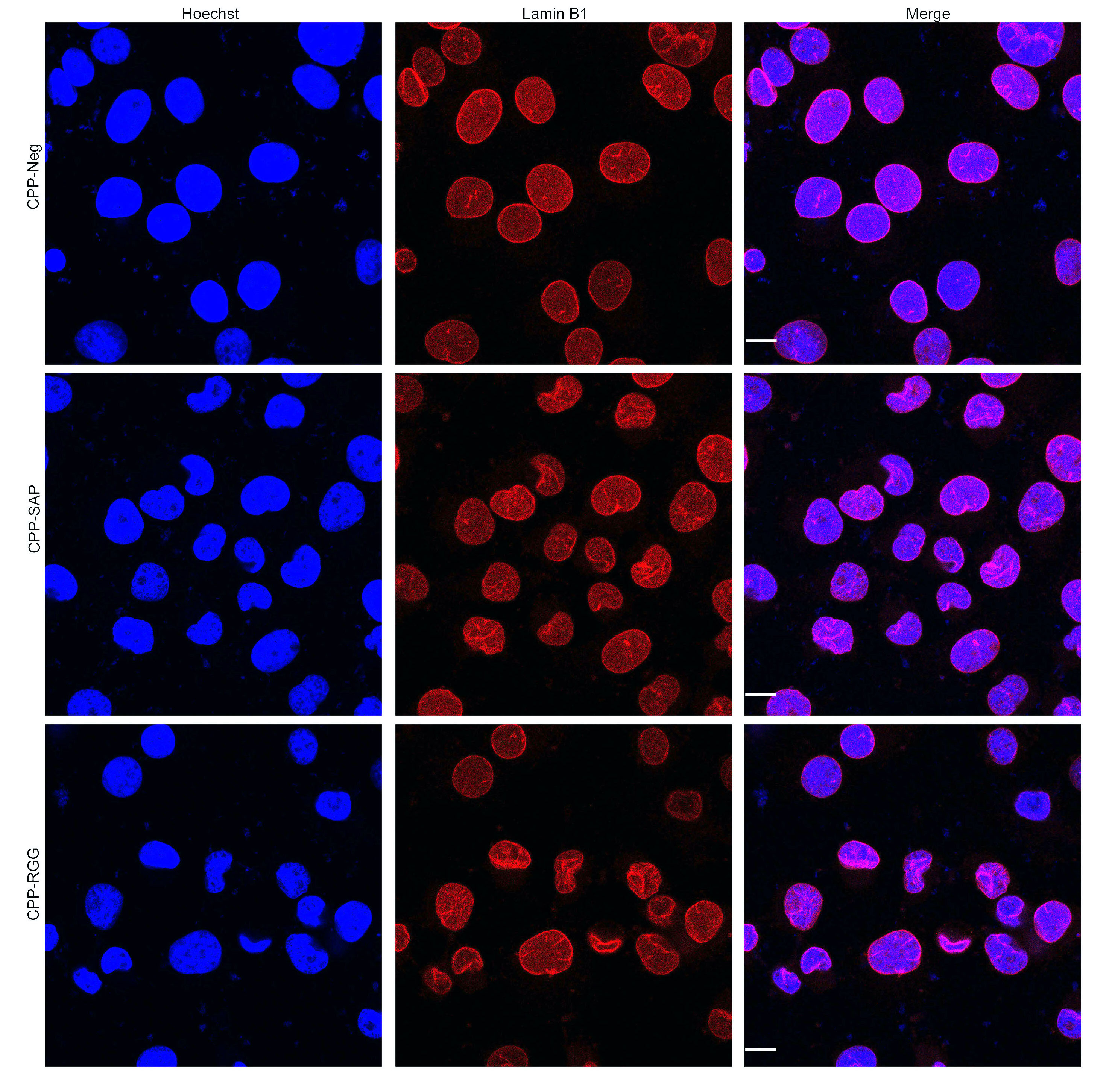

Supplement: Supplementary file 6 [file Data_Sheet_6.zip › 6/S. Fig. 6.1.16.jpg]

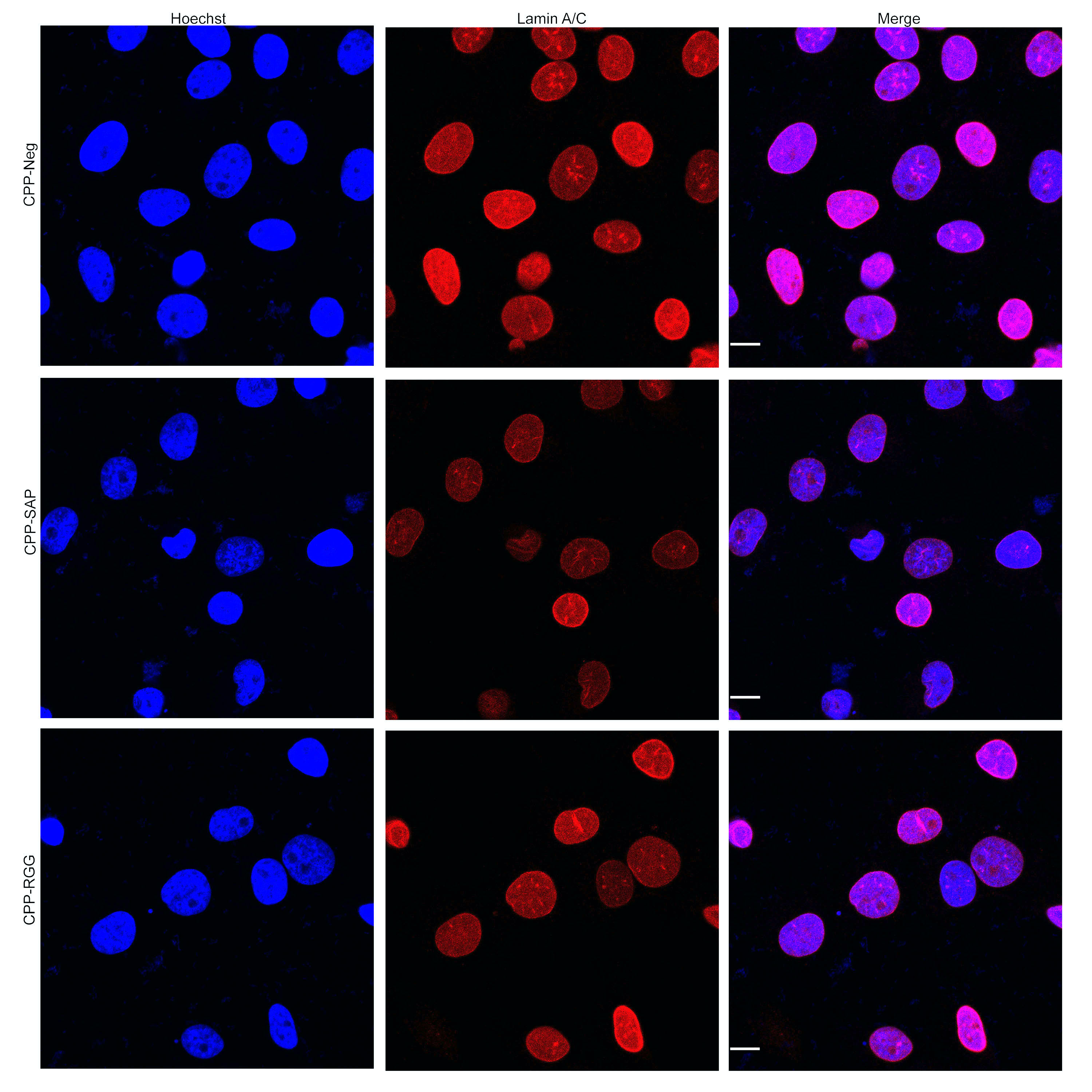

Supplement: Supplementary file 6 [file Data_Sheet_6.zip › 6/S. Fig. 6.1.14.jpg]

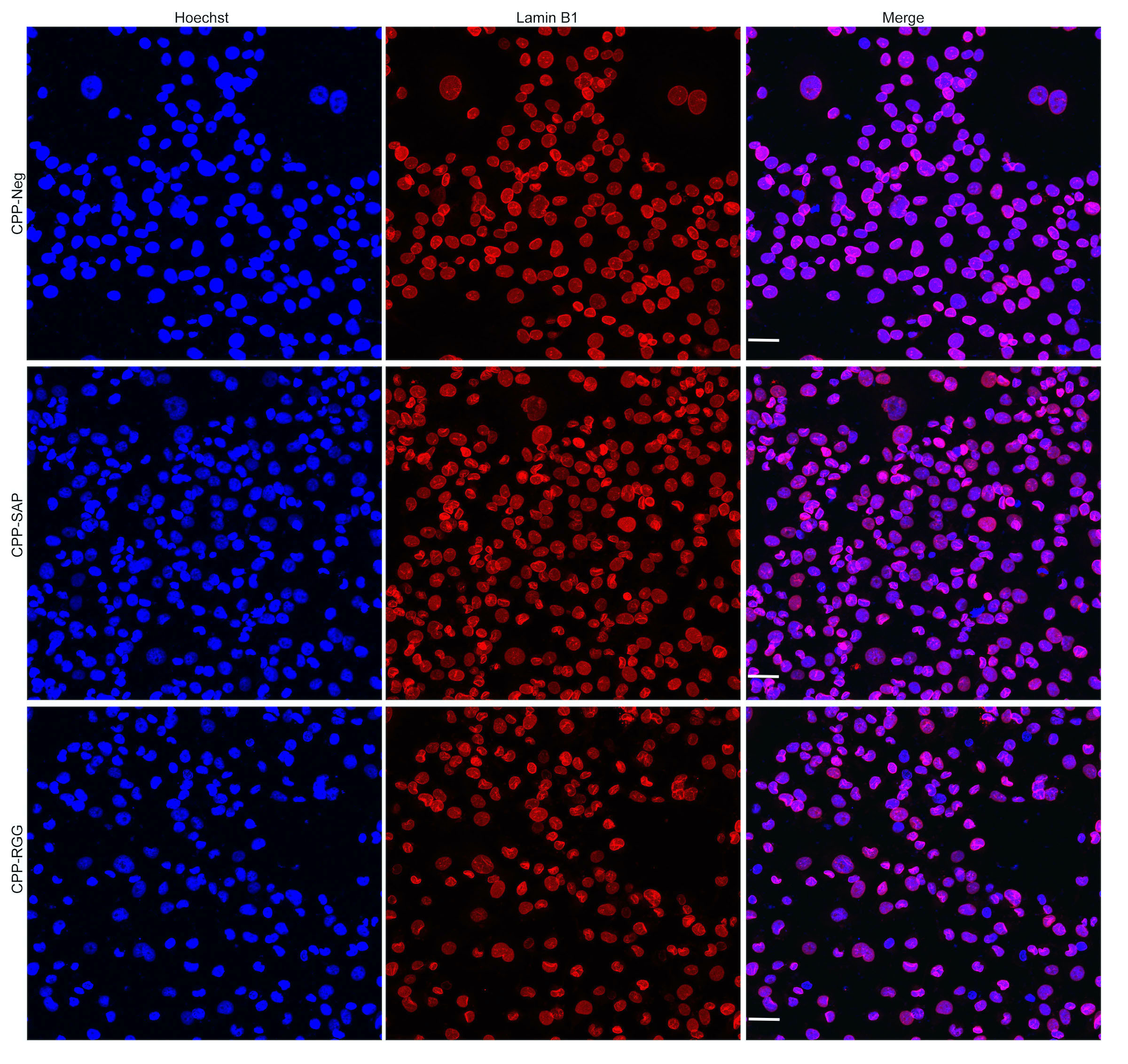

Supplement: Supplementary file 6 [file Data_Sheet_6.zip › 6/S. Fig. 6.1.15.jpg]

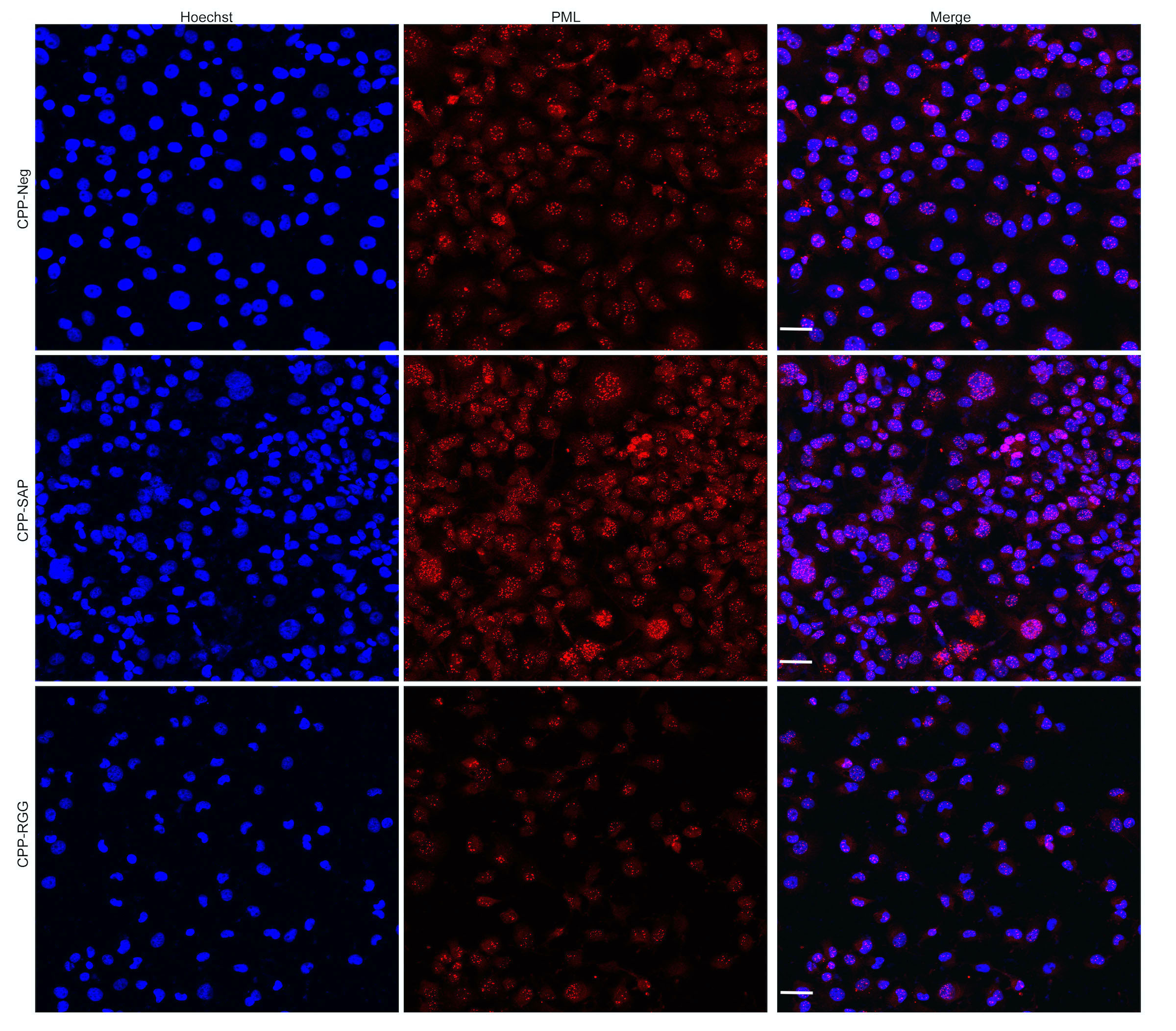

Supplement: Supplementary file 6 [file Data_Sheet_6.zip › 6/S. Fig. 6.1.18.jpg]

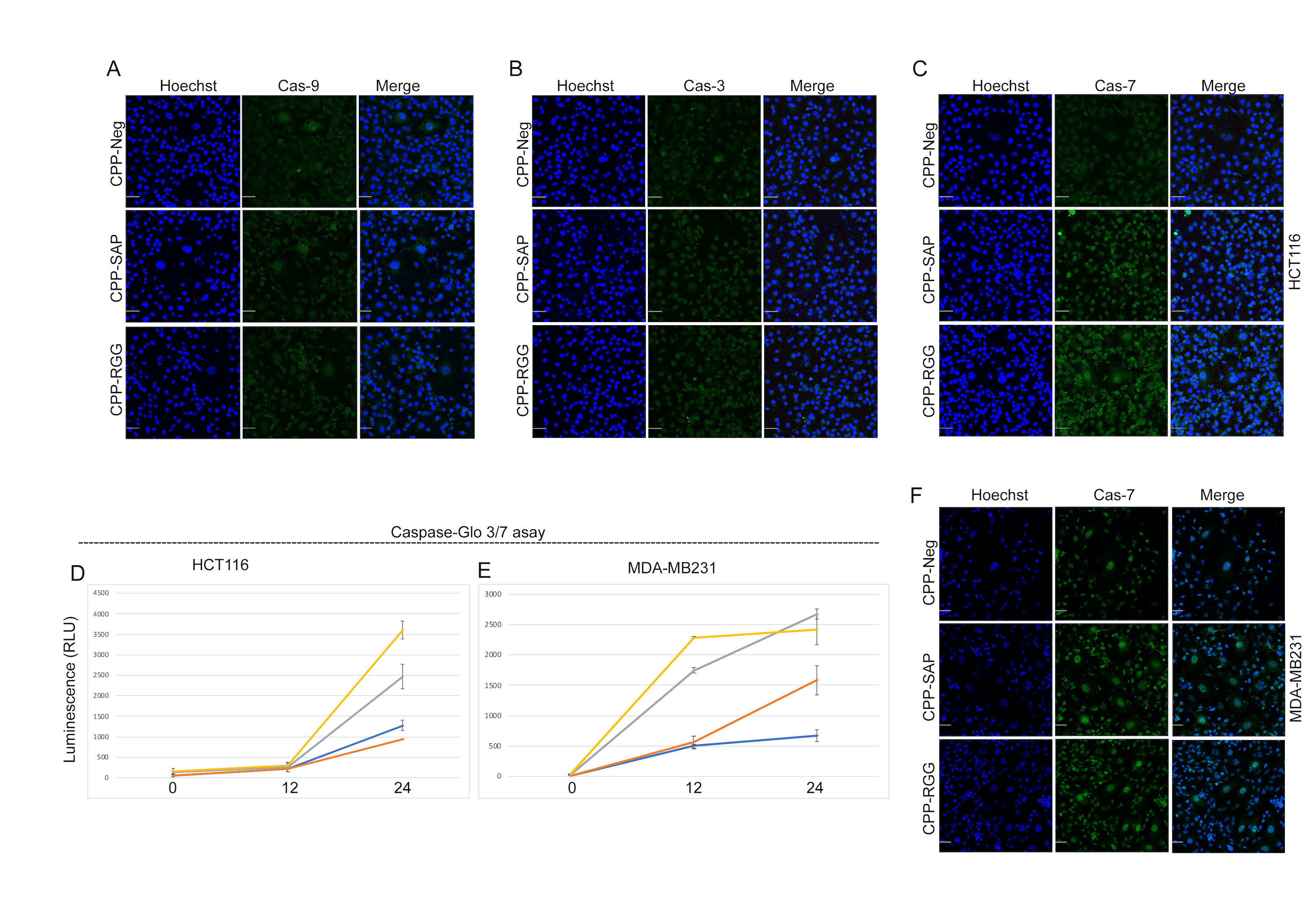

Supplement: Supplementary file 6 [file Data_Sheet_6.zip › 6/S. Fig. 6. 2.jpg]

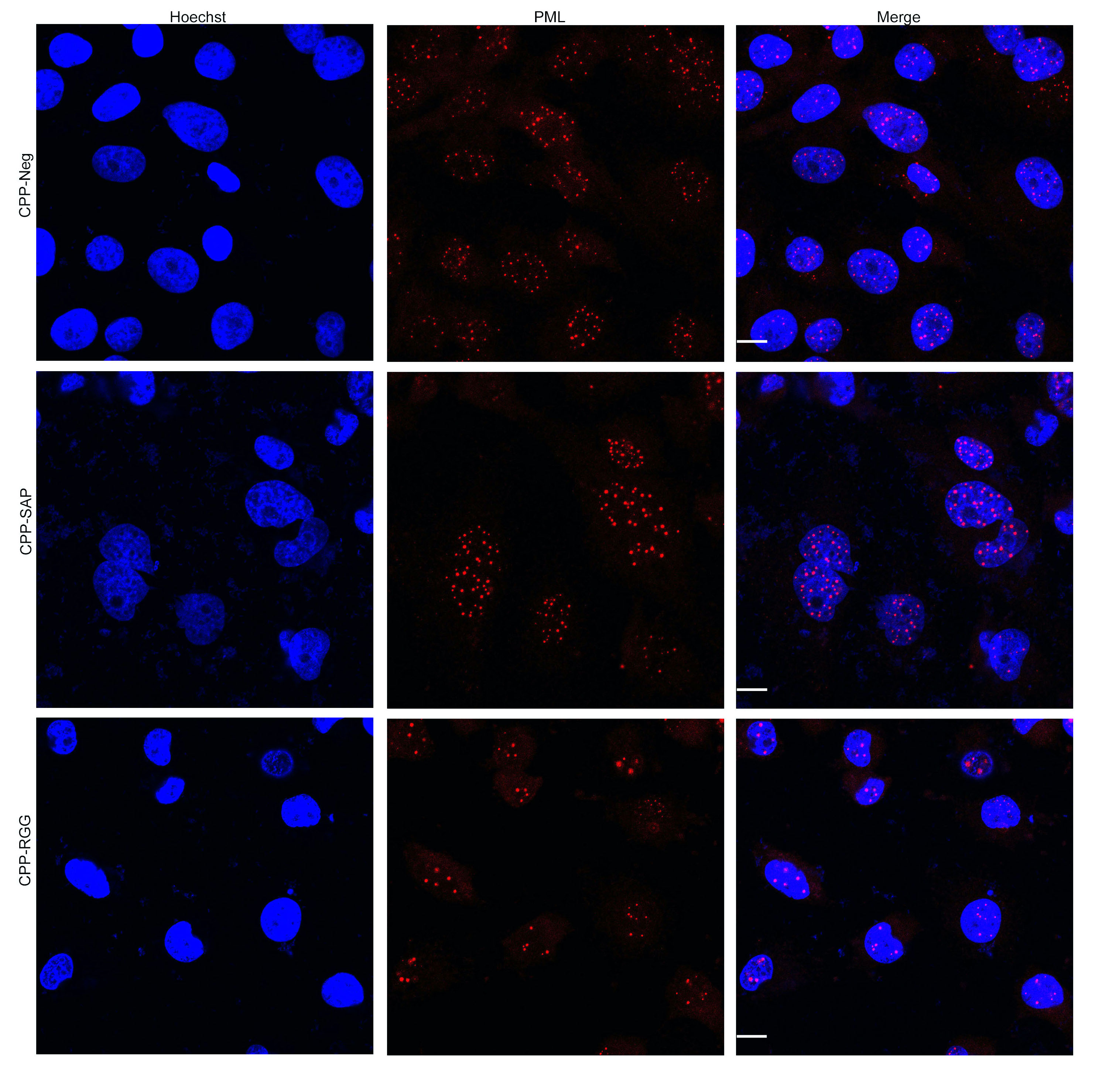

Supplement: Supplementary file 6 [file Data_Sheet_6.zip › 6/S. Fig. 6.1.19 .jpg]

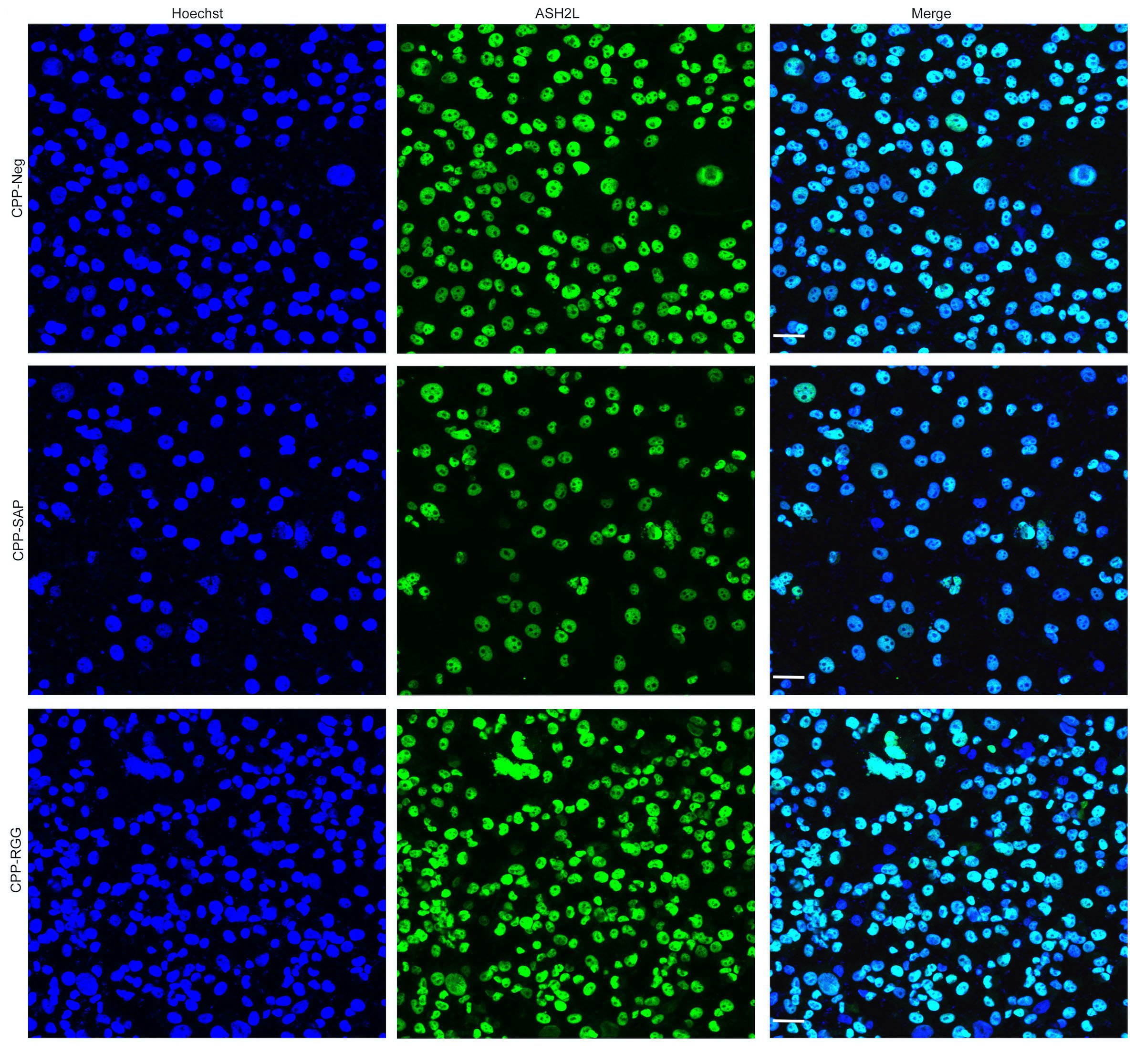

Supplement: Supplementary file 6 [file Data_Sheet_6.zip › 6/S. Fig. 6.1.9.jpg]

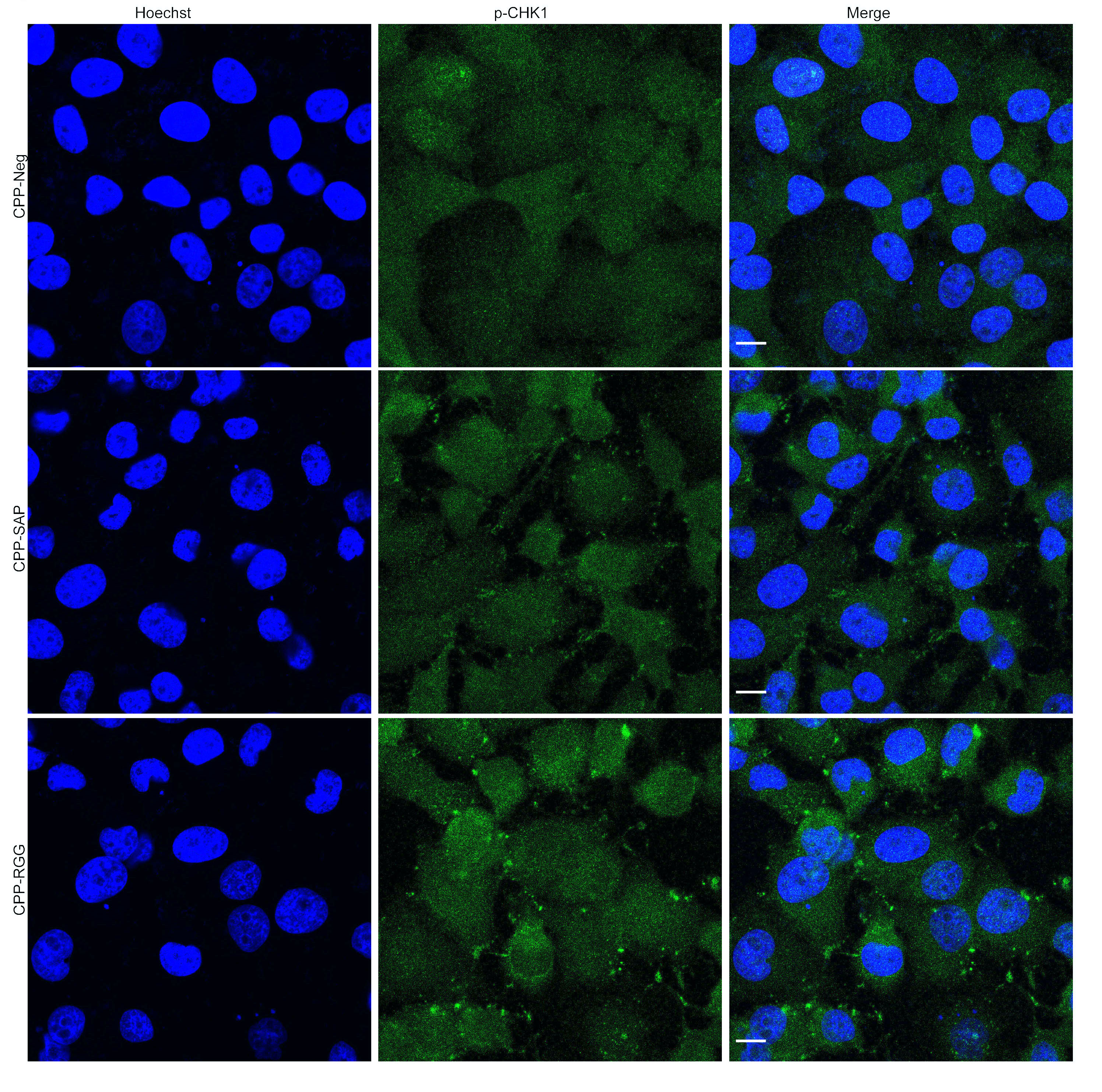

Supplement: Supplementary file 6 [file Data_Sheet_6.zip › 6/S. Fig. 6.1.8.jpg]

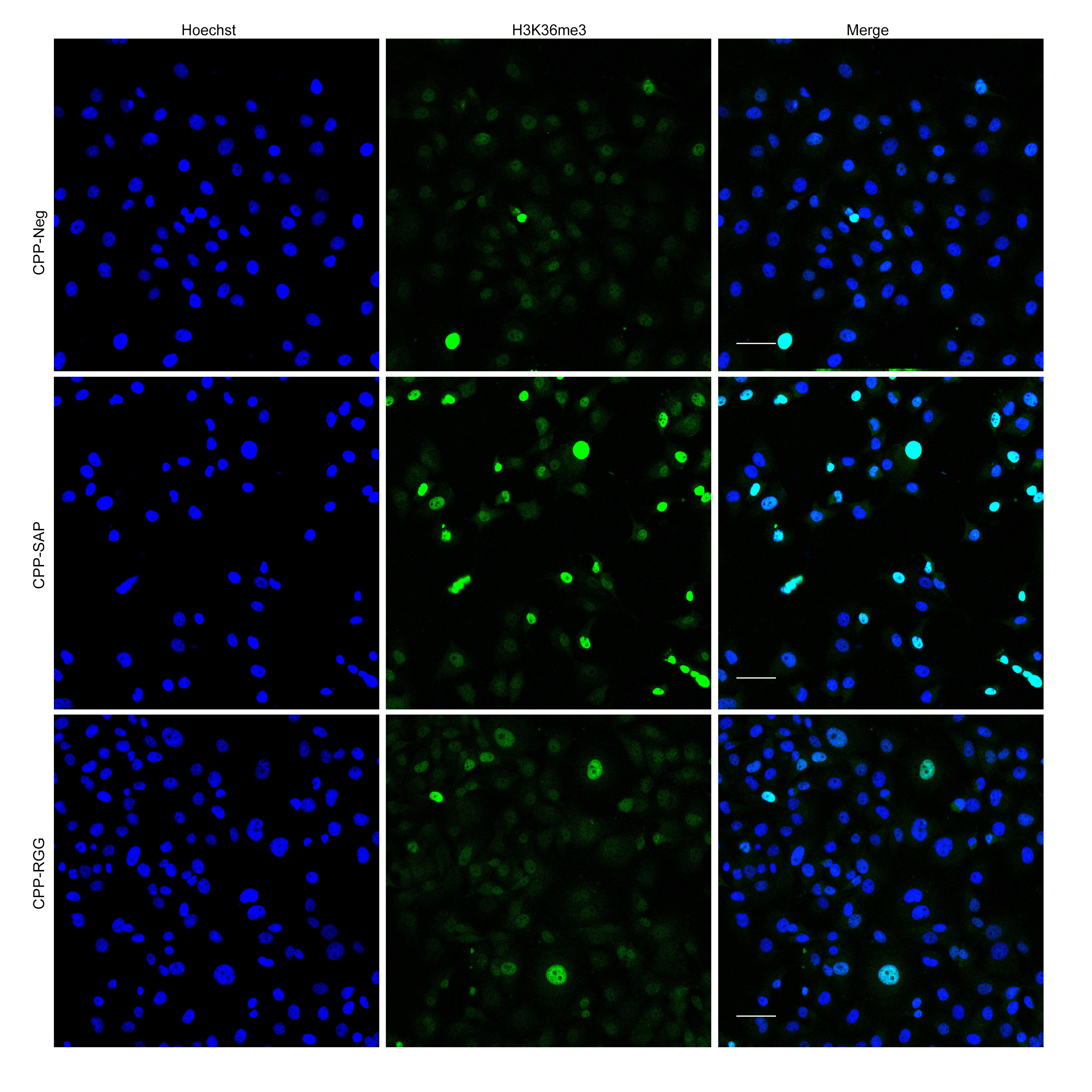

Supplement: Supplementary file 7 [file Data_Sheet_7.zip › Data Sheet 7/S. Fig. 7.9.jpg]

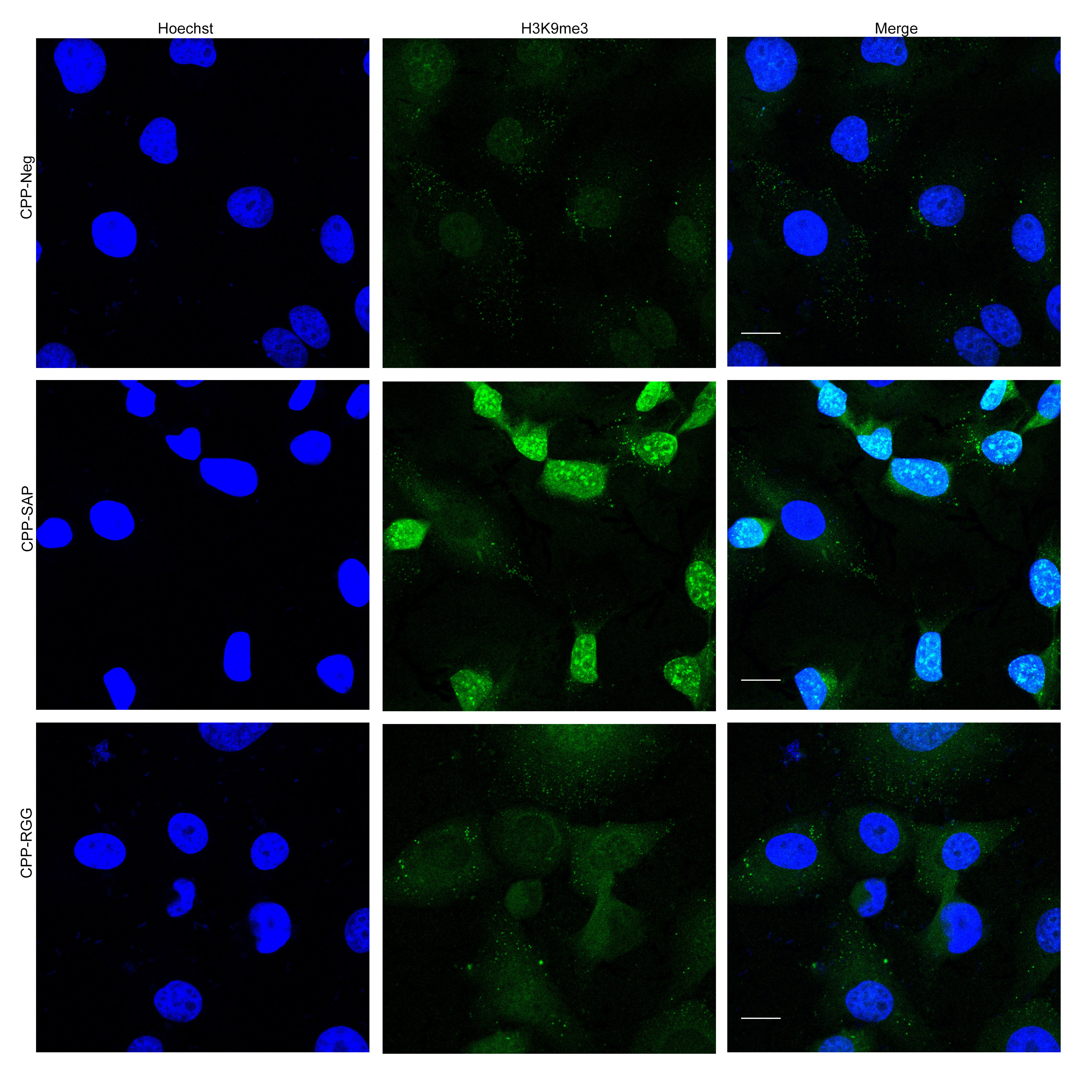

Supplement: Supplementary file 7 [file Data_Sheet_7.zip › Data Sheet 7/S. Fig. 7.8.jpg]

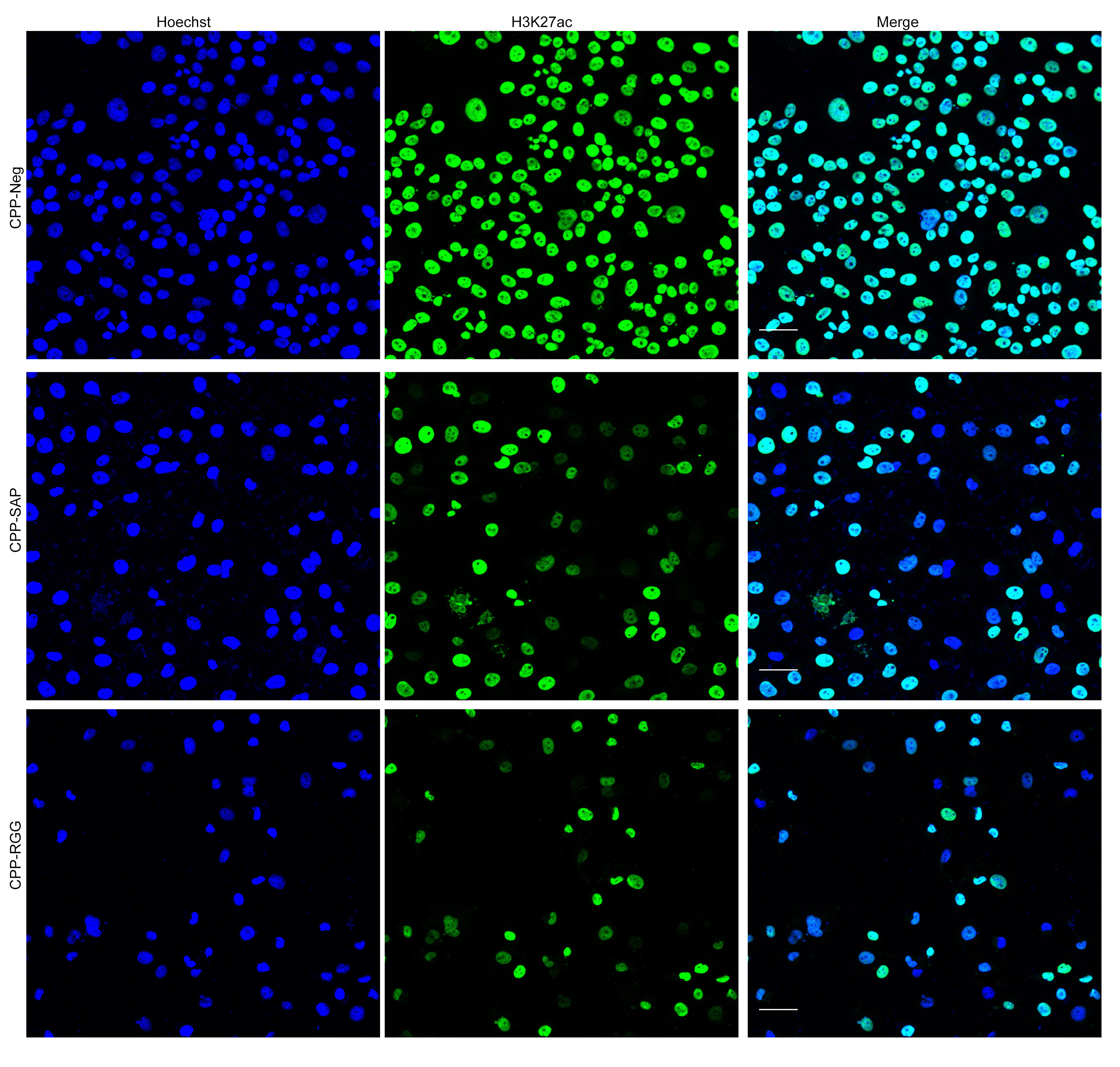

Supplement: Supplementary file 7 [file Data_Sheet_7.zip › Data Sheet 7/S. Fig. 7.15.jpg]

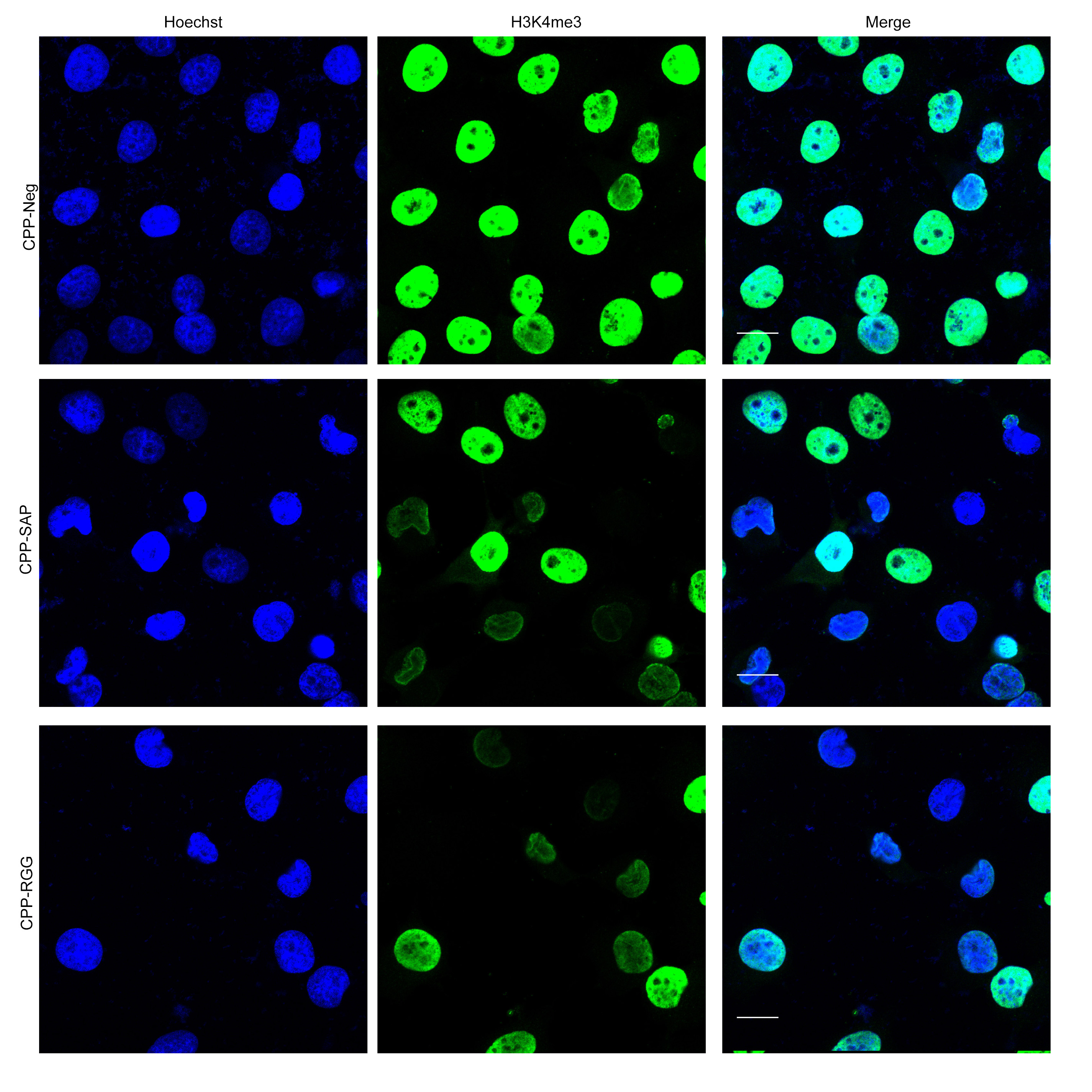

Supplement: Supplementary file 7 [file Data_Sheet_7.zip › Data Sheet 7/S. Fig. 7.14.jpg]

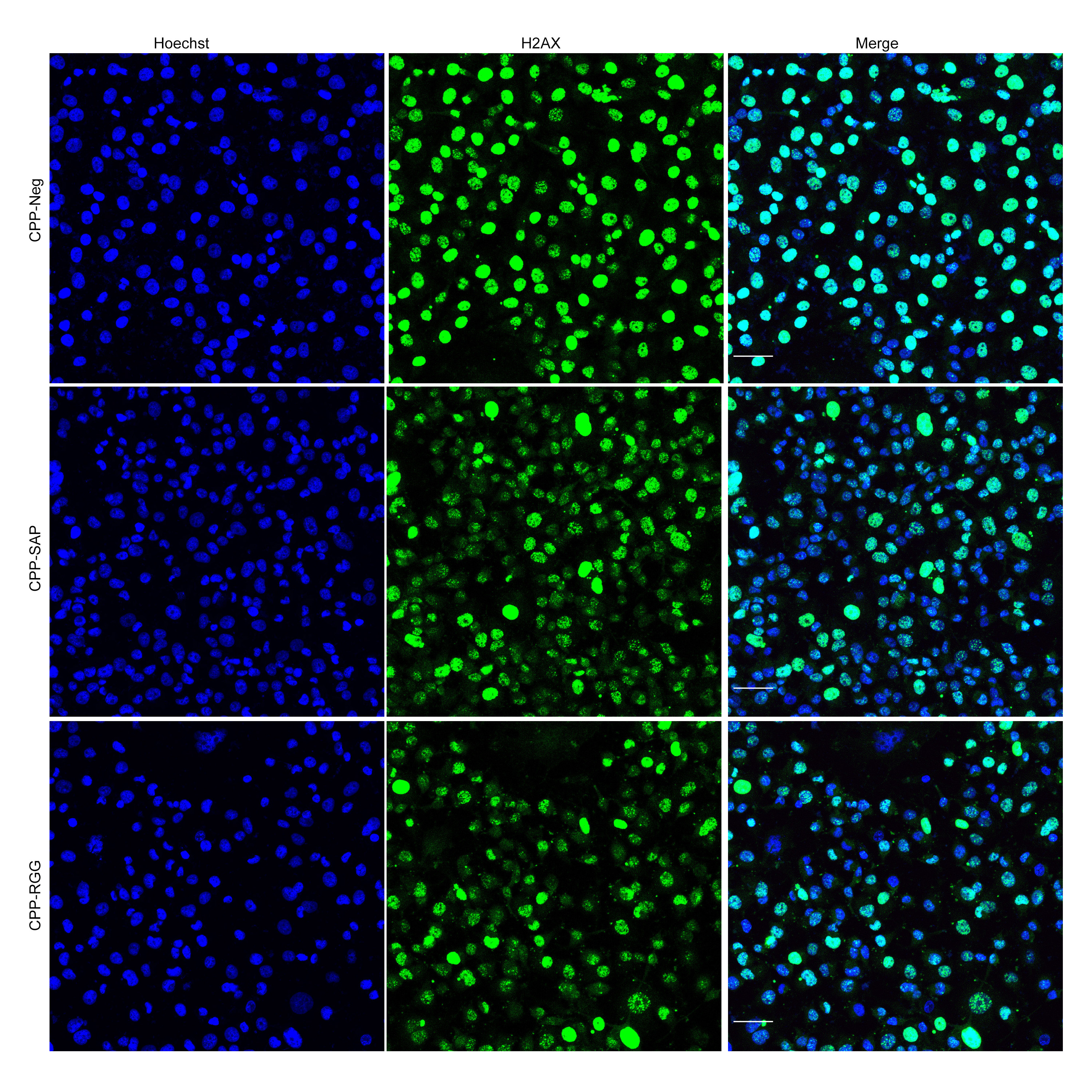

Supplement: Supplementary file 7 [file Data_Sheet_7.zip › Data Sheet 7/S. Fig. 7.1.jpg]

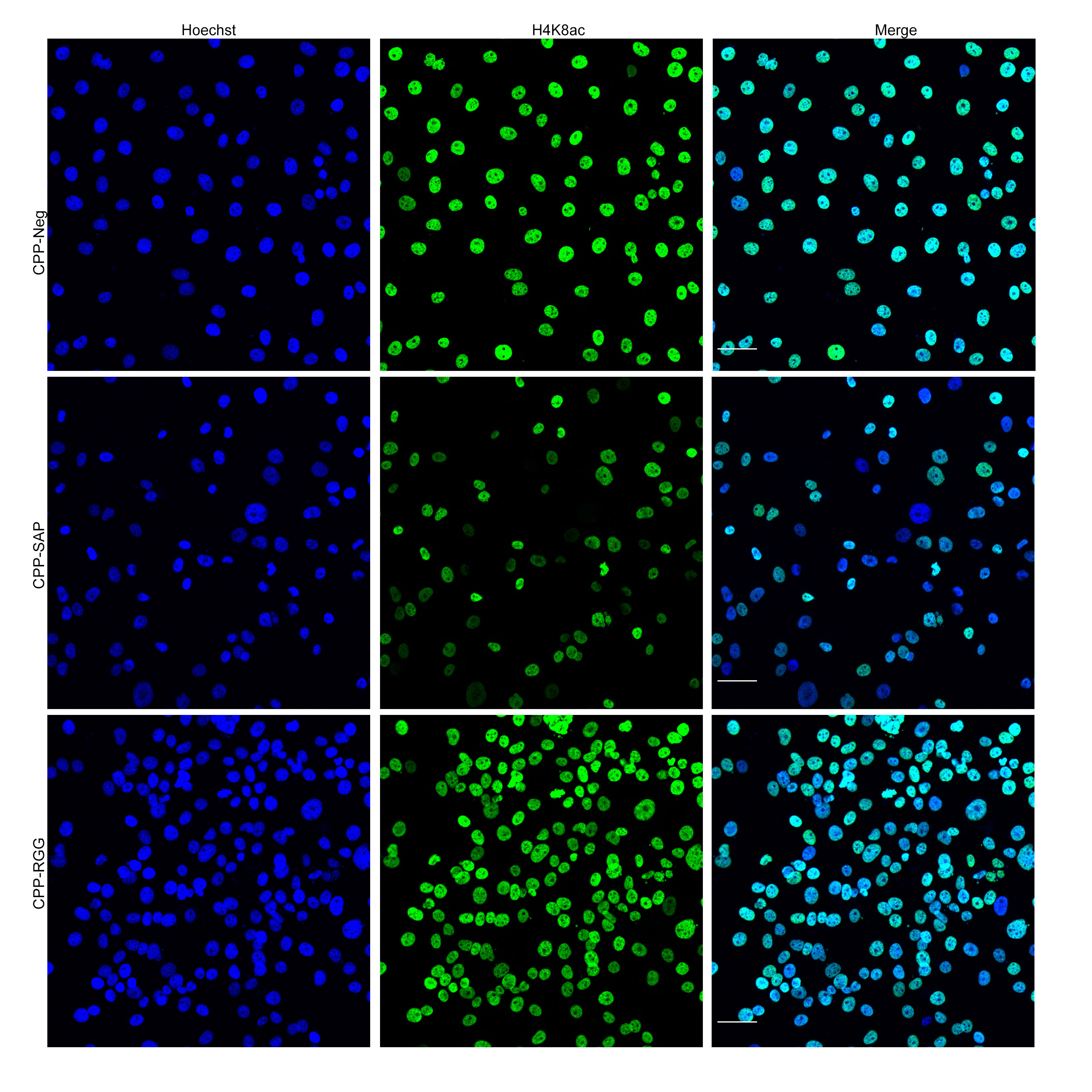

Supplement: Supplementary file 7 [file Data_Sheet_7.zip › Data Sheet 7/S. Fig. 7.3.jpg]

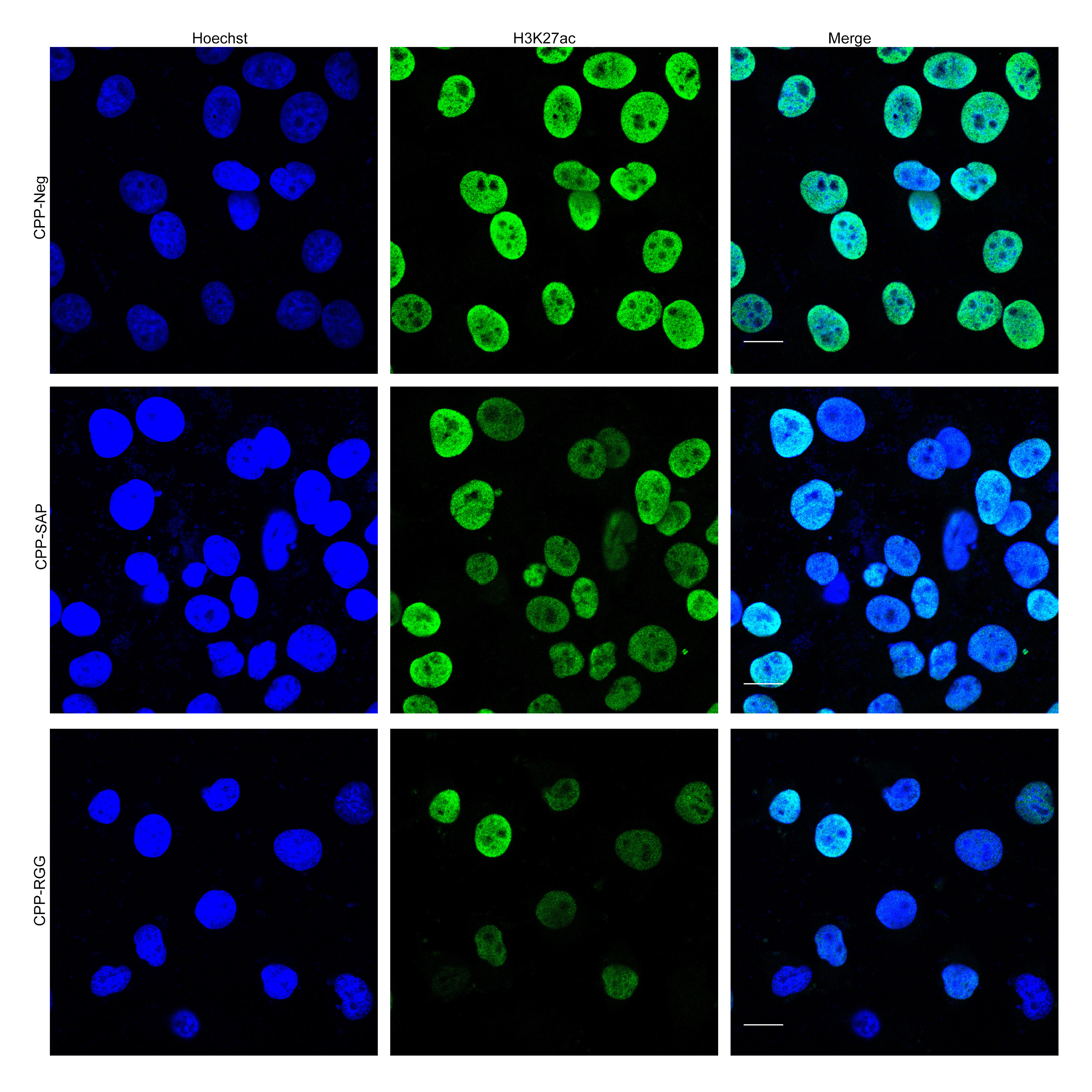

Supplement: Supplementary file 7 [file Data_Sheet_7.zip › Data Sheet 7/S. Fig. 7.16.jpg]

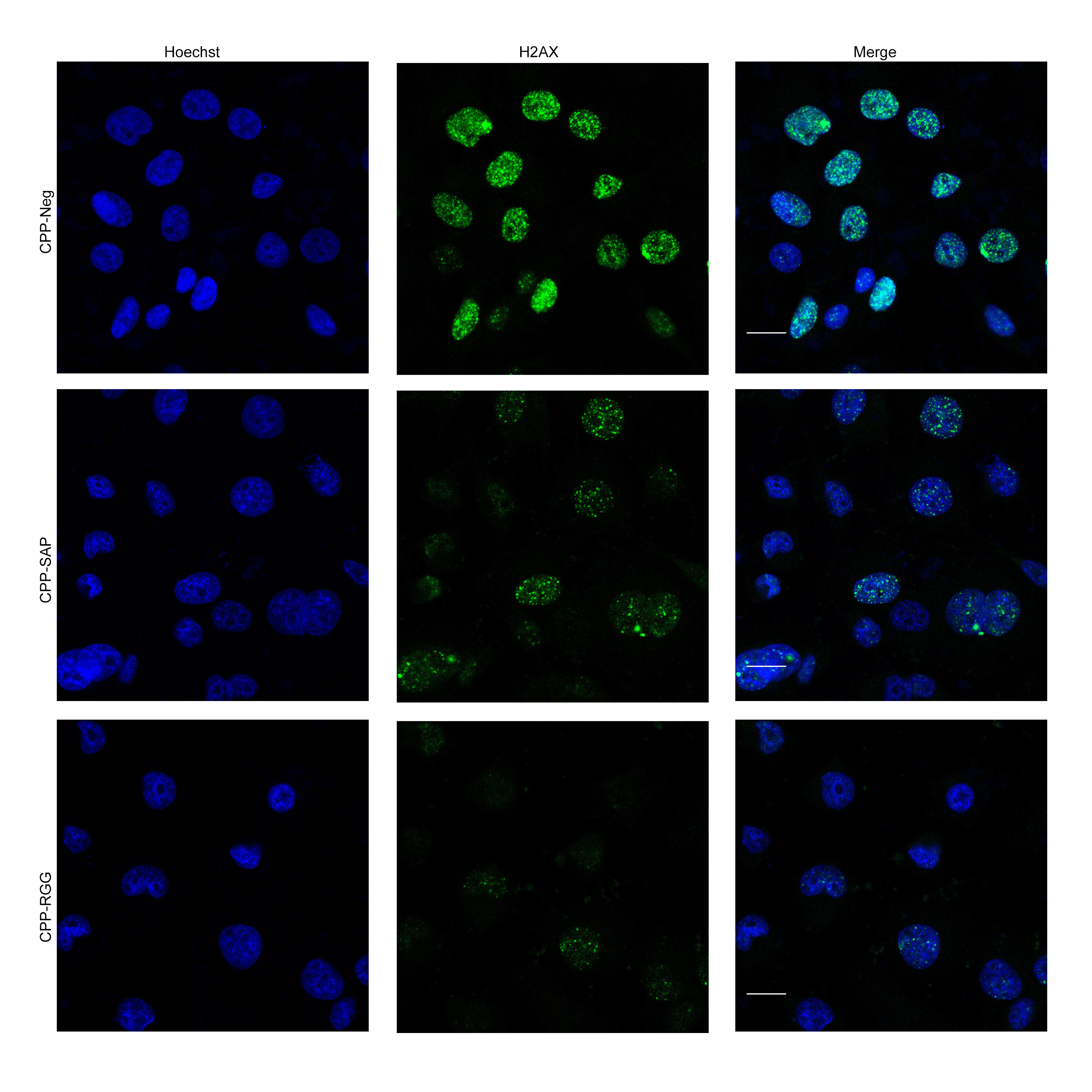

Supplement: Supplementary file 7 [file Data_Sheet_7.zip › Data Sheet 7/S. Fig. 7.2.jpg]

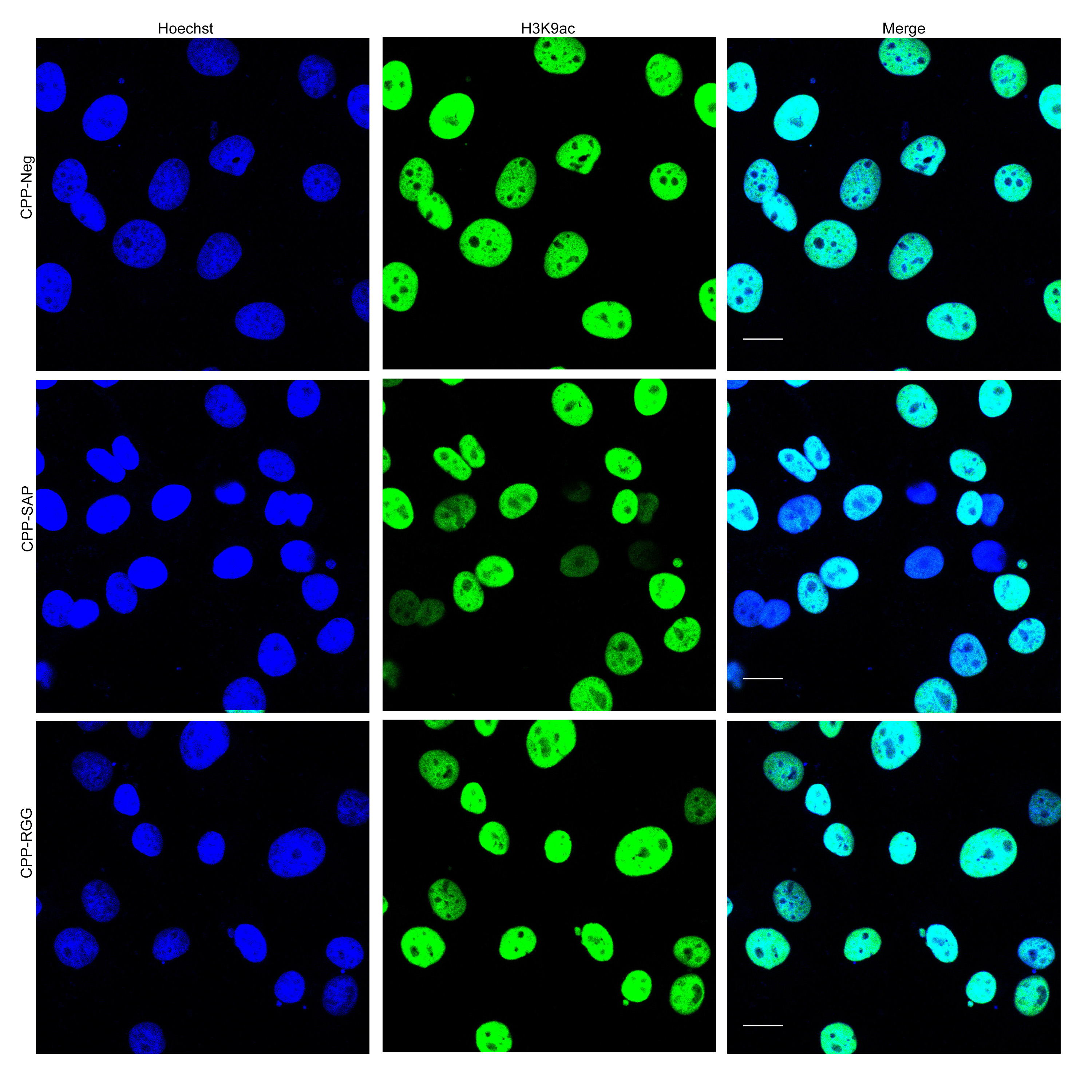

Supplement: Supplementary file 7 [file Data_Sheet_7.zip › Data Sheet 7/S. Fig. 7.6.jpg]

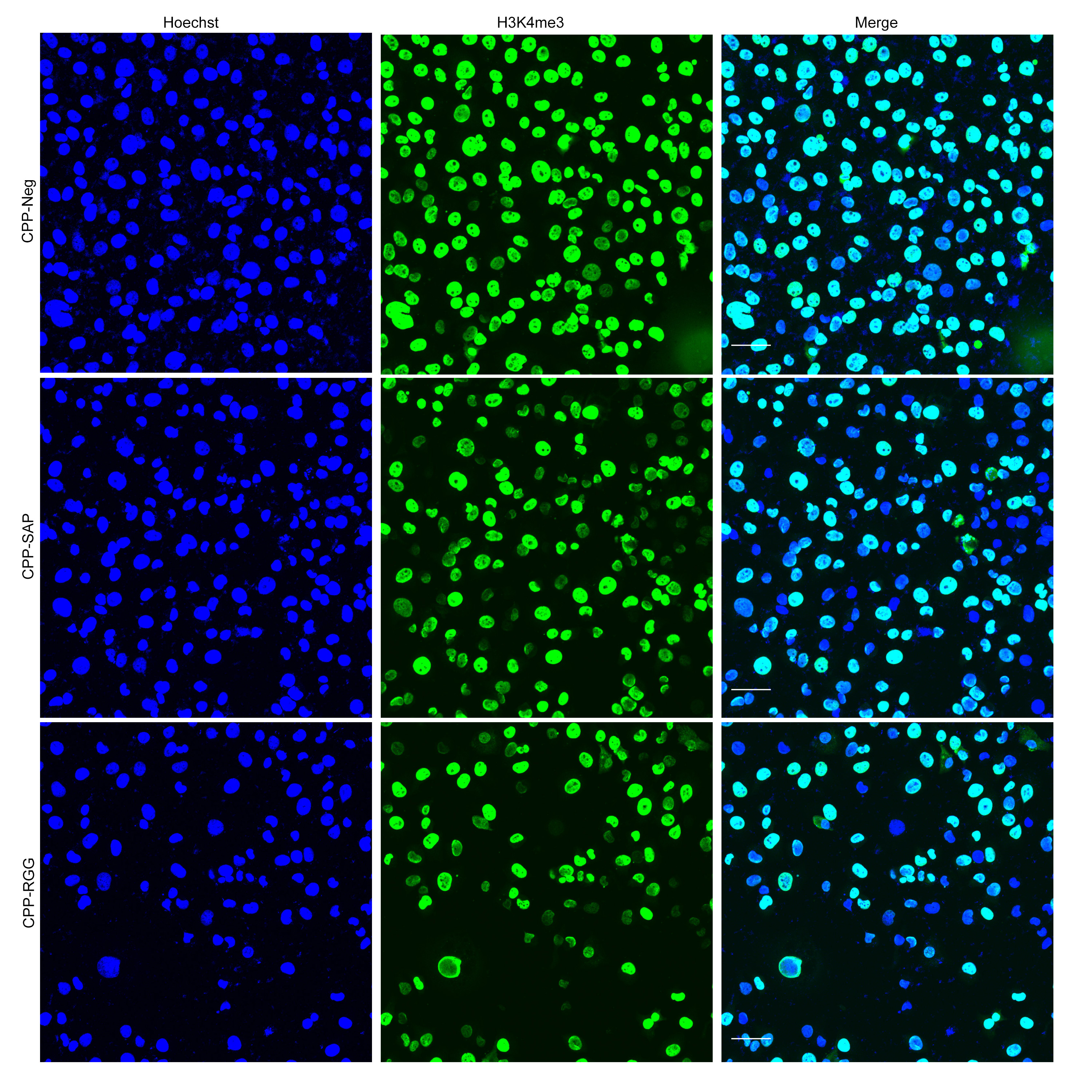

Supplement: Supplementary file 7 [file Data_Sheet_7.zip › Data Sheet 7/S. Fig. 7.13.jpg]

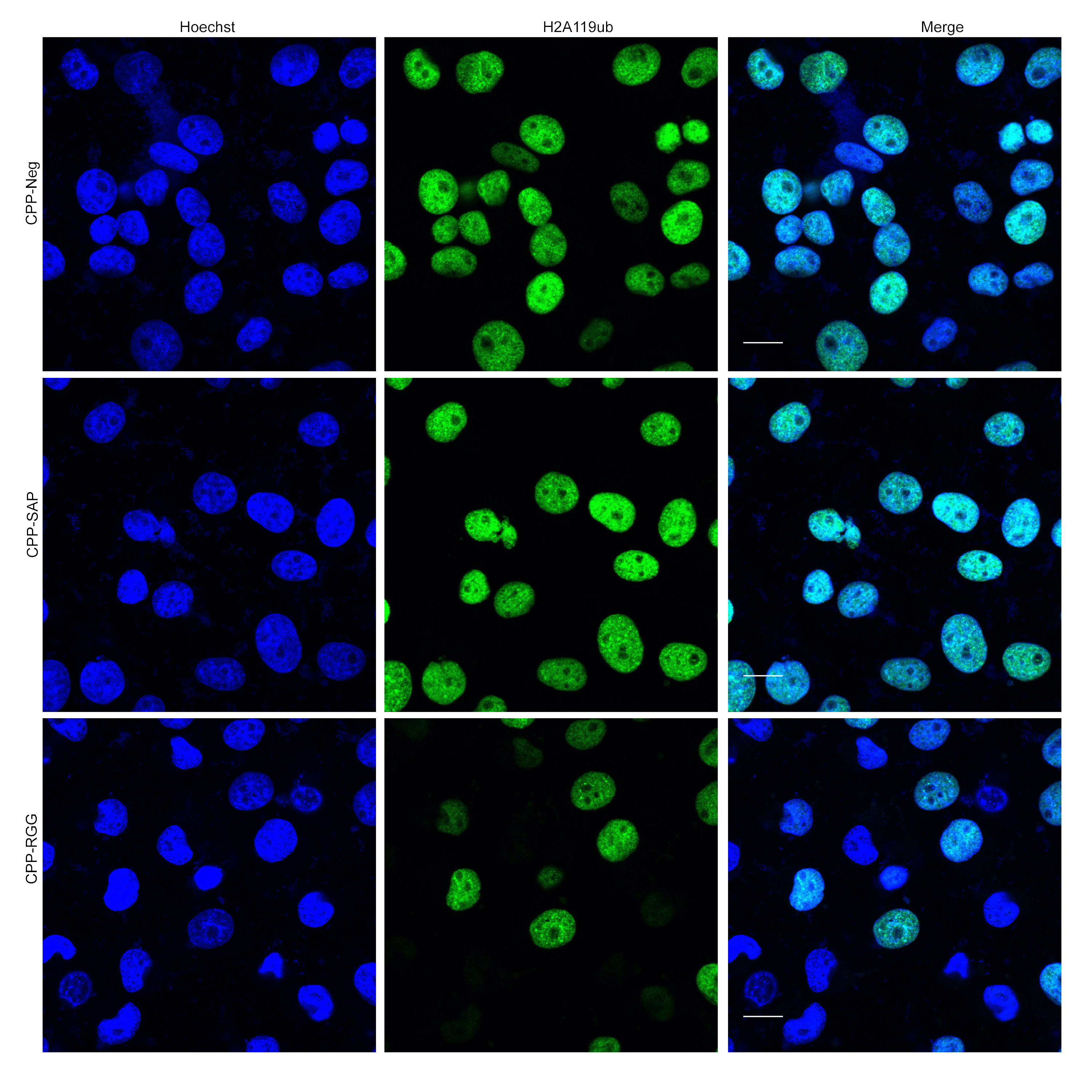

Supplement: Supplementary file 7 [file Data_Sheet_7.zip › Data Sheet 7/S. Fig. 7.12.jpg]

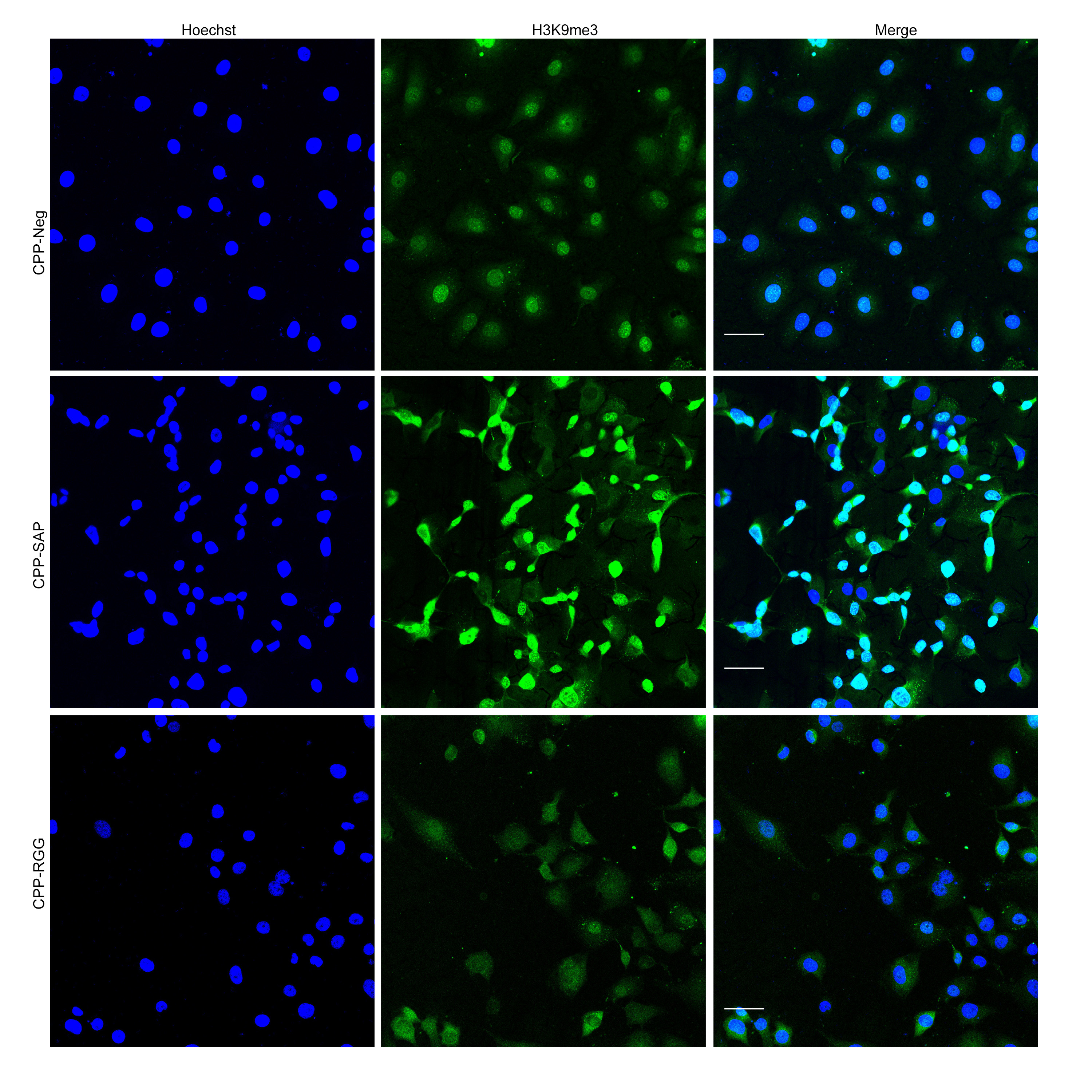

Supplement: Supplementary file 7 [file Data_Sheet_7.zip › Data Sheet 7/S. Fig. 7.7.jpg]

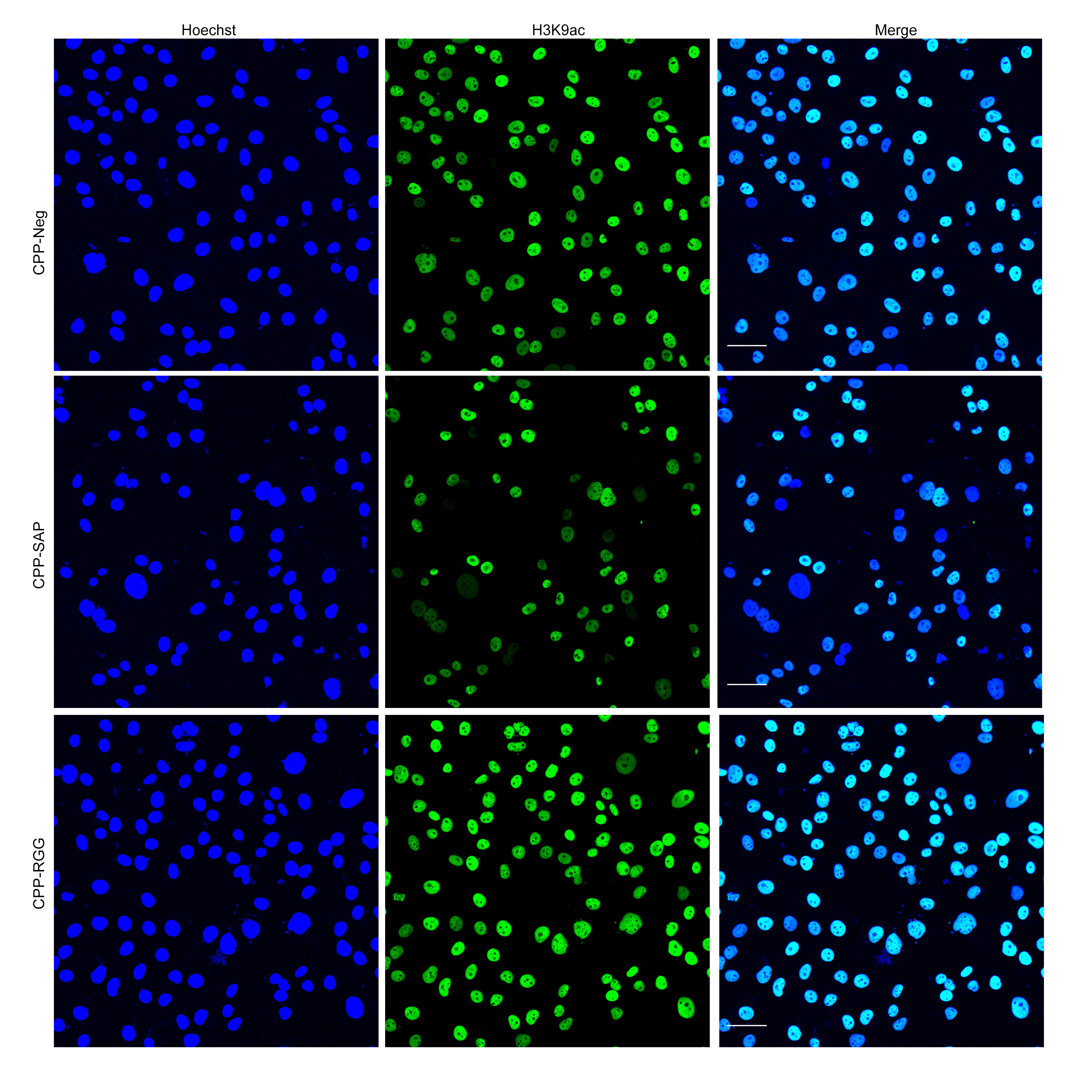

Supplement: Supplementary file 7 [file Data_Sheet_7.zip › Data Sheet 7/S. Fig. 7.5.jpg]

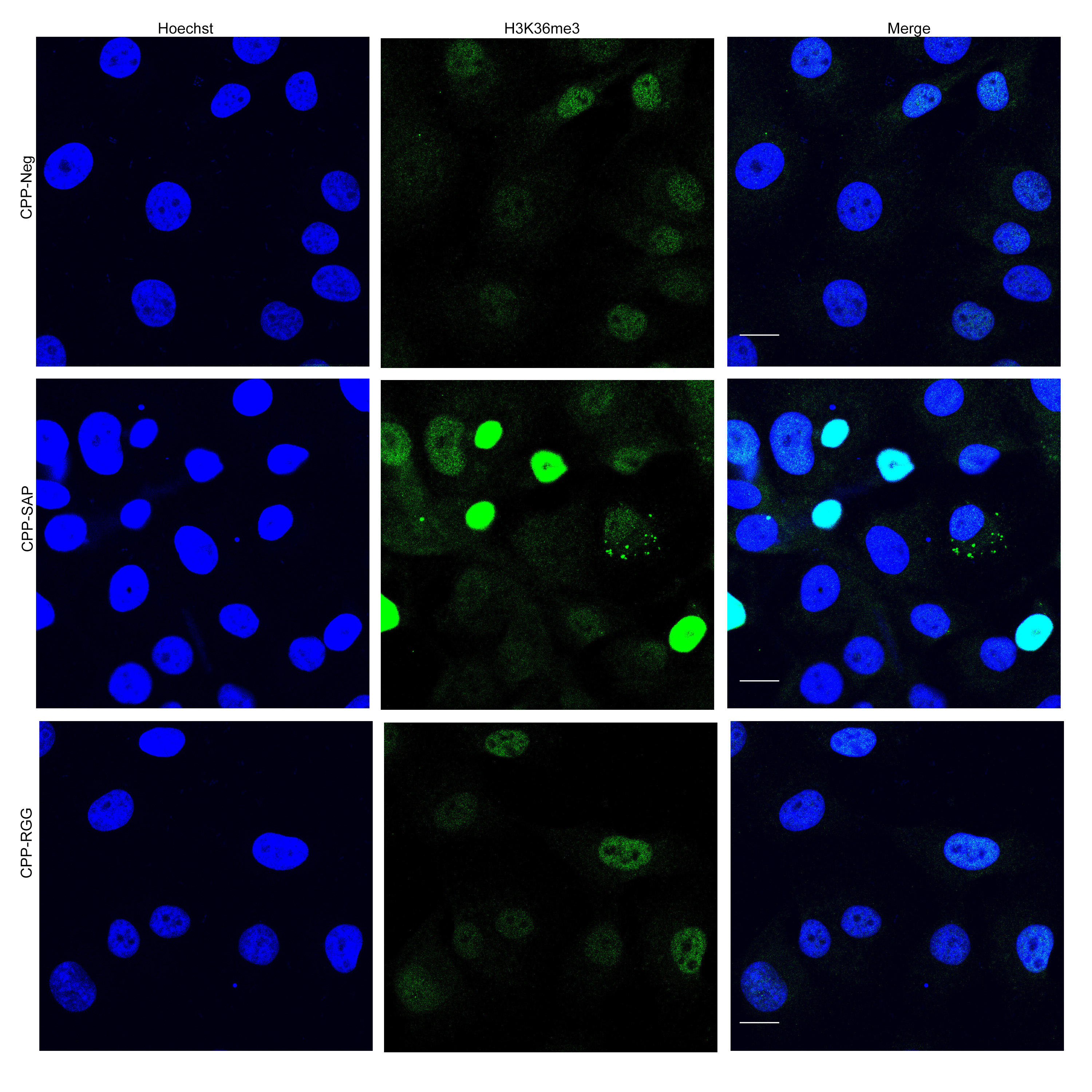

Supplement: Supplementary file 7 [file Data_Sheet_7.zip › Data Sheet 7/S. Fig. 7.10.jpg]

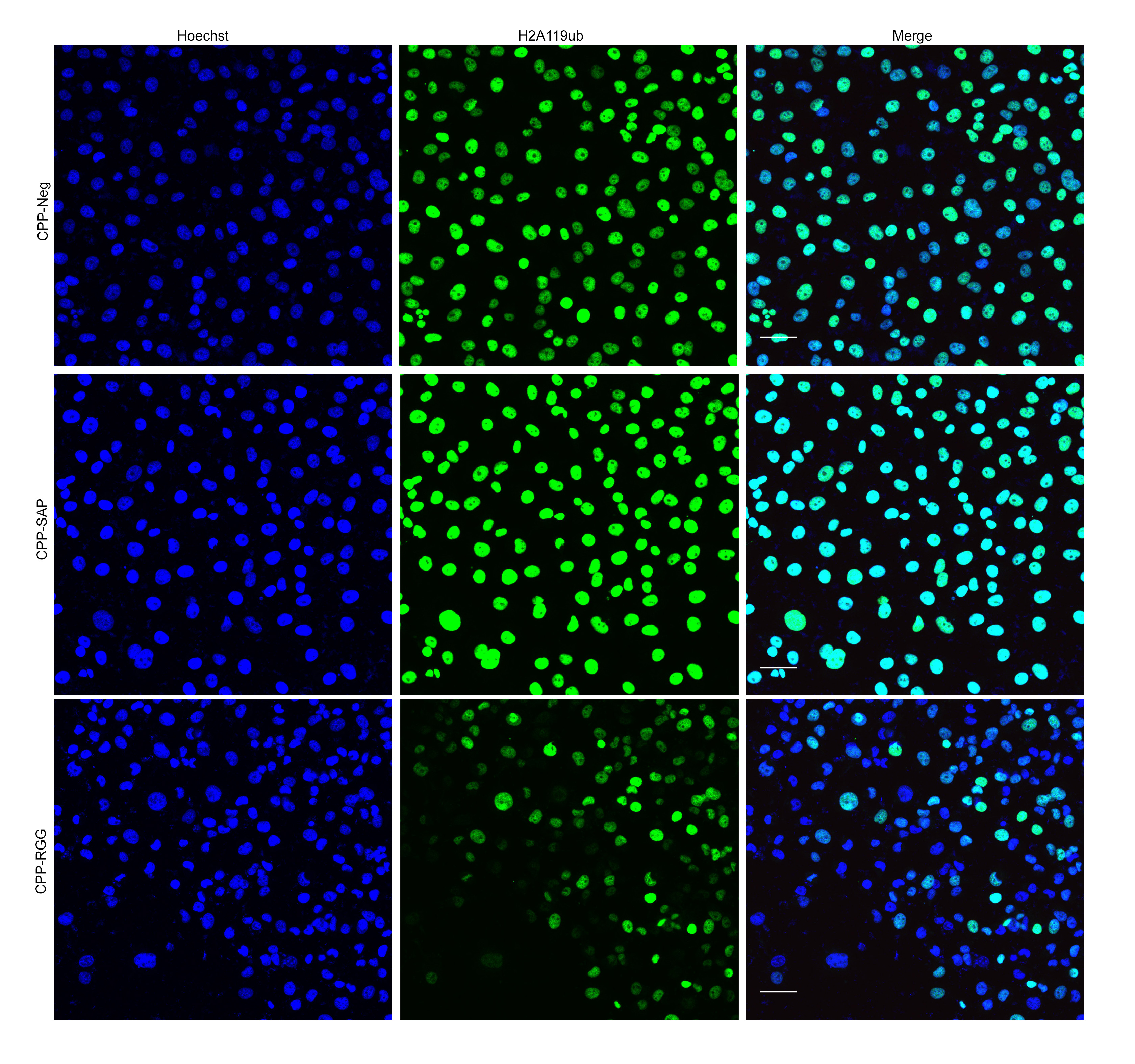

Supplement: Supplementary file 7 [file Data_Sheet_7.zip › Data Sheet 7/S. Fig. 7.11.jpg]

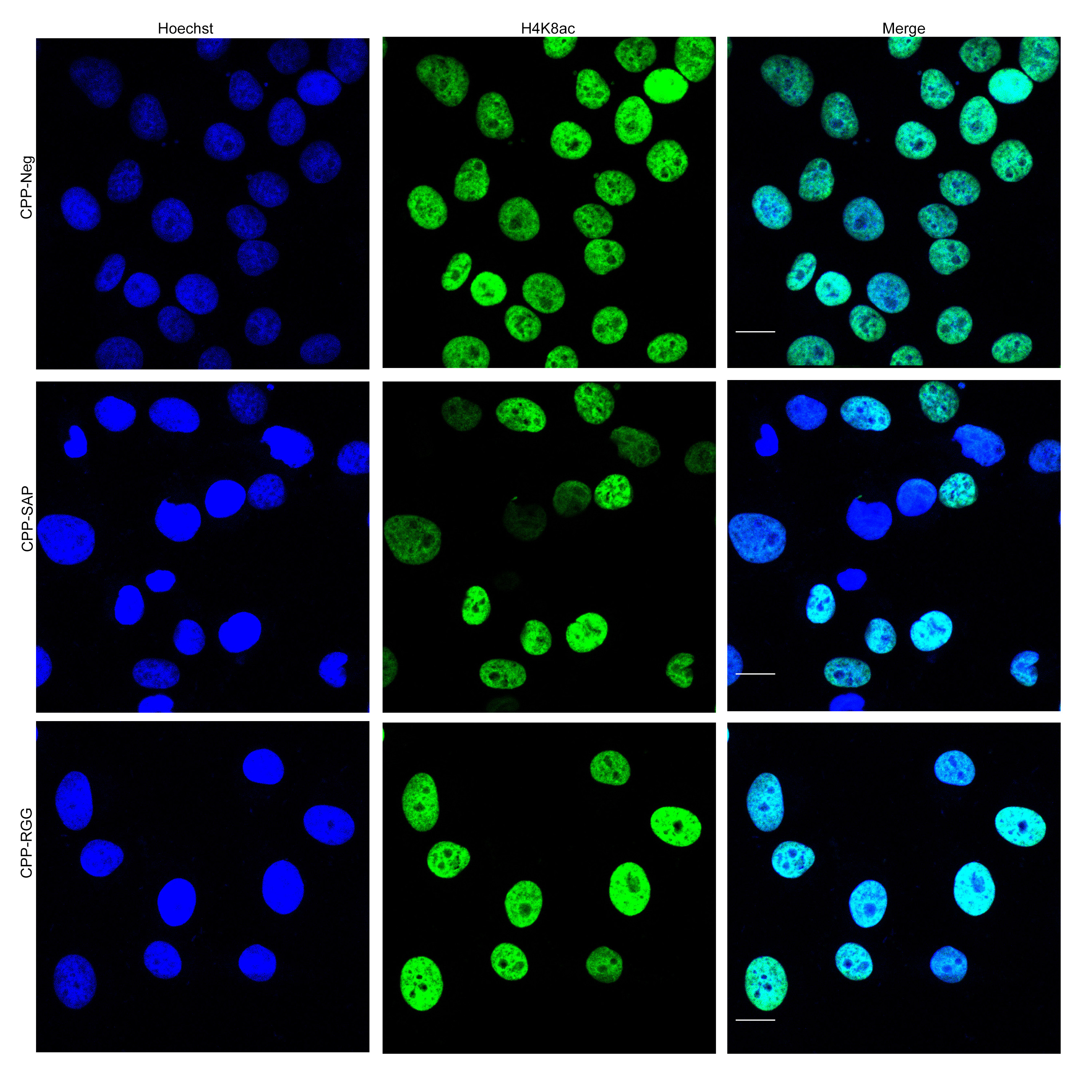

Supplement: Supplementary file 7 [file Data_Sheet_7.zip › Data Sheet 7/S. Fig. 7.4.jpg]
