# Supplementary material for: Novel Cell-Penetrating Peptides Derived From Scaffold-Attachment- Factor A Inhibits Cancer Cell Proliferation and Survival
Source: Front Oncol. 2021 Mar 30;11:621825. doi: 10.3389/fonc.2021.621825 (PMC8042391; doi:10.3389/fonc.2021.621825)
Supplement: Supplementary file 8 [file Data_Sheet_8.docx]

**Supplemental Figure Legends**

**Supplemental Figure 1.1. SAFA-derived cell-penetrating peptides enter nuclei.**

A) Amino acid sequences of the cell-penetrating peptides CPP-Neg, CPP-SAP, CPP-RGG and CPP-Act.

B-E) Representative confocal images of anti-his immunofluorescence signal of HFFs treated with synthetic his6-tagged CPP-Neg, CPP-SAP, CPP-RGG and CPP-Act peptides for 4 hours. Left panel represents Alexa Fluor 596-anti-his signal (red), middle panel is Hoechst (blue) and the right panel corresponds to the merged image. Scale bar, 10 μm

F-J) Representative anti-his immunofluorescence of untreated HFFs, HCT116 and MDA-MB231 cells. F and G scale bars are 10 μm, H scale bar is 50 μm. I and J panels are no primary antibody negative controls. Scale bar, 50 μm.

**Supplemental Figure 1.2. CPP-Act peptide does not affect cell proliferation.**

A) Amino acid sequences of CPP-Neg and CPP-Act.

B-G) Representative light microscopic images of crystal violet stained adherent cells treated with peptide listed at top for 48 hours.

**Supplemental Figure 2.1. SAFA-derived peptides penetrate into multiple types of cancer cells.**

A) Peptide amino acid sequences

B-G) Representative confocal images of anti-his immunofluorescence signal in cells treated with synthetic peptides for 4 hours.

**Supplemental Figure 2.2. SAFA-derived peptides induce cell death in breast cancer cells.**

Representative confocal images of Annexin V / PI stained cells after 24 hours of peptide treatment. Individual channels (Hoechst=Blue, Annexin V=Green, PI=Red) and merged images are shown for each treatment. Scale bar, 50 μm

**Supplemental Figure 2.3. SAFA-derived peptides induce death of multiple cancer cell lines.**

Representative immunofluorescence images of cancer cells treated for 24 hours.

**Supplemental Figure 2.4. SAFA-derived peptides disrupt cell cycle progression.**

Flow cytometry results cells from cells treated with peptides for 24 hours. The percentage of cells in G, S and G2 is indicated in the right top corner of the panel.

**Supplemental Figure 2.5. SAFA-derived CPPs reduce expression of cell cycle genes in cancer cells.**

qRT-PCR analysis of cell cycle genes in total RNA isolated from cell lines listed after 24 hours of peptide treatment. * indicates p<0.05 and ** indicates p<0.01 relative to control.

**Supplemental Figure 3.1. RT-PCR showing SAFA splicing targets that are unaffected by peptide treatment.**

A-F) Representative agarose gels of RT-PCR assay on SAFA splicing targets in cell types noted at top.

**Supplemental Figure 3.2. Assay of SAFA splicing targets in MCF10A cells in response to peptide treatment.**

A, B) Representative agarose gel pictures of RT-PCR assays.

**Supplemental Figure 4.1. Variable effects of CPP-RGG and CPP-SAP on SAFA association with RNA targets with peptide-independent splicing.**

A-D) Representative agarose gel pictures of Crosslinked RNA-IP (CLIP) of cells treated with peptides. Antibodies and cell lines are indicated at top. Transcripts are indicated at left. Compare with peptide sensitive targets in Figure 4A-D.

**Supplemental Figure 4.2**. Comparison of single antibody staining of HCT116 cells treated with SAFA-derived peptides. Peptide treatments are indicated at left. Peptide treated and fixed HCT116 cells were stained with specific antibodies against the indicated proteins. Images were taken using 20X dry (scale bar: 50μm) and 60X oil-immersion lenses. (scale bar: 10μm). Images Supplemental Figure 4.2: 1, 3, 5, 7, 9, 11, 13, 15, 17 were taken using 20X and Supplemental Figure 4.2: 2, 4, 6, 8, 10, 12, 14, 16, 18 were taken using 60X lens.

Supplemental Figure 4.2, 1 and 2: Effect of peptides on DDX3 expression and localization.

Supplemental Figure 4.2, 3 and 4: Effect of peptides on DDX21 expression and localization

Supplemental Figure 4.2, 5 and 6: Effect of peptides on hnRNPA1 expression and localization

Supplemental Figure 4.2, 7 and 8: Effect of peptides on hnRNPC1 expression and localization

Supplemental Figure 4.2, 9 and 10: Effect of peptides on hnRNPD expression and localization

Supplemental Figure 4.2, 11 and 12: Effect of peptides on SAFA expression and localization

Supplemental Figure 4.2, 13 and 14: Effect of peptides on SC35 expression and localization

Supplemental Figure 4.2, 15 and 16: Effect of peptides on TDP43 expression and localization

Supplemental Figure 4.2, 17 and 18: Effect of peptides on U2AF65 expression and localization

**Supplemental Figure 4.3. CPP-SAP and CPP-RGG do not affect SAFA association with target promoter regions in MDA-MB231 or HCT116 cells.**

A, B) ChIP-qPCR for indicated gene promoters after 24 hours treatment. Y-axis indicates fold enrichment relative to input.

**Supplemental Figure 5**.

A) ChIP-PCR of CDK1, CCNB1 and CDC25C promoters with R-IgG and SAFA in MDA-MB231 and HCT116 cells. B) ChIP-PCR of CDK1, CCNB1 and CDC25C promoters with R-IgG and SAFA in MDA-MB231 cells treated with CPP-Neg, CPP-SAP and CPP-RGG peptides for 24hourss.

**Supplemental Figure 6.1**. Comparison of endogenous levels of DNA damage response factors and nuclear proteins in HCT116 cells treated with SAFA-derived peptides by using single antibody staining against indicated proteins. Peptide treatments are indicated at left. Images were taken using 20X dry (scale bar: 50μm) and 60X oil-immersion lenses. (scale bar: 10μm). Images Supplemental Figure 6.1.1, 3, 5, 7, 9, 11, 13, 15, 17, 19, 21, 22 were taken using 20X and Supplemental Figure 6.1.2, 4, 6, 8, 10, 12, 14, 16, 18, 20, 23 were taken using 60X lens.

Supplemental Figure 6.1, 1 and 2: Effect of peptides on p-ATR expression and localization.

Supplemental Figure 6.1, 3 and 4: Effect of peptides on p-CHK2 expression and localization

Supplemental Figure 6.1, 5 and 6: Effect of peptides on p-ATM expression and localization

Supplemental Figure 6.1, 7 and 8: Effect of peptides on p-CHK1 expression and localization

Supplemental Figure 6.1, 9 and 10: Effect of peptides on ASH2L expression and localization

Supplemental Figure 6.1, 11 and 12: Effect of peptides on C23 expression and localization

Supplemental Figure 6.1, 13 and 14: Effect of peptides on Lamin A/C expression and localization

Supplemental Figure 6.1, 15 and 16: Effect of peptides on Lamin B1 expression and localization

Supplemental Figure 6.1, 17: Effect of peptides on MLL1 expression and localization

Supplemental Figure 6.1, 18 and 19: Effect of peptides on PML expression and localization

**Supplemental Figure 6.2**. Effect of peptides on caspase 3, 7 and 9 activation in HCT116 and MDA-MB231 cells. A-C) Immunofluorescence for caspase 3, 7 and 9 in CPP-Neg, CPP-SAP and CPP-RGG treated HCT116 cells. Scale bar, 10 μm

D, E) Caspase-Glo 3/7 assay. X and Y-axis indicate the incubation of the peptide treatment and luminescence units respectively.

**Supplemental Figure 7**. Comparison of histone epigenetic marks in HCT116 cells treated with SAFA-derived peptides by using single antibody staining against indicated proteins. Peptide treatments are indicated at left. Images were taken using 20X dry (scale bar: 50μm) and 60X oil-immersion lenses. (scale bar: 10μm). Images Supplemental Figure 7: 1, 3, 5, 7, 9, 11, 13, and 15 were taken using 20X and Supplemental Figure 7: 2, 4, 6, 8, 10, 12, 14, and 16 were taken using 60X lens.

Supplemental Figure 7.1 and 2: Effect of peptides on γH2AX expression and localization.

Supplemental Figure 7.3 and 4: Effect of peptides on H4K8ac expression and localization

Supplemental Figure 7.5 and 6: Effect of peptides on H3K9ac expression and localization

Supplemental Figure 7.7 and 8: Effect of peptides on H3K9me3 expression and localization

Supplemental Figure 7.9 and 10: Effect of peptides on H3K36me3 expression and localization

Supplemental Figure 7.11 and 12: Effect of peptides on H2A119ub expression and localization

Supplemental Figure 7.13 and 14: Effect of peptides on H3K4me3 expression and localization

Supplemental Figure 7.15 and 16: Effect of peptides on H3K27ac expression and localization.

**Table 1:** Primer sequences used for the RT-PCR and ChIP-PCR analysis.

| Name | Sequence |
| --- | --- |
| ABI2 | GCCATACTCCCCCAACAATA |
| ABI2 RP | GTGGGGGAGACTCATCAAAG |
| *ASPH* | TCGAAGATGAAGCAAAAGAACA |
| *ASPH RP* | CTTCCACGTGGTAACTATGCTC |
| ATXN2 | CAACTCAGTACGGGGCTCAT |
| ATXN2 RP | GACTGGGTGCAGGATGACTT |
| CDC42BPA | GAGCACCAAGACTCACAGCA |
| CDC42BPA RP | GTGGAAAGCCAGTTGAACCA |
| ECHDC1 | AGAGGGGAAAGGCCTCATT |
| ECHDC1 RP | ATTCTGCTCCTCCACCCAAT |
| EIF4A2 | TGTGCAACAAGTGTCTTTGG |
| EIF4A2 RP | TCAATGTCACGAAGAATCCTCT |
| FIP1L1 | GAGGATACGAATGGGACTTGA |
| FIP1L1 RP | TGGAAGCCCAGTCTTGAACA |
| *GAPVD1* | ACAGGAACAGACCTTGGTGG |
| *GAPVD1 RP* | CCGTTTAATGGCATTCCTGT |
| *KITLG* | TCATTCAAGAGCCCAGAACC |
| *KITLG RP* | GCCCAGTGTAGGCTGGAGT |
| *KRIT1* | ATCTCGGTGGTCCAACTCAG |
| *KRIT1 RP* | TGGCAGTATTCTTTGGACGA |
| MACF1 | CCTACTCGTTCCAGCTCCAG |
| MACF1 RP | GCAAGGGATGTCCGACTAGA |
| MAP3K7 | CTCCATCCCAATGGCTTATC |
| MAP3K7 RP | TTTTTGCATTGCTGGTAGTAAG |
| *MFF* | CGAGCAGTTGGCAGACTAAA |
| *MFF RP* | ATGAGGATTAGAAGTGGCGG |
| ODF2L | GGCTGTAAATGATGGAAGTCA |
| ODF2L RP | TTTCCTTCTGTTCAGAAATCTTCA |
| PICALM | TCAAAGCTGCCCAATGATCT |
| PICALM RP | ACATTAAGGCCAGCTGAAGG |
| *PLCXD2* | TTGGATCATCTAAACCGGAAA |
| *PLCXD2 RP* | AACTTCACGCAGGAGCTGTT |
| PRRC2B | ATGAAAGGCTTCCACTTTGC |
| PRRC2B RP | TCAGGCCTTACTCTCCTCCA |
| PSMG1 | GTCTGGGAGGAAGTTGGTTG |
| PSMG1 RP | TGCATGTTCTTCCTTGGACA |
| SS18 | GTGGACAAGGTCCTCCAGAA |
| SS18 RP | CTGCTGCTGTCCTGGGTAAC |
| STARD3NL | CTTCTGGCAGTTTTTCGATTT |
| STARD3NL RP | CAGGAACCACGTCTCAATCC |
| *STRAP* | GTTGATTTGGCCTTCAGTGG |
| *STRAP RP* | GCGTGAAATCCACAGTCTTG |
| TBC1D15 | GAAGCAGAATGGGACATGGT |
| TBC1D15 RP | CCATATGCTGGCTCATCAAG |
| *WDHD1* | TCTTGTGGATCCTCAGTGGC |
| *WDHD1 RP* | CATCATCATCCAAGTCTTCCC |
|  |  |
| AURKA | CTGCCTTGTGATTGGTTGAC |
| AURKA RP | CACCACTTCCGGGTTCTTA |
|  |  |
| CCNB1 | GCCACGAACAGGCCAATAA |
| CCNB1 RP | AGAAACCAACAGCCGTTCC |
|  |  |
| CDK1 | GGGGATTCAGAAATTGATCA |
| CDK1 RP | TGTCAGAAAGCTACATCTTC |
|  |  |
| CDC25C | GAATGGACATCACTAGTAAGGCGCG |
| CDC25C RP | GCAGGCGTTGACCATTCAAACCTTC |
|  |  |
| CDC2 | TCTCTAGCCGCCCTTTCCTCTTTCTTTC |
| CDC2 RP | CTTTGAAGCCAAGTGCGAGCAGTTTC |
|  |  |
| CCNE1 | AAATGGCCAAAATCGACAGG |
| CCNE1 RP | CGAGGCTTGCACGTTGAGTT |
|  |  |
| SAFA | GAGCATCCTATGGTGTGTCAAA |
| SAFA RP | TGACCAGCCAATACGAACTTC |
|  |  |
| CCNA2 | AGTAAACAGCCTGCGTTCACC |
| CCNA2 RP | GAGGGACCAATGGTTTTCTGG |
|  |  |
| CENPF | CGAAGAACAACCATGGCAACTCG |
| CENPF RP | TTCTCGGAGGATGGTGCCTGAAT |
|  |  |
| E2F1 | ACTCCTCGCAGATCGTCATCATCT |
| E2F1 RP | GGACGTTGGTGATGTCATAGATGCG |
|  |  |
| HPRT | GCTGGTGAAAAGGACCTCT |
| HPRT RP | CACAGGACTAGAACACCTGC |
|  |  |
|  |  |
| siRNA SAFA |  |
|  |  |
| S102781002: CTGGCCGTGGTAGTTACTCAA |  |
| S102780540: AGGATATTATTGAATACCCAA |  |
|  |  |
|  |  |
| ChIP-Primers |  |
|  |  |
| MT1G | AGATGGGCCAAGTGCAAG |
| MT1G RP | GTGAGAGAAGCCGCACAC |
|  |  |
| PELI1 | CGGTCTTGTATTATGGTGTTGTTG |
| PELI1 RP | TCAGACCTCACTGGGTACTTAT |
|  |  |
| BIRC3 | CCCTACATTTCCTTTCACCTCTTAC |
| BIRC3 RP | GCTGCAGAAGTCCAGCATTTA |
|  |  |
| BDNF | TTCAGAAACCCTCACAGTCATC |
| BDNF RP | AGCATCTGCTGGCTAATTCA |
|  |  |
| MMP3 | GAGAGAAGAAGTAGGTTGACTTGG |
| MMP3 RP | AGCTATGTATGTACACTTTCCACTT |
|  |  |
| PKP1 | TGGTCTCTGCTATGACCTCAG |
| PKP1 RP | GGAGACCTCTCTCTAGGAAAGAAA |
|  |  |
| JUNB | CAATGGATTGTCAGTCCTCCTAC |
| JUNB RP | GGAGTCCACTGGGACAAATAC |
|  |  |
| RASD1 | CTGTCCCTCTGCGACTTCGG |
| RASD1 RP | ACTGGGTTACTTACTAGATTCCTATTC |

Rationale for selecting different cell line and targets:

We previously demonstrated that SAFA knockdown induces senescence in primary human fibroblasts by dysregulating cell cycle gene expression. Therefore, for proof-of-principle testing of the dominant-negative effects of SAFA-derived peptides, we chose these cells. Both SAFA knockdown from our previous study and the SAFA-derived peptide treatment from this study evoke similar phenotypic responses. Since SAFA knockdown induces senescence in fibroblasts, a key tumor suppressor mechanism, we next assayed for dominant-negative domain effects in breast cancer cell lines. We chose one normal mammary epithelial and three breast cancer cell lines representing different subtypes and evaluated how SAFA-derived peptides impact cell-proliferation, apoptosis, and gene expression. Furthermore, SAFA is a ubiquitous protein in normal cells so we expanded to other cancer cell lines of different tissue origins (HCT116 (colorectal carcinoma), DU145 (prostate cancer), UMUC3 (bladder cancer), and HT1080 (fibrosarcoma cell line). Such a wide range of panels demonstrates the peptides’ broad-spectrum usage and specificity.

For mechanistic understanding of effects on gene expression and splicing, we selected predominantly published targets because our goal was to determine how these peptides are affecting the SAFA’s function rather than discovering novel target genes in this study. Hence, we selected all the previously published SAFA-deregulated genes for the cell cycle gene expression and used them in the RT-PCR assay. We mined the published literature for splicing targets and selected a set of splicing genes directly affected by the SAFA knockdown. We validated them in fibroblasts and confirmed that SAFA regulates the splicing of some of the targets. Of this, we randomly selected 20 targets and evaluated them in 6 different cell types. By choosing targets from different cell types provides evidence for SAFA’s function in regulating its direct splicing targets in multiple cellular contexts.

For the initial characterization of peptide treatments, we used nine cell lines. Later, we selected two breast cancer and four different cancer cell types and performed splicing assays to see the peptides’ effect on SAFA targets. From there, we selected four cell lines to test whether CPP-RGG peptide treatment reduces the SAFA association with its known targets. As proof of principle, one cell line is sufficient. But showing the effect of RGG on SAFA binding in different cell lines gives confidence in its action.

We selected the HCT116 cell line for immunofluorescence analysis as it responded well to CPP-SAP and to CPP-RGG. Secondly, it was an exploration of how these peptides affect different cellular proteins that play a role in nuclear structure, nuclear bodies, and epigenetic marks.

Finally, for understanding the CPP-SAP effect on higher-order chromatin, we selected four different cell types. We show how CPP-SAP treatment affecting the chromatin compaction in cancer but not normal epithelial cells. We tried to incorporate more than four cell types for any experiment throughout the paper except for the immunofluorescence study to provide confidence in the peptides’ mechanisms.
